# Supplementary material for: Hypoxia‐inducible factor 1A inhibition overcomes castration resistance of prostate tumors
Source: EMBO Mol Med. 2023 Apr 18;15(6):e17209. doi: 10.15252/emmm.202217209 (PMC10245031; doi:10.15252/emmm.202217209)
Supplement: Supplementary file 1 — Expanded View Figures PDF [file EMMM-15-e17209-s005.pdf]

Expanded View Figures

Figure EV1. Characterization of castrated Pten$^{(i)pe-/-}$ mice.

A–C Weight of bulbocavernosus muscles (A), seminal vesicles (B), and prostates (C) of Pten$^{L2/L2}$ (control) and Pten$^{(i)pe-/-}$ mice, sham-operated (sham), or castrated (CTX) at 3 months AGI and analyzed at 1 month after surgery. $N = 3–12$ mice/condition. Data presented are mean ± SEM. *P*-values were determined by one-way ANOVA followed by a *post hoc* Tuckey test.

D Gland areas in DLP of sham and CTX control Pten$^{L2/L2}$ and Pten$^{(i)pe-/-}$ mice. $N = 3$ mice/condition. Five glands per mouse were quantified. *P*-values were determined by one-way ANOVA followed by a *post hoc* Tuckey test.

E UMAP depicting the unbiased clusters obtained from scRNA-seq analysis of cells from prostates of Pten$^{(i)pe-/-}$ mice, sham or CTX at 3 months AGI and analyzed 1 month after surgery.

F Stacked bars showing the proportion of the identified cell populations in the prostates of sham and CTX Pten$^{(i)pe-/-}$ mice, based on the scRNA-sequencing analysis.

G Feature plots depicting the expression of Epcam, Ptprc, and Vim in the various cell clusters.

H UMAP depicting luminal-A and luminal-C cells obtained from scRNA-seq analysis of prostates of Pten$^{(i)pe-/-}$ mice, sham or CTX at 3 months AGI and analyzed 1 month after surgery.

© 2023 The Authors

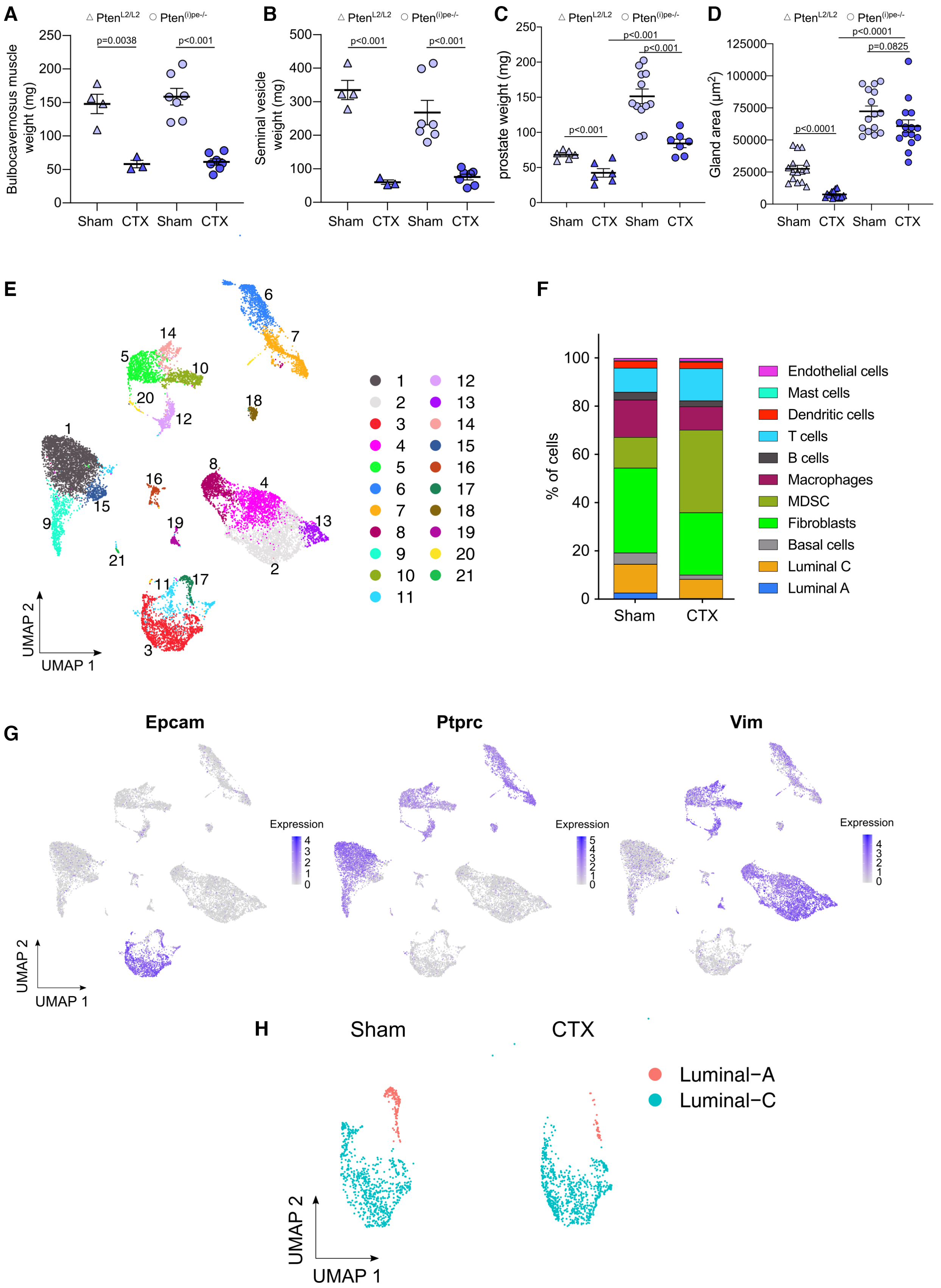

Figure EV1.

 © 2023 The Authors

Figure EV2. Characterization of castrated Pten$^{(i)pe-/-}$ and Pten/Hif1a$^{(i)pe-/-}$ mice.

A, B Seminal vesicles weight of Pten$^{(i)pe-/-}$ mice (A) and Pten/Hif1a$^{(i)pe-/-}$ mice (B), sham-operated (sham), or castrated (CTX) at 3 months AGI and analyzed 3 days after surgery. (A) $N = 3$ mice/condition. (B) $N = 3$–4 mice/condition. Data presented are mean ± SEM. *P*-values were determined by one-way ANOVA followed by a *post hoc* Tuckey test.

C Representative immunohistochemical detection of AR in the DLP of Pten$^{(i)pe-/-}$ and Pten/Hif1a$^{(i)pe-/-}$ mice 1 day after sham operation, and 1–3 days after castration, performed 3 months AGI. Scale bars: 100 μm. $N = 3$ mice/condition.

D, E Percentage of Ki67 positive epithelial cells in DLP of Pten$^{(i)pe-/-}$ mice (D) and Pten/Hif1a $^{(i)pe-/-}$ mice (E) 1 day after sham operation and 1, 2, 3 or 30 days after castration, performed at 3 months AGI. $N = 3$–4 mice/condition. Five glands per mouse were quantified. *P*-values were determined by unpaired *t*-tests.

F–H Representative immunohistochemical detection of cleaved PARP (CPARP) in DLP of sham and CTX Pten$^{(i)pe-/-}$ and Pten/Hif1a$^{(i)pe-/-}$ mice (F) and quantification of CPARP-positive prostatic epithelial cells in DLP of Pten$^{(i)pe-/-}$mice (G) and Pten/Hif1a$^{(i)pe-/-}$ (H) mice. Surgery was performed at 3 months AGI. Sham mice were analyzed 1 day later and CTX mice 1, 2, 3, and 30 days later. $N = 4$ mice/condition. (F) Scale bars: 100 μm. Five glands per mouse were quantified in (G) and (H). Data presented are mean ± SEM. *P*-values were determined by unpaired *t*-tests.

© 2023 The Authors

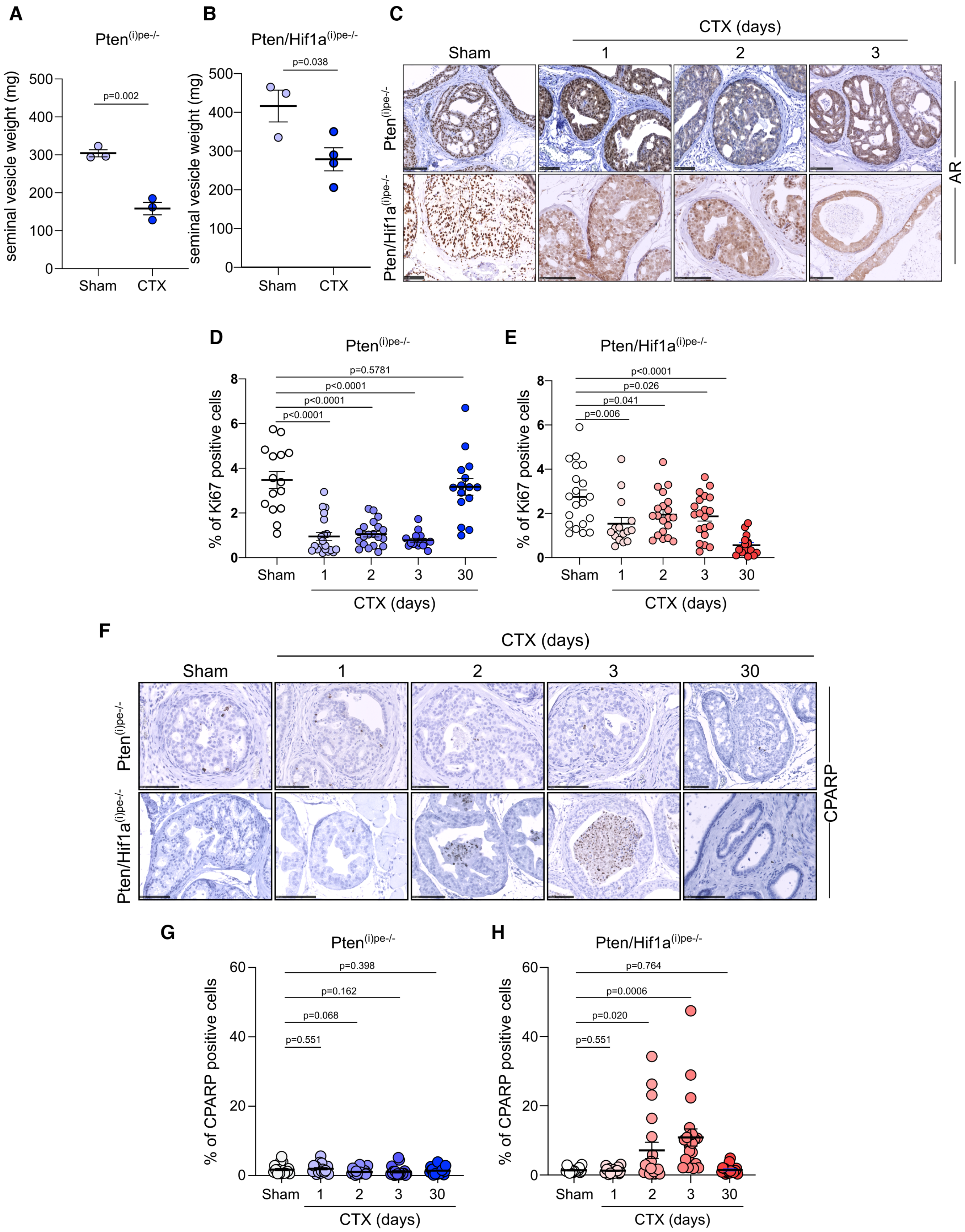

Figure EV2.

© 2023 The Authors

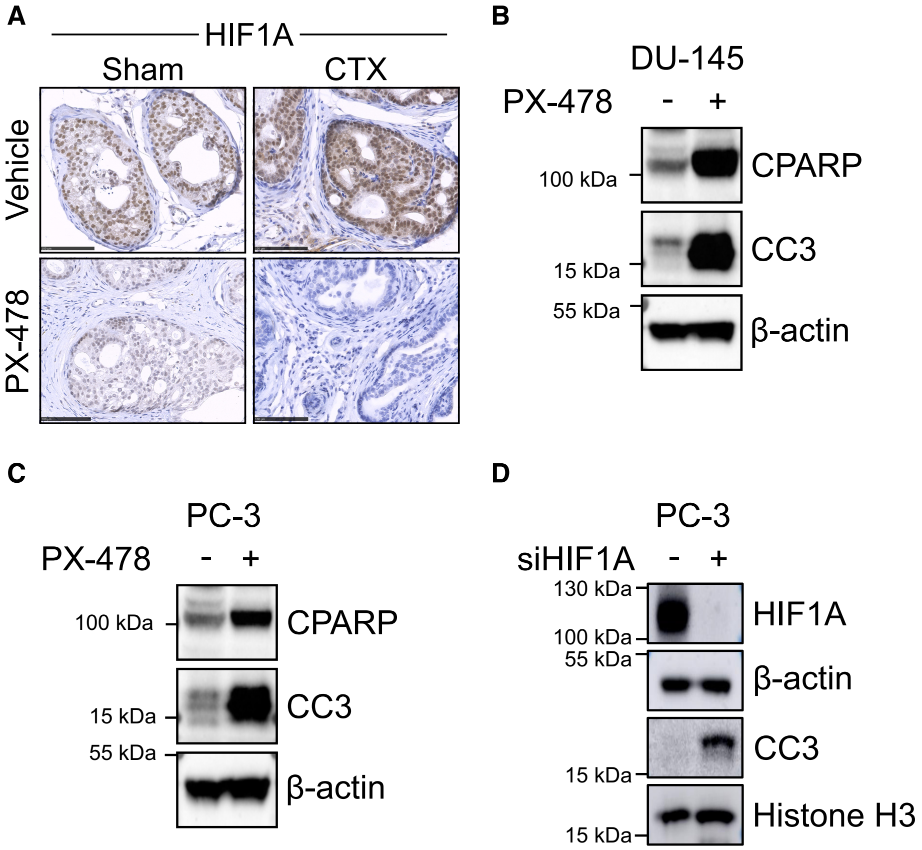

Figure EV3. Effects of HIF1A inhibition on prostate of castrated Pten$^{(i)pe-/-}$ mice and human CRPC cell lines.

A Representative immunohistochemical analysis of HIF1A in DLP of sham and CTX Pten$^{(i)pe-/-}$ mice treated with vehicle or PX-478 as described in Fig 4A, and analyzed at day 25. Scale bar: 100 μm. *N* = 3–4 mice per group.

B, C Western blot analysis of CPARP and CC3 in protein lysates of DU-145 (B) and PC-3 (C) human prostate cell lines treated with vehicle (–) or PX-478 (50 μM) for 24 h. β-actin was used as a loading control. Data are representative of two independent biological replicates.

D Western blot analysis of HIF1A and CC3 in protein lysates of PC-3 cells silenced or not for HIF1A. Histone H3 and β-actin were used as a loading control.

© 2023 The Authors
