# Supplementary material for: Hypoxia‐inducible factor 1A inhibition overcomes castration resistance of prostate tumors
Source: EMBO Mol Med. 2023 Apr 18;15(6):e17209. doi: 10.15252/emmm.202217209 (PMC10245031; doi:10.15252/emmm.202217209)
Supplement: Supplementary file 2 — Table EV1 [file EMMM-15-e17209-s003.docx]

| **Upregulated genes set in CTX versus sham** |  |  |  |  |  |
| --- | --- | --- | --- | --- | --- |
| Gene Set Name | Number of genes in Gene Set (K) | Number of genes in Overlap (k) | k/K | p-value | FDR q-value |
| TNFA SIGNALING VIA NFKB | 196 | 26 | 0.1327 | 9.49E-29 | 4.74E-27 |
| IL2 STAT5 SIGNALING | 199 | 14 | 0.0704 | 3.74E-12 | 9.34E-11 |
| KRAS SIGNALING UP | 198 | 13 | 0.0657 | 5.32E-11 | 8.86E-10 |
| COMPLEMENT | 185 | 12 | 0.0649 | 3.42E-10 | 4.27E-9 |
| APOPTOSIS | 161 | 11 | 0.0683 | 1.09E-9 | 1.09E-8 |
| COAGULATION | 134 | 10 | 0.0746 | 2.69E-9 | 2.24E-8 |
| CHOLESTEROL HOMEOSTASIS | 71 | 8 | 0.1127 | 4.07E-9 | 2.91E-8 |
| ESTROGEN RESPONSE LATE | 194 | 11 | 0.0567 | 7.74E-9 | 4.83E-8 |
| HYPOXIA | 199 | 11 | 0.0553 | 1.01E-8 | 5.6E-8 |
| UV RESPONSE DN | 144 | 9 | 0.0625 | 7.93E-8 | 3.97E-7 |

| **Downregulated genes set in CTX versus sham** |  |  |  |  |  |
| --- | --- | --- | --- | --- | --- |
| Gene Set Name | Number of genes in Gene Set (K) | Number of genes in Overlap (k) | k/K | p-value | FDR q-value |
| INTERFERON ALPHA RESPONSE | 94 | 7 | 0.0745 | 6.16E-8 | 1.78E-6 |
| ANDROGEN RESPONSE | 96 | 7 | 0.0729 | 7.13E-8 | 1.78E-6 |
| ESTROGEN RESPONSE LATE | 194 | 8 | 0.0412 | 6.67E-7 | 9.36E-6 |
| ESTROGEN RESPONSE EARLY | 197 | 8 | 0.0406 | 7.49E-7 | 9.36E-6 |
| INTERFERON GAMMA RESPONSE | 188 | 7 | 0.0372 | 6.64E-6 | 6.64E-5 |
| OXIDATIVE PHOSPHORYLATION | 195 | 7 | 0.0359 | 8.43E-6 | 7.03E-5 |
| P53 PATHWAY | 200 | 7 | 0.0350 | 9.94E-6 | 7.1E-5 |
| ADIPOGENESIS | 200 | 6 | 0.0300 | 1.02E-4 | 6.39E-4 |
| HEME METABOLISM | 188 | 5 | 0.0266 | 6.77E-4 | 3.44E-3 |
| REACTIVE OXIGEN SPECIES PATHWAY | 48 | 3 | 0.0625 | 7.44E-4 | 3.44E-3 |

**Table EV1:** Top 10 hallmarks obtained with Molecular Signature DataBase (MSig DB) of the upregulated (top) and downregulated (bottom) genes in luminal-C cells of castrated Pten^(i)pe-/-^ mice versus sham-operated ones.
