# Supplementary material for: Hypoxia‐inducible factor 1A inhibition overcomes castration resistance of prostate tumors
Source: EMBO Mol Med. 2023 Apr 18;15(6):e17209. doi: 10.15252/emmm.202217209 (PMC10245031; doi:10.15252/emmm.202217209)
Supplement: Supplementary file 4 — PDF+ [file EMMM-15-e17209-s006.pdf]

Article

SOURCE DATA TRANSPARENT PROCESS OPEN ACCESS

EMBO Molecular Medicine

Hypoxia-inducible factor 1A inhibition overcomes castration resistance of prostate tumors

Julie Terzic[1,2,3,4,†], Mohamed A Abu el Maaty[1,2,3,4,5,†], Régis Lutzing[1,2,3,4], Alexandre Vincent[1,2,3,4], Rana El Bizri[1,2,3,4], Matthieu Jung[1,2,3,4], Céline Keime[1,2,3,4] & Daniel Metzger[1,2,3,4,*]

Abstract

Androgen deprivation therapy (ADT) is a cornerstone of prostate cancer (PCa) management. Although tumors initially regress, many progress to a hormone-independent state termed castration-resistant PCa (CRPC), for which treatment options are limited. We here report that the major luminal cell population in tumors of $Pten^{(i)pe-/-}$ mice, generated by luminal epithelial cell-specific deletion of the tumor suppressor PTEN after puberty, is castration-resistant and that the expression of inflammation and stemness markers is enhanced in persistent luminal cells. In addition, hypoxia-inducible factor 1 (HIF1) signaling, which we have previously demonstrated to be induced in luminal cells of $Pten^{(i)pe-/-}$ mice and to promote malignant progression, is further activated. Importantly, we show that genetic and pharmacological inhibition of HIF1A sensitizes Pten-deficient prostatic tumors to castration and provides durable therapeutic responses. Furthermore, HIF1A inhibition induces apoptotic signaling in human CRPC cell lines. Therefore, our data demonstrate that HIF1A in prostatic tumor cells is a critical factor that enables their survival after ADT, and identify it as a target for CRPC management.

Keywords castration-resistant prostate cancer; genetically-engineered mice; HIF1A; PTEN; single-cell RNA sequencing
Subject Categories Cancer; Urogenital System
DOI 10.15252/emmm.202217209 | Received 21 November 2022 | Revised 17 March 2023 | Accepted 20 March 2023 | Published online 18 April 2023
EMBO Mol Med (2023) 15: e17209

Introduction

Prostate cancer (PCa) is the second most commonly diagnosed neoplasia in men worldwide and one of the major causes of cancer-related death (Siegel *et al*, 2017; Rebello *et al*, 2021). Curative options of localized tumors include radical surgical prostatectomy, external beam radiotherapy, or low-dose-rate brachytherapy. However, these treatments often induce side effects, such as impotence, incontinence, and infertility (Resnick *et al*, 2013). The benefit of adding neoadjuvant and adjuvant androgen deprivation therapy (ADT) to achieve castrate levels of testosterone by either surgical orchiectomy or medical castration is well established for men with high-risk localized and advanced disease. Although initially effective, about 30% of patients relapse after first-line treatment (Rebello *et al*, 2021). Moreover, nearly all metastatic patients progress to an androgen-independent state, termed castration-resistant prostate cancer (CRPC) (Parker *et al*, 2020; Rebello *et al*, 2021). Key regulators implicated in the progression of localized disease to CRPC include PTEN, a tumor suppressor gene that is mutated in 12% of localized and metastatic castration-sensitive prostate cancer, and in more than 40% of CRPC patients (Cancer Genome Atlas Research, 2015; Robinson *et al*, 2015). Numerous studies have identified mechanisms of castration resistance, including tumor cell-intrinsic ones, such as androgen receptor (AR) amplifications and mutations, as well as microenvironmental ones, such as the enrichment of immunosuppressive myeloid-derived suppressor cells (MDSCs) (Rebello *et al*, 2021). Despite these findings, the therapeutic arsenal for CRPC is limited (Heidenreich *et al*, 2013). Indeed, a combination of second-generation AR pathway inhibitors (e.g., abiraterone and enzalutamide) and conventional taxane-based chemotherapy (e.g., docetaxel) only improves the survival of CRPC patients by about 5 months (Fizazi *et al*, 2012). Moreover, the inhibition of AR signaling in CRPC by such potent AR inhibitors has been linked to the emergence of lethal neuroendocrine PCa (Davies *et al*, 2018). While the use of PARP inhibitors, such as olaparib and rucaparib, has broadened the therapeutic landscape in the context of metastatic CRPC with alterations in homologous recombination repair genes (ATM, BRCA1, BRCA2), resistance may also emerge (Loehr *et al*, 2022), and the overall survival is improved by around 3 months (Hussain *et al*, 2020). Therefore, elucidating additional targetable mechanisms of castration resistance is highly desired to develop effective therapeutic strategies for CRPC.

1 Institut de Génétique et de Biologie Moléculaire et Cellulaire, Illkirch, France
2 Centre National de la Recherche Scientifique (CNRS), UMR7104, Illkirch, France
3 Institut National de la Santé et de la Recherche Médicale (INSERM), U1258, Illkirch, France
4 Université de Strasbourg, Strasbourg, France
5 Institute for Diabetes and Cancer, Helmholtz Center Munich, and German Center for Diabetes Research (DZD), Neuherberg, Germany
*Corresponding author. Tel: 33 3 88 65 34 63; E-mail: metzger@igbmc.fr
[†]These authors contributed equally to this work

© 2023 The Authors. Published under the terms of the CC BY 4.0 license

We have previously shown that prostatic intraepithelial neoplasia (PIN) of Pten$^{(i)pe-/-}$ mice, generated by luminal epithelial cell-specific deletion of the tumor suppressor PTEN after puberty, is hypoxic and that enhanced hypoxia-inducible factor 1 (HIF1) signaling in luminal cells drives the malignant evolution of such lesions (Abu El Maaty *et al*, 2022). In this study, we analyzed the molecular underpinnings enabling luminal cell survival in tumors of Pten$^{(i)pe-/-}$ mice under androgen deprivation conditions and found that castration resistance is associated with further activation of hypoxic signaling and a shift in luminal cell state. Importantly, we demonstrate that genetic or pharmacological inhibition of hypoxia-inducible factor 1A (HIF1A) overcomes castration resistance and provides a durable therapeutic response. Our results therefore demonstrate that inhibiting HIF1A is a therapeutic strategy for the management of CRPC.

Results

Androgen deprivation further enhances hypoxic signaling in prostatic luminal-C cells

To study the impact of androgen deprivation on Pten-deficient prostatic luminal cells, Pten$^{L2/L2}$ (control) and Pten$^{(i)pe-/-}$ mice were orchiectomized at 3 months after gene invalidation (AGI), and analyzed 1 month after surgery. As expected, the weight of androgen-responsive tissues (e.g., seminal vesicles and bulbocavernosus muscle) was strongly reduced in castrated mice of both genotypes compared with sham-operated ones (Figs EV1A and 1B). Although the prostate weight was reduced by castration in both mouse lines, histological analyses revealed a shrinkage of the dorsolateral prostate (DLP) glands only in castrated Pten$^{L2/L2}$ mice (Figs 1A and EV1C and D). Importantly, a similar proportion of PIN and adenocarcinoma were present in the DLP of sham-operated and castrated Pten$^{(i)pe-/-}$ mice (Fig 1A and B). Of note, immunohistochemical detection of AR revealed a strong nuclear staining in prostates of sham-operated control and mutant mice and cytoplasmic staining in those of castrated ones (Fig 1A). Collectively, these results demonstrate that androgen deprivation impairs AR nuclear translocation but does not induce regression of Pten-deficient prostatic lesions.

To characterize the molecular underpinnings enabling luminal cell survival under androgen deprivation in an unbiased manner, we performed droplet-based single-cell RNA sequencing (scRNA-seq) on 6,166 and 9,940 cells isolated 1 month after surgery from prostates of Pten$^{(i)pe-/-}$ mice, sham-operated and castrated at 3 months AGI, respectively. Analysis of these 16,106 cells identified 21 prostatic clusters (Fig EV1E), which were classified into epithelial cells (*Epcam*; clusters 3, 11, and 17) and mesenchymal cells, the latter comprising leukocytic (*Ptprc*; clusters 1, 5, 6, 7, 9, 10, 12, 14, 15, 16, 18, 20, 21) and nonleukocytic (*Vim*; clusters 2, 4, 8, 13 and 19) cells (Fig EV1E–G). The leukocytic clusters included T- (*Cd3g*) and B-(*Cd79a*) lymphocytes, MDSCs (*S100a8*), dendritic cells (*Flt3*), macrophages (*Adgre1*), and mast cells (*Cpa3* and *Mcpt4*) (Fig 1C). Nonleukocytic mesenchymal cells included endothelial cells (*Pecam1*) and stromal fibroblasts (*Col1a1*). Epithelial clusters comprised basal cells (*Krt5*), as well as two luminal subsets (*Krt8*), termed luminal-A and -C (Fig 1C), characterized by elevated levels of androgen-responsive genes (e.g., *Pbsn*, *Nkx3-1*, and *Tgm4*) (Rennie *et al*, 1993; He *et al*, 1997; Lopez-Bujanda *et al*, 2021), and stemness-associated markers (*Tacstd2* [encoding *Trop2*]/*Krt4*/*Ly6a*), respectively (Fig 1C and D). Luminal-C cells are a minor population in control prostates, and the predominant epithelial subset in Pten-deficient prostatic lesions (Abu El Maaty *et al*, 2021, 2022). Cells with similar characteristics have been recently suggested to be intrinsically castration-resistant (Baures *et al*, 2022). Interestingly, the abundance of luminal-A but not luminal-C cells was reduced after castration (Figs 1D and EV1F and H). These data therefore demonstrate that only a minor population of prostatic luminal cells is eliminated by castration of Pten$^{(i)pe-/-}$ mice, and indicate intrinsic castration-resistant properties for luminal-C cells.

Differential expression analysis revealed that the transcript levels of around 380 genes were altered in luminal-C cells by castration (Dataset EV1). As expected, enrichment analysis of the genes downregulated in luminal-C cells of castrated mice compared with those of sham-operated ones using the Molecular Signatures Database (MSigDB) identified "androgen response" among the top 10 downregulated pathways (Fig 1E; Table EV1), with the expression of a number of androgen-responsive genes, including *Pbsn*, *Tgm4*, and *Fkbp5*, reduced by androgen deprivation (Fig 1F; Dataset EV1). On the other hand, enrichment analysis of the genes upregulated in luminal-C cells of castrated mice identified inflammation-related pathways, including "TNFA signaling via NF-kB" and "IL2-STAT5 signaling" (Fig 1G; Table EV1). A number of genes encoding cytokines, including *Il1b*, *Cxcl1*, and the MDSC-recruiting factor *Cxcl5*, were induced by castration (Fig 1H; Dataset EV1), the latter potentially underscoring the enrichment of MDSCs in prostatic tumors of castrated Pten$^{(i)pe-/-}$ mice (Figs 1I and EV1F).

Interestingly, enrichment analyses also identified "Hypoxia," a major feature of Pten-deficient prostatic lesions, among the hallmarks upregulated in luminal-C cells of castrated mice (Fig 1G; Table EV1). Castration induced the expression of 11 hypoxia-related genes in luminal-C cells (Table 1 and Dataset EV1), including *Pfkfb3* (6-phosphofructo-2-kinase/fructose-2,6-biphosphatase 3), *Cp* (Ceruloplasmin), and *Car12* (Carbonic anhydrase 12) (Fig 1J).

Cd44 and *Ly6d*, which have been previously reported to identify prostate stem cells/progenitors (Barros-Silva *et al*, 2018; Wang *et al*, 2021), were also among the genes upregulated in persistent luminal-C cells (Fig 1K; Dataset EV1), suggesting induced plasticity by castration. We thus determined the levels of Transglutaminase 2 (TGM2), which we have recently shown to be a marker of HIF1A-driven disease progression and luminal plasticity (Abu El Maaty *et al*, 2022). Immunohistochemical analyses revealed that TGM2 was expressed at low levels in less than half of the prostatic epithelial cells of sham-operated Pten$^{(i)pe-/-}$ mice at 3 months AGI and at high levels in most epithelial cells of castrated ones (Fig 1L).

Collectively, these data demonstrate that the abundance of luminal-A is strongly decreased in castrated Pten$^{(i)pe-/-}$ mice, whereas hypoxic signaling is further activated in castration-resistant luminal-C cells, which acquire a high plasticity state and promote an immune-suppressive microenvironment.

Luminal HIF1A enables cellular survival in response to androgen deprivation

Since hypoxic signaling was enhanced in luminal-C cells of castrated Pten$^{(i)pe-/-}$ mice, we determined whether luminal HIF1A is

 © 2023 The Authors

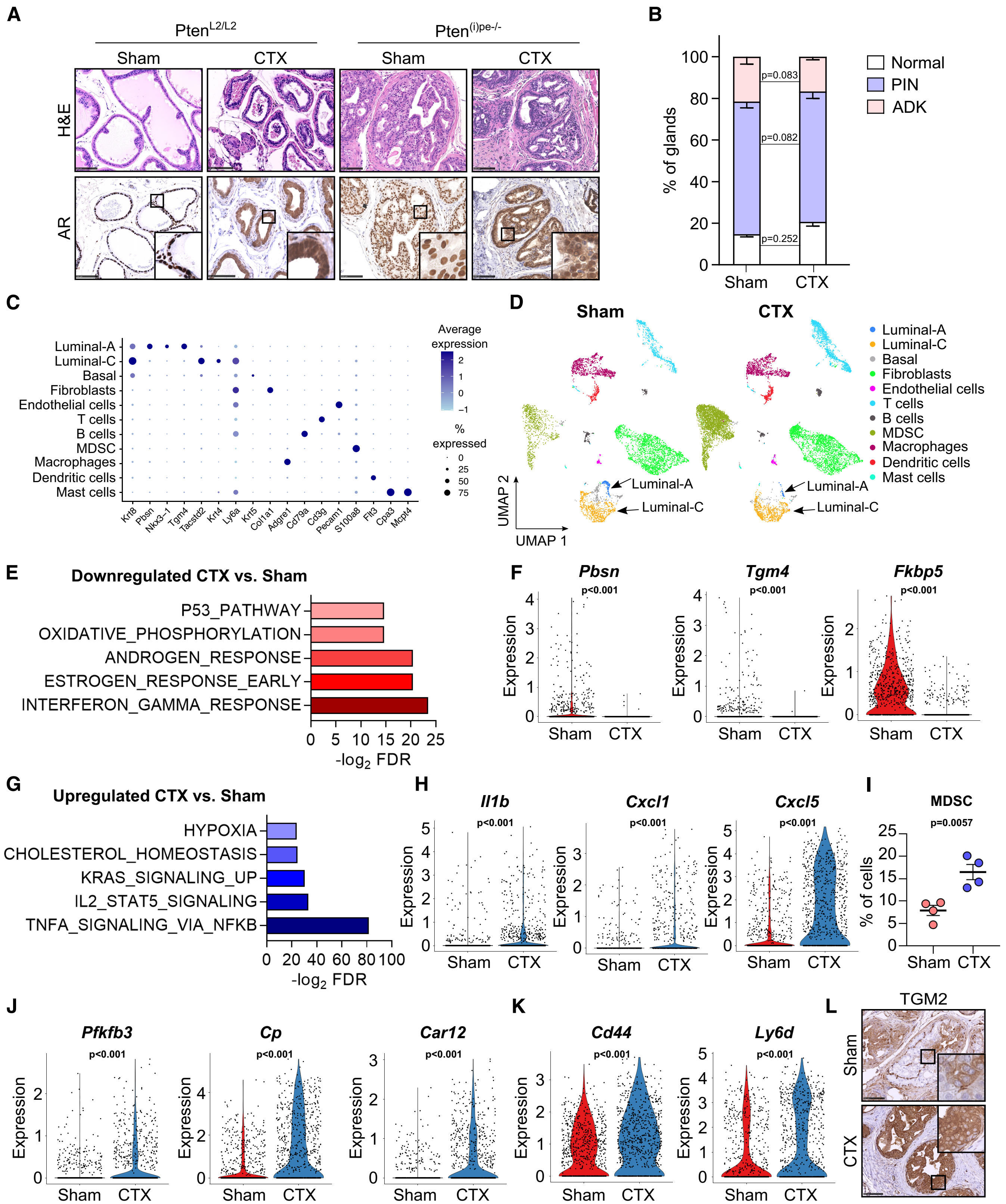
A
Pten L2/L2
Pten (i)pe-/-
Sham
CTX
Sham
CTX
H&E
AR
B
100
80
60
40
20
0
% of glands
p=0.083
p=0.082
p=0.252
Sham
CTX
Normal
PIN
ADK
C
Luminal-A
Luminal-C
Basal
Fibroblasts
Endothelial cells
T cells
B cells
MDSC
Macrophages
Dendritic cells
Mast cells
Krt8
Pbsn
Nkx3-1
Tgm4
Tacstd2
Krt4
Ly6a
Krt5
Col1a1
Adgre1
Cd79a
Cd3g
Pecam1
S100a8
Flt3
Cpa3
Mcpt4
Average expression
% expressed
D
Sham
CTX
Luminal-A
Luminal-C
UMAP 1
UMAP 2
E
Downregulated CTX vs. Sham
P53_PATHWAY
OXIDATIVE_PHOSPHORYLATION
ANDROGEN_RESPONSE
ESTROGEN_RESPONSE_EARLY
INTERFERON_GAMMA_RESPONSE
-log2 FDR
F
Pbsn
Tgm4
Fkbp5
p<0.001
Expression
Sham
CTX
G
Upregulated CTX vs. Sham
HYPOXIA
CHOLESTEROL_HOMEOSTASIS
KRAS_SIGNALING_UP
IL2_STAT5_SIGNALING
TNFA_SIGNALING_VIA_NFKB
-log2 FDR
H
Il1b
Cxcl1
Cxcl5
p<0.001
Expression
Sham
CTX
I
MDSC
p=0.0057
% of cells
Sham
CTX
J
Pfkfb3
Cp
Car12
p<0.001
Expression
Sham
CTX
K
Cd44
Ly6d
p<0.001
L
TGM2
Sham
CTX

Figure 1.

◀ **Figure 1. Characterization of prostate tumors of castrated Pten$^{(i)pe-/-}$ mice.**

A Representative H&E staining and immunohistochemical analysis of the androgen receptor (AR) in the dorsolateral prostate (DLP) of Pten$^{L2/L2}$ and Pten$^{(i)pe-/-}$ mice, sham-operated (sham), or castrated (CTX) at 3 months after gene inactivation (AGI), and analyzed at 1 month after surgery. Scale bars: 100 μm. *N* = 3 mice per condition.

B Histological scoring of DLP of sham and CTX Pten$^{(i)pe-/-}$ mice analyzed at 1 month after surgery. ADK, adenocarcinoma. *N* = 4 mice per condition. Data presented are mean, and error bars correspond to SEM. *P*-values were determined using an unpaired *t*-test.

C Dot plot depicting the expression of cell-lineage specific markers in the various clusters generated after scRNA-seq analysis of prostates of sham and CTX Pten$^{(i)pe-/-}$ mice analyzed at 1 month after surgery.

D Uniform Manifold Approximation and Projection (UMAP) of cells from prostates of sham or CTX Pten$^{(i)pe-/-}$ mice. Luminal-A and Luminal-C cells are indicated by arrows.

E Bar chart depicting the identified hallmarks through Gene Set Enrichment Analysis (GSEA) of the genes downregulated in luminal-C cells of Pten$^{(i)pe-/-}$ mice with castration. FDR, False Discovery Rate.

F Violin plots depicting transcript levels of androgen-responsive genes in luminal-C cells of sham and CTX Pten$^{(i)pe-/-}$ mice. *P*-values were determined by the Wilcoxon rank-sum test.

G Bar chart depicting the identified hallmarks through GSEA of the genes upregulated in luminal-C cells of Pten$^{(i)pe-/-}$ mice with castration.

H Violin plots depicting the transcript levels of inflammation-related genes in luminal-C cells of sham and CTX Pten$^{(i)pe-/-}$ mice. *P*-values were determined by the Wilcoxon rank-sum test.

I Flow cytometric analysis of myeloid-derived suppressor cells (MDSC) in prostates of Pten$^{(i)pe-/-}$ mice, sham and CTX at 3 months AGI and analyzed 1 month later. *N* = 4 mice per group. *P*-values were determined by a two-tailed *t*-test.

J, K Violin plots depicting the transcript levels of hypoxia-related genes (J) and plasticity-related genes (K) in luminal-C cells of sham and CTX Pten$^{(i)pe-/-}$ mice. *P*-values were determined by the Wilcoxon rank-sum test.

L Representative immunohistochemical detection of TGM2 in DLP of sham and CTX Pten$^{(i)pe-/-}$ mice. *N* = 3 mice per condition. Scale bars: 100 μm.

Source data are available online for this figure.

Table 1. List of hypoxia-related genes induced in luminal-C cells of castrated Pten$^{(i)pe-/-}$ mice versus sham-operated ones.

Gene name	*P*-value	Average log2FC	pct.1	pct.2	*P* val_adj
Cp	1.55E-34	2.229	0.595	0.343	3.30E-30
Car12	2.42E-32	0.813	0.409	0.157	5.17E-28
Zfp36	9.50E-15	0.754	0.850	0.807	2.03E-10
Bhlhe40	2.77E-14	0.640	0.668	0.592	5.91E-10
Map3k1	3.07E-13	0.403	0.598	0.518	6.54E-09
Pfkfb3	3.57E-13	0.473	0.346	0.209	7.61E-09
Fos	7.68E-10	0.594	0.904	0.851	1.64E-05
Errfi1	4.09E-09	0.457	0.862	0.824	8.73E-05
Ndrg1	2.07E-08	0.412	0.416	0.306	4.42E-04
Klf6	1.97E-06	0.453	0.924	0.889	4.20E-02
Ccn1	3.82E-04	0.629	0.697	0.679	1.00E+00

Average log2FC: log fold change of the average expression between the two groups. Positive values indicate that the gene is more highly expressed in the first group. Pct.1: percentage of cells where the gene is detected in the first group. Pct.2: percentage of cells where the gene is detected in the second group. *P* val adj: Adjusted *P*-value, based on Bonferroni correction using all genes in the dataset.

involved in resistance to androgen deprivation. To this end, Pten/Hif1a$^{(i)pe-/-}$ mice, in which both *Pten* and *Hif1a* are selectively inactivated in luminal cells after puberty (Abu El Maaty *et al*, 2022), were castrated at 3 months AGI and analyzed 1 month after surgery. The prostate weight of castrated Pten/Hif1a$^{(i)pe-/-}$ mice was markedly lower than that of sham-operated ones, and similar to that of castrated controls (Pten$^{L2/L2}$/Hif1a$^{L2/L2}$ mice; Fig 2A). Histological analyses revealed a striking gland shrinkage in the DLP of castrated Pten/Hif1a$^{(i)pe-/-}$ mice (Fig 2B and C), as observed in castrated controls (Figs 1A and 2C). Moreover, whereas about 75% of glands contained PINs or adenocarcinoma in sham-operated Pten/Hif1a$^{(i)pe-/-}$ mice, > 80% of glands in castrated ones had a neoplastic-free appearance and no adenocarcinoma was observed (Fig 2B and D). Of note, immunohistochemical analyses showed that most of the remaining luminal cells of castrated Pten/Hif1a$^{(i)pe-/-}$ mice expressed the luminal-C marker TROP2 (Abu El Maaty *et al*, 2022) and AR in the cytoplasm (Fig 2E).

To further characterize the impact of HIF1A deficiency on androgen deprivation, we analyzed Pten$^{(i)pe-/-}$ and Pten/Hif1a$^{(i)pe-/-}$ mice at 1 to 3 days after surgery. Although AR was already cytoplasmic 1 day after castration and the weight of the seminal vesicles was reduced after 3 days in both mouse lines (Fig EV2A–C), histological analyses revealed tumor regression in prostates of Pten/Hif1a$^{(i)pe-/-}$ mice but not in those of Pten$^{(i)pe-/-}$ ones (Fig 2F). Furthermore, the proliferation rate of epithelial cells was decreased in both mouse lines 1–3 days after castration (Fig EV2D and E). Note, however, that while cell proliferation further decreased 1 month after castration in Pten/Hif1a$^{(i)pe-/-}$ mice, it increased in Pten$^{(i)pe-/-}$ mice to reach levels similar to those before castration (Fig EV2D and E). Remarkably, castration induced a time-dependent increase in the number of cleaved caspase-3 (CC3)-positive and cleaved poly(ADP-ribose) polymerase (CPARP)-positive epithelial cells within 3 days in prostates of Pten/Hif1a$^{(i)pe-/-}$ mice, but not in those of Pten$^{(i)pe-/-}$ ones (Figs 2G and H, and EV2F–H). Note that < 5% of apoptotic cells were detected in Pten/Hif1a$^{(i)pe-/-}$ mice at 30 days after surgery (Figs 2G and H, and EV2F–H). Thus, androgen deprivation impairs epithelial cell proliferation within 3 days in both Pten$^{(i)pe-/-}$ and Pten/Hif1a$^{(i)pe-/-}$ mice but induces rapid cell death only in the latter. In addition, the antiandrogen enzalutamide induced CC3 levels in organoids generated from prostates of Pten/Hif1a$^{(i)pe-/-}$ mice but not of Pten$^{(i)pe-/-}$ ones (Fig 2I). Collectively, these data demonstrate that luminal HIF1A is a key driver of castration resistance in Pten-deficient prostatic tumors and that its inactivation overcomes this resistance.

© 2023 The Authors

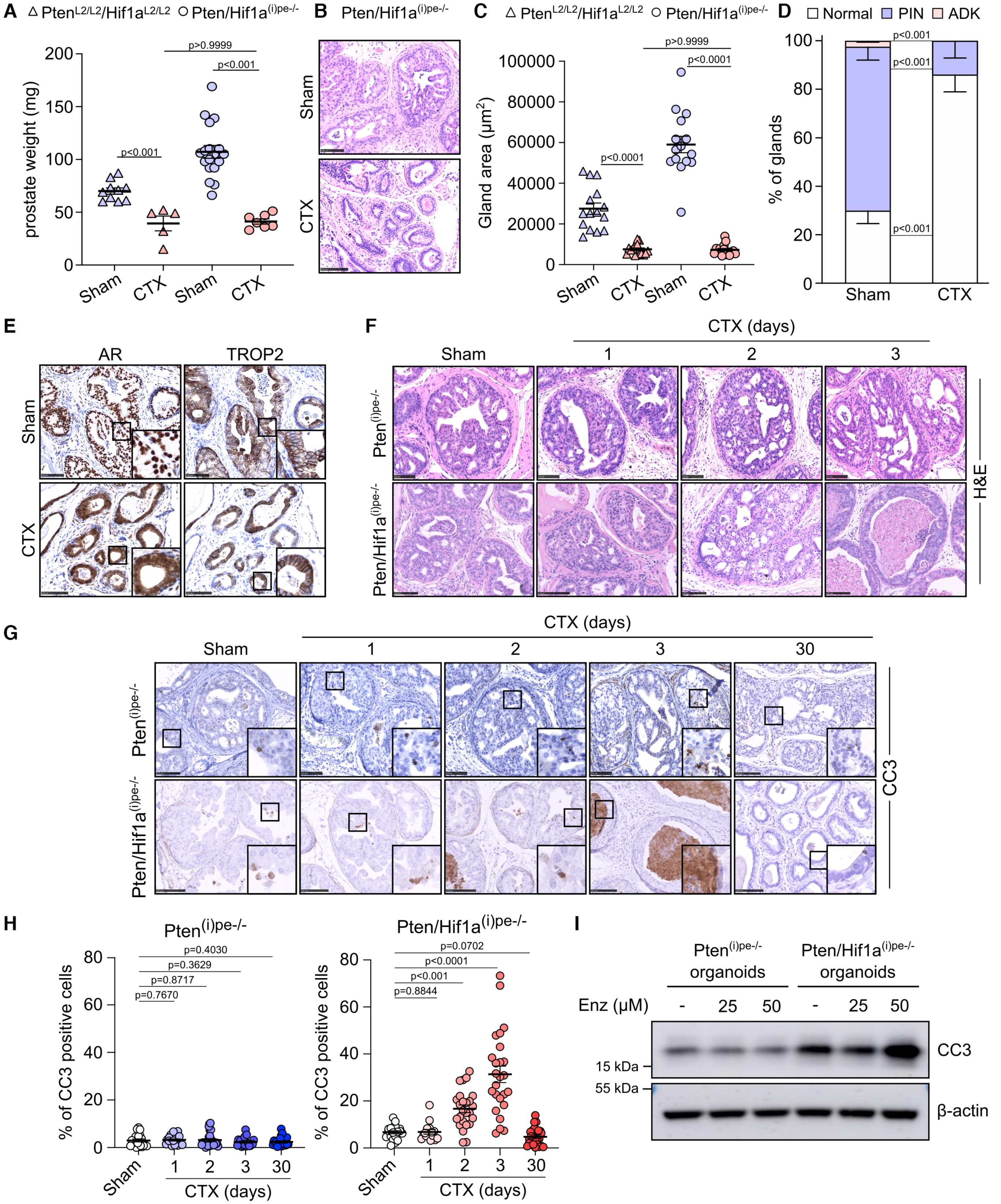
A
Pten^L2/L2/Hif1a^L2/L2
Pten/Hif1a^(i)pe-/-
prostate weight (mg)
p>0.9999
p<0.001
p<0.001
Sham CTX Sham CTX
B
Pten/Hif1a^(i)pe-/-
Sham
CTX
C
Gland area (µm²)
p>0.9999
p<0.0001
p<0.001
D
Normal PIN ADK
% of glands
p<0.001
Sham CTX
E
AR TROP2
Sham
CTX
F
CTX (days)
Sham 1 2 3
Pten^(i)pe-/-
Pten/Hif1a^(i)pe-/-
H&E
G
CTX (days)
Sham 1 2 3 30
Pten^(i)pe-/-
Pten/Hif1a^(i)pe-/-
CC3
H
Pten^(i)pe-/-
% of CC3 positive cells
p=0.4030
p=0.3629
p=0.8717
p=0.7670
CTX (days)
Pten/Hif1a^(i)pe-/-
p=0.0702
p<0.0001
p<0.001
p=0.8844
I
Pten^(i)pe-/- organoids
Pten/Hif1a^(i)pe-/- organoids
Enz (µM) - 25 50 - 25 50
15 kDa
55 kDa
CC3
β-actin

Figure 2.

Figure 2. Effects of castration on prostates of Pten/Hif1a$^{(i)pe-/-}$ mice.

A Prostate weight of control (Pten$^{L2/L2}$/Hif1a$^{L2/L2}$) and Pten/Hif1a$^{(i)pe-/-}$ mice sham or CTX at 3 months AGI and analyzed 1 month after surgery. N = 5–18 mice/condition. Data presented are mean ± SEM. *P*-values were determined by one-way ANOVA followed by a *post hoc* Tuckey test.
B Representative H&E staining of DLP of sham and CTX Pten/Hif1a$^{(i)pe-/-}$ mice. Scale bars: 100 μm. N = 3 mice/condition.
C Gland areas in DLP of sham and CTX control and Pten/Hif1a$^{(i)pe-/-}$ mice. Five glands per mouse were quantified. N = 3 mice/condition. Data presented are mean ± SEM. *P*-values were determined by one-way ANOVA followed by a *post hoc* Tuckey test.
D Histological scoring of DLP of sham and CTX Pten/Hif1a$^{(i)pe-/-}$ mice. N = 3–5 mice/condition. Data presented are mean, and error bars correspond to SEM. *P*-values were determined by an unpaired *t*-test.
E Representative immunohistochemical detection of AR and TROP2 in DLP of sham and CTX Pten/Hif1a$^{(i)pe-/-}$ mice. N = 3–5 mice/condition. Scale bars: 100 μm.
F Representative H&E stained DLP of Pten$^{(i)pe-/-}$ and Pten/Hif1a$^{(i)pe-/-}$ mice sham or CTX at 3 months AGI and analyzed 1 or 1, 2, and 3 days after surgery, respectively. N = 3–5 mice/condition. Scale bars: 100 μm.
G Representative immunohistochemical detection of cleaved caspase 3 (CC3) in DLP of Pten$^{(i)pe-/-}$ and Pten/Hif1a$^{(i)pe-/-}$ mice, sham or CTX at 3 months AGI and analyzed 1 or 1, 2, 3, and 30 days after surgery, respectively. N = 3 mice/condition. Scale bars: 100 μm.
H Quantification of CC3-positive prostatic epithelial cells in DLP of sham-operated and castrated Pten$^{(i)pe-/-}$ and Pten/Hif1a$^{(i)pe-/-}$ mice. N = 3–5 mice/condition. Five glands per mouse were quantified. Data presented are mean ± SEM. *P*-values were determined by one-way ANOVA followed by a *post hoc* Tuckey test.
I Western blot analysis of CC3 in protein lysates of organoids generated from prostates of Pten$^{(i)pe-/-}$ and Pten/Hif1a$^{(i)pe-/-}$ mice at 3 months AGI and treated with enzalutamide (Enz) for 24 h at the indicated concentrations. β-actin was used as a loading control. Data are representative of two independent biological replicates.

Source data are available online for this figure.

Luminal HIF1A inactivation provides a durable therapeutic response to castration

To investigate whether the lack of luminal HIF1A provides long-term therapeutic responses to castration, we analyzed control and Pten/Hif1a$^{(i)pe-/-}$ mice 5 months after surgery. The prostate weight and gland size of castrated Pten/Hif1a$^{(i)pe-/-}$ mice were markedly lower than those of sham-operated ones, and similar to those of castrated controls (Fig 3A–C). Moreover, while around 60 and 20% of glands contained PIN and adenocarcinoma in sham-operated mice, respectively, > 80% of glands had a normal-like appearance in castrated ones and no glands contained adenocarcinoma (Fig 3B and D). The remaining luminal cells after castration expressed AR in the cytoplasm and were TROP2-positive (Fig 3E), in agreement with our results above (Fig 2E). Altogether, our findings demonstrate that HIF1A is a key factor for Pten-deficient luminal cell survival after castration, making HIF1A inhibition a highly attractive approach to overcome castration resistance of prostatic tumors.

Pharmacological HIF1A inhibition sensitizes Pten-deficient tumors to androgen deprivation

Since we demonstrated that HIF1A is a critical mediator of luminal cell response to androgen deprivation, we determined whether pharmacologically inhibiting this factor sensitizes Pten-deficient lesions to castration. We therefore treated Pten$^{(i)pe-/-}$ mice at 3 months AGI for 5 days with PX-478, an orally bioavailable HIF1A inhibitor (Koh *et al*, 2008), after which mice were either castrated or sham-operated, and treated 1 week later with PX-478 for 3 additional weeks (Fig 4A). In agreement with our previous study (Abu El Maaty *et al*, 2022), immunohistochemical analyses revealed that PX-478 treatment strongly reduces HIF1A levels in prostatic epithelial cells of Pten$^{(i)pe-/-}$ mice (Fig EV3A). Histological analyses of the DLP revealed adenocarcinoma in around 20% of glands in vehicle-treated sham-operated and castrated mice, as well as in PX-478-treated sham-operated ones. By contrast, such lesions were found in < 3% of glands in PX-478-treated castrated mice (Fig 4B and C). Moreover, 40% of the glands had a neoplastic-free appearance in PX-478-treated castrated mice compared with around 20% in the 3 other groups. Importantly, PX-478 induced CC3 and CPARP levels in the human C4-2B PCa cells, a castration-resistant subline of the LNCaP-derived C4-2 cells (Spans *et al*, 2014), which were further enhanced when combined with enzalutamide (Fig 4D). Moreover, PX-478 induced CC3 and CPARP levels in the human castration-resistant prostate cancer cell lines DU-145 and PC-3 (Fig EV3B and C), in keeping with previous results showing reduced survival of these cells in presence of PX-478 (Palayoor *et al*, 2008). Furthermore, HIF1A silencing in PC-3 cells enhanced CC3 levels (Fig EV3D), demonstrating that pharmacological inhibition of HIF1A induces profound anticancer effects in castration-resistant prostate tumor cells.

To determine whether HIF1A inhibition also impacts tumors after androgen deprivation-induced cell plasticity, Pten$^{(i)pe-/-}$ mice were treated with PX-478 1 month after castration (Fig 4E). While only 5 and 25% of the DLP glands had a normal architecture in vehicle-treated castrated and PX-478-treated sham-operated Pten$^{(i)pe-/-}$ mice, respectively, > 50% of the glands were tumor-free in PX-478-treated castrated Pten$^{(i)pe-/-}$ mice (Fig 4F and G). Moreover, PX-478 treatment of castrated mice markedly reduced the DLP gland area (Fig 4H). Therefore, these data demonstrate that inhibiting HIF1A overcomes resistance to castration even after androgen deprivation-induced luminal cell plasticity.

Discussion

Androgen deprivation therapy is a cornerstone in the management of men with high-risk localized and advanced PCa. However, despite initial efficacy, many tumors progress to CRPC for which treatment options remain limited. Therefore, elucidating new targets for CRPC management is in demand. By analyzing castration-resistant Pten-deficient prostatic lesions, we discovered that androgen deprivation further enhances hypoxic signaling in luminal cells and induces a high plasticity state in them. Furthermore, we found that it triggers inflammation-related pathways, increases the expression of cytokines in such lesions, and enhances the prostatic infiltration of MDSCs, thus promoting an immunosuppressive environment. Importantly, we demonstrate that HIF1A is a critical factor enabling tumor cell survival in response to ADT and that genetic or pharmacological inhibition of HIF1A sensitizes Pten-deficient lesions to castration, and leads to durable therapeutic responses.

 © 2023 The Authors

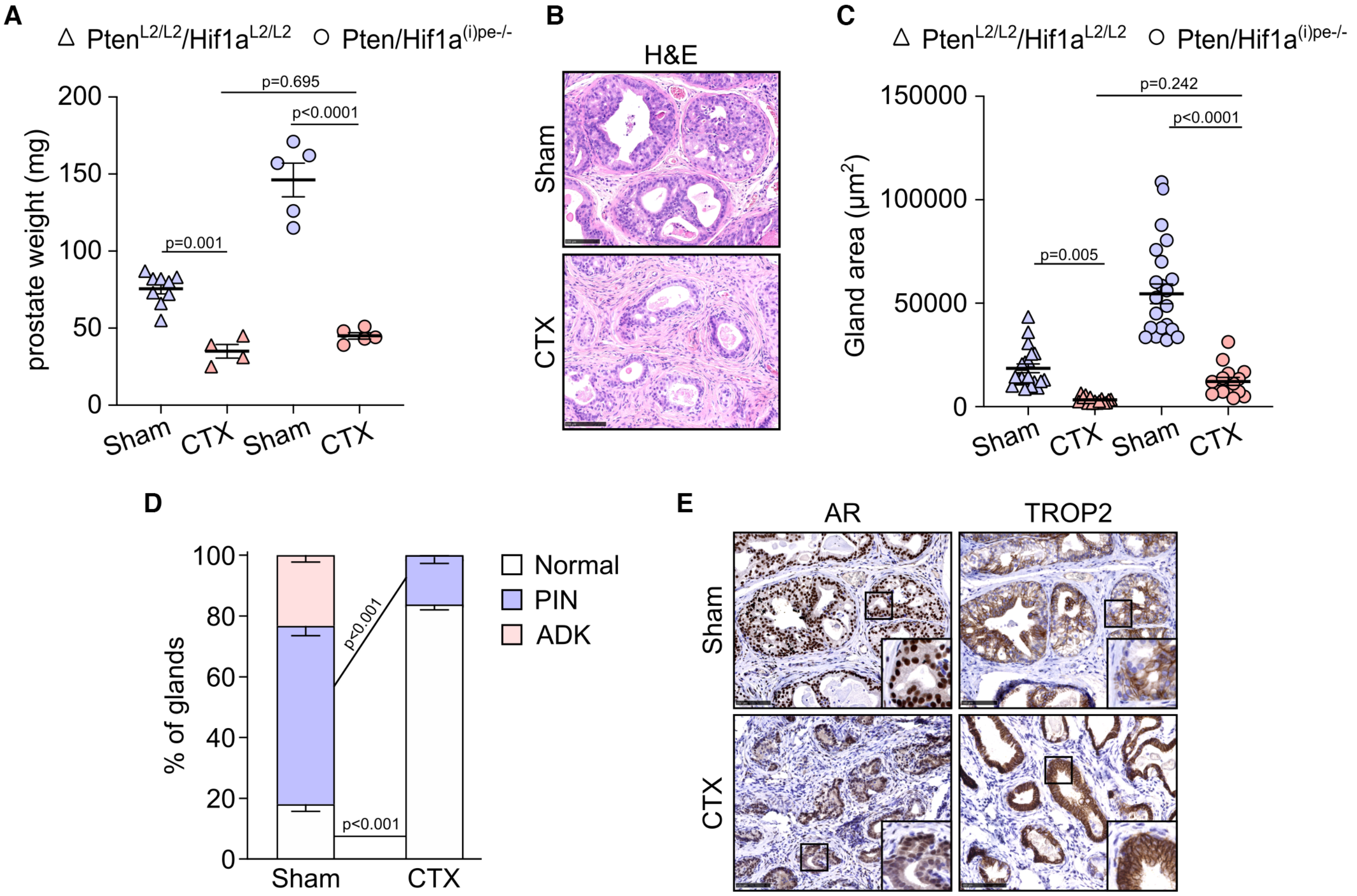

Figure 3. Long-term effects of castration on prostates of Pten/Hif1a$^{(i)pe-/-}$ mice.

A Prostate weight of control (Pten/Hif1a$^{L2/L2}$) and Pten/Hif1a$^{(i)pe-/-}$ mice, sham or CTX at 3 months AGI and determined at 5 months after surgery. N = 4–9 mice/condition. Data presented are mean ± SEM. P-values were determined by one-way ANOVA followed by a *post hoc* Tuckey test.

B Representative H&E staining of DLP of Pten/Hif1a$^{(i)pe-/-}$ mice, sham and CTX at 3 months AGI and analyzed at 5 months after surgery. N = 4–5 mice/condition. Scale bars: 100 μm.

C Gland areas in DLP of control and Pten/Hif1a$^{(i)pe-/-}$ mice, sham and CTX at 3 months AGI and analyzed at 5 months after surgery. N = 3–4 mice/condition. Five glands per mouse were quantified. Data presented are mean, and error bars correspond to SEM. P-values were determined by one-way ANOVA followed by a *post hoc* Tuckey test.

D Histological scoring of DLP of Pten/Hif1a$^{(i)pe-/-}$ mice, sham and CTX at 3 months AGI and analyzed at 5 months after surgery. Data presented are mean, and error bars correspond to SEM. N = 3–5 mice/condition. P-values were determined by an unpaired t-test.

E Representative immunohistochemical detection of AR and TROP2 in DLP of Pten/Hif1a$^{(i)pe-/-}$ mice, sham and CTX at 3 months AGI and analyzed at 5 months after surgery. N = 3–4 mice/condition. Scale bars: 100 μm.

Source data are available online for this figure.

Furthermore, our study demonstrates that inhibiting HIF1A relieves castration resistance even after castration-induced plasticity of luminal cells.

Prostatic tumors of mouse models based on the prostate-specific deletion of Pten have been previously shown to be castration-resistant (Wang *et al*, 2003; Mulholland *et al*, 2011). Our scRNA-seq analysis revealed that distinct luminal subsets in the prostatic epithelium of Pten$^{(i)pe-/-}$ mice exhibit different sensitivities to androgen deprivation, where most luminal-A cells are eliminated by castration, whereas luminal-C cells are castration-resistant, which is in line with the identification of *Tacstd2* (*TROP2*)-expressing luminal cells as intrinsically castration-resistant (Baures *et al*, 2022). Importantly, our results demonstrate that in addition to promoting the plasticity and malignant progression of luminal-C cells (Abu El Maaty *et al*, 2022), HIF1A signaling underscores their castration-resistant phenotype. The persistent luminal-C cells are characterized by the enrichment of stemness- and inflammation-related pathways. Recent studies have reported that JAK/STAT inflammatory signaling drives lineage plasticity in PCa, and that inhibiting JAK and FGFR sensitizes castration-resistant tumors to ADT (Chan *et al*, 2022; Deng *et al*, 2022). In view of the established crosstalk between HIF1A and inflammatory transcription factors including STAT3 and NF-kB (Palazon *et al*, 2014), it is conceivable that hypoxia- and inflammation-driven therapeutic resistance pathways are linked.

Previous studies have demonstrated the impact of microenvironmental cells, including MDSCs (Calcinotto *et al*, 2018), macrophages

© 2023 The Authors

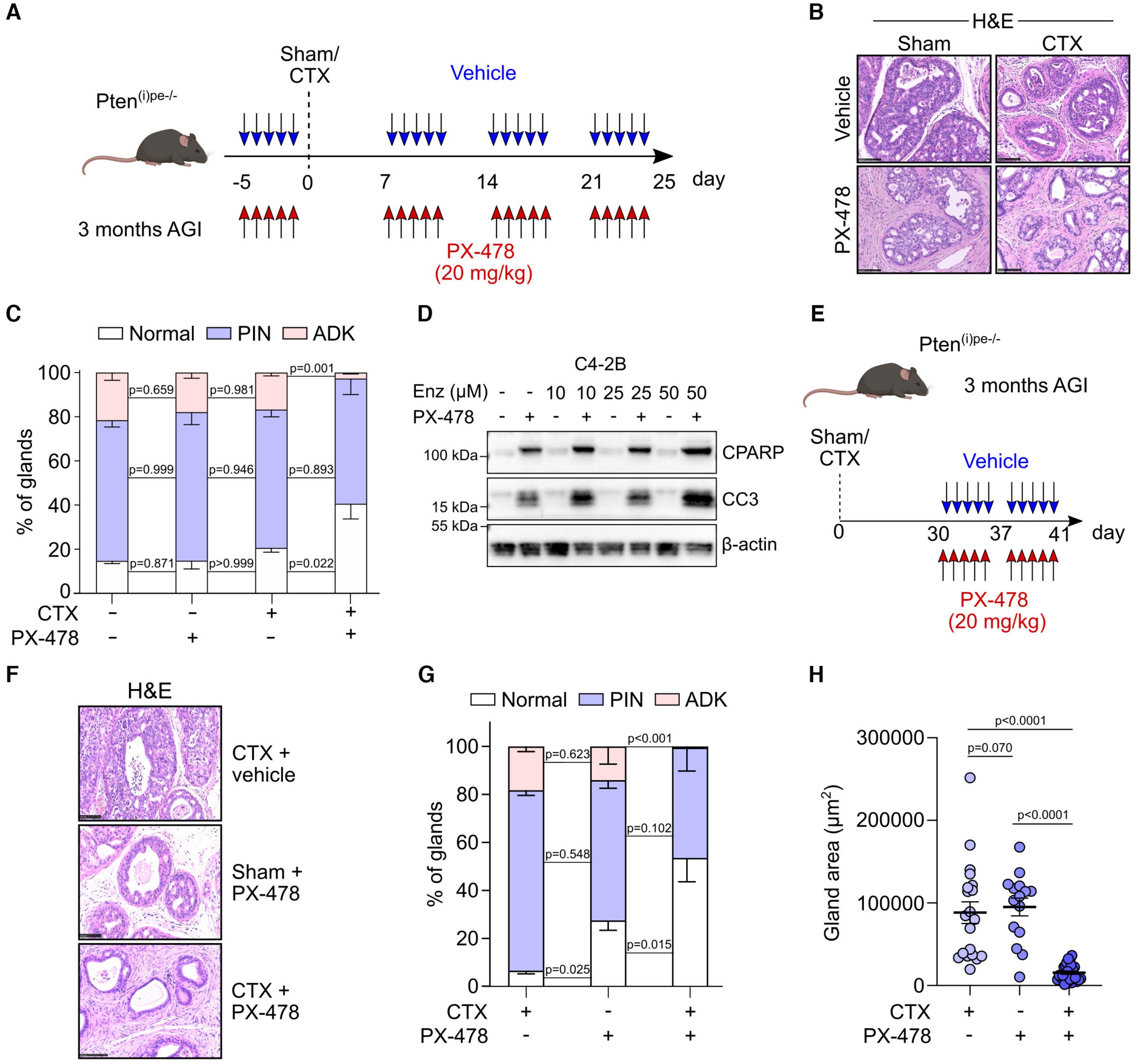

Figure 4. Effects of pharmacological HIF1A inhibition on prostates of castrated Pten$^{(i)pe-/-}$ mice.

A Schematic representation of the experimental protocol investigating the effects of combining HIF1A inhibition with androgen deprivation.

B Representative H&E staining of DLP of sham and CTX Pten$^{(i)pe-/-}$ mice, treated with PX-478 or vehicle, as depicted in (A). Scale bars: 100 μm. *N* = 4–6 mice/condition.

C Histological scoring of DLP of sham-operated and CTX Pten$^{(i)pe-/-}$ mice, treated with PX-478 or vehicle, as depicted in (A). *N* = 4–6 mice/condition. Data presented are mean, and error bars correspond to SEM. *P*-values were determined by an unpaired *t*-test.

D Representative western blot analysis of CPARP and CC3 in human C4-2B PCa cells treated for 24 h with PX-478, increasing concentrations of enzalutamide (Enz), or a combination of both. Beta-actin was used as a loading control.

E Schematic representation of the experimental protocol investigating the potential of PX-478 to reverse castration resistance in Pten$^{(i)pe-/-}$ mice.

F Representative H&E staining of DLP of sham and CTX Pten$^{(i)pe-/-}$ mice, treated with vehicle or PX-478, as depicted in (E). Scale bar: 100 μm. *N* = 3–4 mice/condition.

G Histological scoring of glands in DLP of sham and CTX Pten$^{(i)pe-/-}$ mice, treated with vehicle or PX-478, as depicted in (E). Data presented are mean, and error bars correspond to SEM. *N* = 3–5 mice/condition. *P*-values were determined by an unpaired *t*-test.

H Gland areas of DLP of sham and CTX Pten$^{(i)pe-/-}$ mice, treated with vehicle or PX-478, as depicted in (E). *N* = 3–4 mice/condition. Data presented are mean ± SEM. Five glands per mouse were quantified, and *P*-values were determined by one-way ANOVA followed by a *post hoc* Tuckey test.

Source data are available online for this figure.

© 2023 The Authors

(El-Kenawi *et al*, 2021), and cancer-associated fibroblasts (Zhang *et al*, 2020), on the development of CRPC and sensitivity to antiandrogens. As luminal HIF1A promotes MDSC recruitment to prostatic lesions (Abu El Maaty *et al*, 2022), the reduced prostatic infiltration of MDSCs by HIF1A inhibition might contribute to the enhanced sensitivity of Pten-deficient luminal cells to androgen deprivation. Our findings support the results of Calcinotto *et al* (2018), who showed that treating prostate cancer mouse models with a CXCR2 antagonist decreases the number of MDSCs and prevents the progression to CRPC. However, our data demonstrating apoptotic activation by enzalutamide in organoids generated from prostates of Pten/Hif1a$^{(i)pe-/-}$ mice but not in those generated from Pten$^{(i)pe-/-}$ ones show that cell-autonomous mechanisms play an important role in the enhanced sensitivity to AR signaling blockade by HIF1A inhibition. Importantly, HIF1A inhibition also triggered apoptotic signaling in human CRPC cell line C4-2B that was enhanced by enzalutamide, and in the PTEN-expressing and PTEN-null human CRPC cell lines DU-145 and PC-3, respectively, that express high levels of CD44 and TROP2 (Tang *et al*, 2022). Thus, these results are in line with our *in vivo* data showing that PX-478 sensitizes to castration luminal-C cells that also express such stem cell markers.

In conclusion, our study demonstrates that HIF1A inhibition overcomes castration resistance in Pten-deficient prostatic tumors in mice and induces apoptotic signaling in human castration-resistant cell lines. These compelling findings therefore highlight the importance of analyzing tumoral HIF1 signaling in patients whose disease progresses after ADT and pave the way for the clinical evaluation of HIF1A inhibitors in men with CRPC.

Materials and Methods

Mice

Pten$^{L2/L2}$, Pten$^{(i)pe-/-}$, Pten/Hif1a$^{L2/L2}$, and Pten/Hif1a$^{(i)pe-/-}$ male mice (all on a C57BL/6 genetic background) were generated as described (Ratnacaram *et al*, 2008; Abu El Maaty *et al*, 2022). Mice were maintained in a temperature- and humidity-controlled animal facility, with a 12-h light/dark cycle. Standard rodent chow (2,800 kcal/kg, Usine d'Alimentation Rationelle, Villemoisson-sur-Orge, France) and water were provided *ad libitum*. Mice breeding and maintenance were done in the accredited IGBMC/ICS animal house (C67-2018-37), in compliance with French and EU regulations on the use of laboratory animals for research. Animal experiments were approved by the Ethical committee Com'Eth (Comité d'Ethique pour l'Expérimentation Animale, Strasbourg, France) and the French ministry of Higher Education and Research (#34298-2021121009278850 v5).

Tamoxifen was administered at 2 months of age, as described (Ratnacaram *et al*, 2008). Bilateral orchiectomy was performed under aseptic conditions by trained operators. Control animals underwent an abdominal laparotomy without resection of testicles (sham operation). Surgery was performed 3 months AGI under anesthesia with isoflurane (4%). Mice were intraperitoneally treated with buprenorphine (0.1 mg/kg) before surgery and with meloxicam (2 mg/kg) at the end of the surgery to prevent pain. Mice were monitored for 3 days after surgery, and buprenorphine was administered once a day to those that developed pain signs.

PX-478 2HCl (Selleck Chemicals; catalog Nr S7612) was dissolved in water and administered to mice at a daily dose of 20 mg/kg via oral gavage (Abu El Maaty *et al*, 2022). Mice were randomized and analyzed by investigators not blinded to the genotype and treatment. A sample size calculation was not performed. No outliers were eliminated. Mice were euthanized by cervical dislocation and tissues were harvested.

Organoid and cell cultures

Prostate organoid cultures were established from Pten$^{(i)pe-/-}$ and Pten/Hif1a$^{(i)pe-/-}$ mice at 3 months AGI and cultured as described (Karthaus *et al*, 2014; Drost *et al*, 2016; Abu El Maaty *et al*, 2022) and treated for 24 h with enzalutamide (MDV3100; Selleckchem; catalog No.S1250) at 25 and 50 μM, or vehicle (DMSO). C4-2B (CRL-3315), DU-145 (HTB-81), and PC-3 (CRL-1435) cells were obtained from ATCC and cultured in DMEM (4.5 g/l glucose, 10% FCS, 1% penicillin/streptomycin) at 37°C and 5% CO_2. Cell lines used were mycoplasma-free but not recently authenticated. HIF1A silencing in PC-3 cells was performed as described (Abu El Maaty *et al*, 2022).

C4-2B cells were treated for 24 h with PX-478 (Selleck Chemicals; catalog No.S7612) at 50 μM and/or with enzalutamide (MDV3100; Selleckchem; catalog No.S1250) at 10, 25 and 50 μM, or vehicle (DMSO). DU-145 and PC-3 cells were treated for 24 h with PX-478 at 50 μM.

Histological examination

Five micrometre sections were prepared from paraffin-embedded prostates and dried overnight at 37°C. Hematoxylin and eosin (H&E) staining was performed on prostate sections according to standard protocols. Slides were scanned in brightfield mode using the NanoZoomer digital slide scanner (Hamamatsu) and analyzed using the NDP.view2 Viewing software (Hamamatsu). Representative images of the DLP are provided. Histopathological assessment was performed on the DLP. The number of glands with a normal architecture, PINs, and adenocarcinomas was determined by a resident in oncology, as described (Shappell *et al*, 2004). The area of at least five representative glands of the DLP was determined in each sample using the Qu-Path software (Bankhead *et al*, 2017).

Immunostaining

Five micrometre prostate sections were deparaffinized according to standard protocols and incubated for 20 min in SignalStain® Citrate Unmasking Solution (10×) (CST 14746) in a pressure cooker for heat-induced antigen retrieval. The following primary antibodies were used: TROP2 (Abcam ab214488; dilution 1:200), Androgen receptor (Abcam ab108341; dilution 1:200), TGM2 (CST, 3557; dilution 1:100), HIF1A (Abcam, ab51608; dilution 1:100), Ki-67 (Thermo Fisher Scientific, MA5-14520; dilution 1:200), cleaved caspase 3 (CST 9664; dilution 1:200), and cleaved PARP (Asp124) D6X6X (CST, 94885; dilution 1:50). One drop of SignalStain® Boost IHC Detection Reagent (CST 8114) was added to each section and SignalStain® DAB Substrate Kit (CST 8059) was used to develop the signal, according to the manufacturer's instructions. The sections were counterstained with hematoxylin and mounted.

© 2023 The Authors

The number of Ki-67-positive, CC3-positive, and CPARP-positive cells was determined using the Qu-path software (Bankhead *et al*, 2017).

Western blotting

Prostatic organoids and PCa cell lines were lysed in RIPA buffer, supplemented with protease (05892970001; Sigma-Aldrich) and phosphatase PhoSTOP (PHO SS-RO; Sigma-Aldrich) inhibitors. Protein concentrations were determined using Bradford assay (Abcam; ab119216). Equal amounts of proteins were resolved on SDS–PAGE, and transferred onto nitrocellulose membranes using Trans-blot turbo transfer system (Bio-Rad).

Membranes were incubated with anti-CC3 (CST 9664), anti-cleaved PARP (CST 9544), anti-HIF1A (CST, 36169), anti-histone H3 (CST, 4499S), and anti-ß-actin (SCBT SC-47778 or Sigma-Aldrich A5441) primary antibodies diluted at 1:1,000. Membranes were then incubated with anti-Mouse IgG (CST 7076S) or anti-Rabbit IgG (CST 7074S) HRP-linked antibodies diluted at 1:5,000. Signals were developed using Lightning Plus-ECL Enhanced Chemiluminescence Substrate (Perkin Elmer; ref: NEL104001EA) and detected using an Amersham™ Imager 600 (GE Healthcare).

MDSC quantification

Mice prostates were dissociated into single cells as described (Abu El Maaty *et al*, 2022). Cells were incubated on ice for 15 min with anti-CD16/32 antibody (BD Pharmingen; 1:50). To quantify MDSCs ($CD45^+Gr\text{-}1^+CD11b^+$), cell suspensions were incubated with anti-CD45 (BioLegend; ref. 103128; conjugation: Alexa Fluor 700), anti-Ly6G/Ly-6C (Gr-1) (eBioscience; ref. 11-5931-82; conjugation: fluorescein isothiocyanate [FITC]), and anti-CD11b (eBioscience; ref. 45-0112-82; conjugation: PerCP-Cy5.5) antibodies for 15 min on ice. All antibodies were diluted 1:200 in DMEM (glucose [4.5 g/l], 1% penicillin/streptomycin, and 2% bovine serum albumin, without phenol red). Cells were analyzed using a BD LSR II flow cytometer and the FlowJo software.

Living cell FACS-sorting for scRNA-seq analysis

Mice prostates were dissociated into single cells as described (Abu El Maaty *et al*, 2022). Cells from three prostates for each condition were pooled and stained with DAPI, and living cells (DAPI-negative) were FACS-sorted using a BD FACSAria™ Fusion flow cytometer.

scRNA-seq and data analysis

Cell number and viability of FACS-sorted cells were performed with trypan blue exclusion assay, as described (Abu El Maaty *et al*, 2022). Cells with a viability of > 95% were processed using the Chromium Controller (10× Genomics, Leiden, The Netherlands). Ten thousand cells were loaded per well to capture between 5,000 and 10,000 cells in nanoliter-scale Gel Beads-in-Emulsion (GEMs). Libraries were generated and sequenced on Illumina NextSeq 550 as 100 bases paired-end reads, as described (Abu El Maaty *et al*, 2022). RA 2.7.7 and Cell Ranger 3.0.2 mkfastq were used for image analysis, base calling, and demultiplexing. Cell Ranger 6.0.2 count and the mouse reference 2020-A (GRCm38 Ensembl release 98 and Gencode release M23) were used for alignment, barcode, and UMI filtering and counting.

Seurat R package (Butler *et al*, 2018) version 4.0.5 was used to perform the single-cell analysis. Briefly, for each sample, cells expressing more than 8,000 genes, < 200 genes, and with a percentage of mitochondrial reads > 30% were discarded from the analysis. Sample counts were then normalized using LogNormalize method before being integrated together with anchors found using the 20 first dimensions. Cells expressing Pate4 (normalized count > 4) were discarded from the analysis, as they have been previously reported to be epididymal contaminants (Abu El Maaty *et al*, 2021, 2022). The resulting integrated object was then scaled, and a UMAP was constructed using the 20 first principal components of a PCA analysis. Cell clustering was performed using FindNeighbors and FindClusters (with a resolution of 0.5). Differentially expressed genes between samples were identified using a Wilcoxon test whose performance for single-cell differential expression analysis has been evaluated (Mou *et al*, 2019). Pathway analysis was performed using the MSigDB (Subramanian *et al*, 2005; Liberzon *et al*, 2015).

Statistical analysis

Error bars represent the standard error of the mean. Comparison between two groups was performed using a two-tailed Student's *t*-test. One-way ANOVA followed by a Tuckey test was performed to compare more than two groups. *P*-values < 0.05 were considered significant. Specific *P*-values are indicated in the figures. The number of replicates is indicated in the figure legends. DEGs were defined as having a $\log_2$ fold change ≥ 0.4 and a *P*-value < 0.05, calculated using the Wilcoxon rank-sum test.

The paper explained

Problem

Hormonal therapy known as androgen deprivation therapy is used to treat men with prostate cancer. While tumors initially respond to this treatment, disease relapse occurs in a subset of patients, and tumors progress to an advanced stage known as castration-resistant prostate cancer (CRPC), for which treatment options remain limited. In this study, we sought to identify novel therapeutic targets for the management of men with CRPC.

Results

We analyzed prostates of castration-resistant genetically-engineered mice using single-cell transcriptomics and unraveled that hypoxia-inducible factor 1 (HIF1) signaling is induced in persistent Pten-deficient prostatic luminal cells. We found that genetic and pharmacological inhibition of HIF1A in prostatic lesions sensitizes them to androgen deprivation, and leads to durable therapeutic responses. In addition, we demonstrate that HIF1A inhibition induces apoptotic signaling in human CRPC cell lines.

Impact

Our study demonstrates that HIF1A inhibition is an efficient strategy to target castration-resistant prostatic tumors, and paves the way for the clinical evaluation of HIF1A inhibitors in the management of CRPC.

© 2023 The Authors

Data availability

scRNA-seq data used in this study are deposited in the Gene Expression Omnibus repository (accession numbers: GSE216158). https://www.ncbi.nlm.nih.gov/geo/query/acc.cgi?acc=GSE216158.

Expanded View for this article is available online.

Acknowledgements
We thank the IGBMC mouse, cell culture and imaging facilities, the flow cytometry facility members C. Ebel and M. Phillips, the ICS histopathology facility and GenomEast, and a member of the “France Génomique” consortium (ANR-10-INBS-0009) for excellent assistance. We are grateful to G. Laverny for helpful discussions. This study was supported by funds from the Centre National de la Recherche Scientifique (CNRS) (D.M.), Institut National de la Santé et de la Recherche Médicale (INSERM) (D.M.), Université de Strasbourg (D.M.), Interdisciplinary Thematic Institute IMCBio, as part of the ITI 2021-2028 program of the University of Strasbourg, CNRS and Inserm, supported by IdEx Unistra (ANR-10-IDEX-0002), and by SFRI-STRAT’US project (ANR 20-SFRI-0012) and EUR IMCBio (ANR-17-EURE-0023) under the framework of the French Investments for the Future Program (D.M.), Fondation pour la Recherche Médicale grant EQU201903007800 (D.M.), ITMO Cancer within the funds of the Cancer Plan 2014–2019 (administrated by INSERM) and with a collaborative support from the French National Cancer Institute (D.M.), INCa-PLBIO 2021-074 (D.M.), Fondation ARC pour la Recherche sur le Cancer (PGA-2022-www.fondationARC.org) (D.M.), Agence Nationale de la Recherche grant ANR-10-LABX-0030-INRT under the frame program Investissements d’Avenir labeled ANR-10-IDEX-0002-02 (D.M.), Fondation ARC pour la Recherche sur le Cancer (M.A.A.e.M.), ITMO Cancer Aviesan/Inserm/Cancer 2020 through the “Formation à la recherche fondamentale et translationnelle en cancérologie” program (J.T.), Al Bizri Foundation (R.E.B.). A CC-BY public copyright license has been applied by the authors to the present document and will be applied to all subsequent versions up to the Author Accepted Manuscript arising from this submission, in accordance with the grant’s open access conditions.

Author contributions
Julie Terzic: Conceptualization; formal analysis; investigation; visualization; methodology; writing – original draft; writing – review and editing. **Mohamed A Abu el Maaty:** Conceptualization; formal analysis; investigation; visualization; methodology; writing – original draft; writing – review and editing. **Régis Lutzing:** Investigation. **Alexandre Vincent:** Investigation. **Rana El Bizri:** Investigation. **Matthieu Jung:** Validation; investigation; methodology. **Céline Keime:** Investigation; visualization; methodology. **Daniel Metzger:** Conceptualization; supervision; funding acquisition; writing – original draft; project administration; writing – review and editing.

Disclosure and competing interests statement
The authors declare that they have no conflict of interest.

References

Abu El Maaty MA, Grelet E, Keime C, Rerra AI, Gantzer J, Emprou C, Terzic J, Lutzing R, Bornert JM, Laverny G *et al* (2021) Single-cell analyses unravel cell type-specific responses to a vitamin D analog in prostatic precancerous lesions. *Sci Adv* 7: eabg5982

Abu El Maaty MA, Terzic J, Keime C, Rovito D, Lutzing R, Yanushko D, Parisotto M, Grelet E, Namer IJ, Lindner V *et al* (2022) Hypoxia-mediated stabilization of HIF1A in prostatic intraepithelial neoplasia promotes cell plasticity and malignant progression. *Sci Adv* 8: eabo2295

Bankhead P, Loughrey MB, Fernandez JA, Dombrowski Y, McArt DG, Dunne PD, McQuaid S, Gray RT, Murray LJ, Coleman HG *et al* (2017) QuPath: open source software for digital pathology image analysis. *Sci Rep* 7: 16878

Barros-Silva JD, Linn DE, Steiner I, Guo G, Ali A, Pakula H, Ashton G, Peset I, Brown M, Clarke NW *et al* (2018) Single-cell analysis identifies LY6D as a marker linking castration-resistant prostate luminal cells to prostate progenitors and cancer. *Cell Rep* 25: 3504 – 3518.e6

Baures M, Dariane C, Tika E, Puig Lombardi E, Barry Delongchamps N, Blanpain C, Guidotti JE, Goffin V (2022) Prostate luminal progenitor cells: from mouse to human, from health to disease. *Nat Rev Urol* 19: 201 – 218

Butler A, Hoffman P, Smibert P, Papalexi E, Satija R (2018) Integrating single-cell transcriptomic data across different conditions, technologies, and species. *Nat Biotechnol* 36: 411 – 420

Calcinotto A, Spataro C, Zagato E, Di Mitri D, Gil V, Crespo M, De Bernardis G, Losa M, Mirenda M, Pasquini E *et al* (2018) IL-23 secreted by myeloid cells drives castration-resistant prostate cancer. *Nature* 559: 363 – 369

Cancer Genome Atlas Research N (2015) The molecular taxonomy of primary prostate cancer. *Cell* 163: 1011 – 1025

Chan JM, Zaidi S, Love JR, Zhao JL, Setty M, Wadosky KM, Gopalan A, Choo ZN, Persad S, Choi J *et al* (2022) Lineage plasticity in prostate cancer depends on JAK/STAT inflammatory signaling. *Science* 377: 1180 – 1191

Davies AH, Beltran H, Zoubeidi A (2018) Cellular plasticity and the neuroendocrine phenotype in prostate cancer. *Nat Rev Urol* 15: 271 – 286

Deng S, Wang C, Wang Y, Xu Y, Li X, Johnson NA, Mukherji A, Lo UG, Xu L, Gonzalez J *et al* (2022) Ectopic JAK-STAT activation enables the transition to a stem-like and multilineage state conferring AR-targeted therapy resistance. *Nat Cancer* 3: 1071 – 1087

Drost J, Karthaus WR, Gao D, Driehuis E, Sawyers CL, Chen Y, Clevers H (2016) Organoid culture systems for prostate epithelial and cancer tissue. *Nat Protoc* 11: 347 – 358

El-Kenawi A, Dominguez-Viqueira W, Liu M, Awasthi S, Abraham-Miranda J, Keske A, Steiner KK, Noel L, Serna AN, Dhillon J *et al* (2021) Macrophage-derived cholesterol contributes to therapeutic resistance in prostate cancer. *Cancer Res* 81: 5477 – 5490

Fizazi K, Scher HI, Molina A, Logothetis CJ, Chi KN, Jones RJ, Staffurth JN, North S, Vogelzang NJ, Saad F *et al* (2012) Abiraterone acetate for treatment of metastatic castration-resistant prostate cancer: final overall survival analysis of the COU-AA-301 randomised, double-blind, placebo-controlled phase 3 study. *Lancet Oncol* 13: 983 – 992

He WW, Sciavolino PJ, Wing J, Augustus M, Hudson P, Meissner PS, Curtis RT, Shell BK, Bostwick DG, Tindall DJ *et al* (1997) A novel human prostate-specific, androgen-regulated homeobox gene (NKX3.1) that maps to 8p21, a region frequently deleted in prostate cancer. *Genomics* 43: 69 – 77

Heidenreich A, Pfister D, Merseburger A, Bartsch G, German Working Group on Castration-Resistant Prostate C (2013) Castration-resistant prostate cancer: where we stand in 2013 and what urologists should know. *Eur Urol* 64: 260 – 265

Hussain M, Mateo J, Fizazi K, Saad F, Shore N, Sandhu S, Chi KN, Sartor O, Agarwal N, Olmos D *et al* (2020) Survival with Olaparib in metastatic castration-resistant prostate cancer. *N Engl J Med* 383: 2345 – 2357

Karthaus WR, Iaquinta PJ, Drost J, Gracanin A, van Boxtel R, Wongvipat J, Dowling CM, Gao D, Begthel H, Sachs N *et al* (2014) Identification of

multipotent luminal progenitor cells in human prostate organoid cultures. *Cell* 159: 163 – 175

Koh MY, Spivak-Kroizman T, Venturini S, Welsh S, Williams RR, Kirkpatrick DL, Powis G (2008) Molecular mechanisms for the activity of PX-478, an antitumor inhibitor of the hypoxia-inducible factor-1alpha. *Mol Cancer Ther* 7: 90 – 100

Liberzon A, Birger C, Thorvaldsdottir H, Ghandi M, Mesirov JP, Tamayo P (2015) The molecular signatures Database (MSigDB) hallmark gene set collection. *Cell Syst* 1: 417 – 425

Loehr A, Hussain A, Patnaik A, Bryce AH, Castellano D, Font A, Shapiro J, Zhang J, Sautois B, Vogelzang NJ *et al* (2022) Emergence of BRCA reversion mutations in patients with metastatic castration-resistant prostate cancer after treatment with Rucaparib. *Eur Urol* 83: 200 – 209

Lopez-Bujanda ZA, Obradovic A, Nirschl TR, Crowley L, Macedo R, Papachristodoulou A, O'Donnell T, Laserson U, Zarif JC, Reshef R *et al* (2021) TGM4: an immunogenic prostate-restricted antigen. *J Immunother Cancer* 9: e001649

Mou T, Deng W, Gu F, Pawitan Y, Vu TN (2019) Reproducibility of methods to detect differentially expressed genes from single-cell RNA sequencing. *Front Genet* 10: 1331

Mulholland DJ, Tran LM, Li Y, Cai H, Morim A, Wang S, Plaisier S, Garraway IP, Huang J, Graeber TG *et al* (2011) Cell autonomous role of PTEN in regulating castration-resistant prostate cancer growth. *Cancer Cell* 19: 792 – 804

Palayoor ST, Mitchell JB, Cerna D, Degraff W, John-Aryankalayil M, Coleman CN (2008) PX-478, an inhibitor of hypoxia-inducible factor-1alpha, enhances radiosensitivity of prostate carcinoma cells. *Int J Cancer* 123: 2430 – 2437

Palazon A, Goldrath AW, Nizet V, Johnson RS (2014) HIF transcription factors, inflammation, and immunity. *Immunity* 41: 518 – 528

Parker C, Castro E, Fizazi K, Heidenreich A, Ost P, Procopio G, Tombal B, Gillessen S, clinicalguidelines@esmo.org EGCEa (2020) Prostate cancer: ESMO clinical practice guidelines for diagnosis, treatment and follow-up. *Ann Oncol* 31: 1119 – 1134

Ratnacaram CK, Teletin M, Jiang M, Meng X, Chambon P, Metzger D (2008) Temporally controlled ablation of PTEN in adult mouse prostate epithelium generates a model of invasive prostatic adenocarcinoma. *Proc Natl Acad Sci USA* 105: 2521 – 2526

Rebello RJ, Oing C, Knudsen KE, Loeb S, Johnson DC, Reiter RE, Gillessen S, Van der Kwast T, Bristow RG (2021) Prostate cancer. *Nat Rev Dis Primers* 7: 9

Rennie PS, Bruchovsky N, Leco KJ, Sheppard PC, McQueen SA, Cheng H, Snoek R, Hamel A, Bock ME, MacDonald BS *et al* (1993) Characterization of two cis-acting DNA elements involved in the androgen regulation of the probasin gene. *Mol Endocrinol* 7: 23 – 36

Resnick MJ, Koyama T, Fan KH, Albertsen PC, Goodman M, Hamilton AS, Hoffman RM, Potosky AL, Stanford JL, Stroup AM *et al* (2013) Long-term functional outcomes after treatment for localized prostate cancer. *N Engl J Med* 368: 436 – 445

Robinson D, Van Allen EM, Wu YM, Schultz N, Lonigro RJ, Mosquera JM, Montgomery B, Taplin ME, Pritchard CC, Attard G *et al* (2015) Integrative clinical genomics of advanced prostate cancer. *Cell* 162: 454

Shappell SB, Thomas GV, Roberts RL, Herbert R, Ittmann MM, Rubin MA, Humphrey PA, Sundberg JP, Rozengurt N, Barrios R *et al* (2004) Prostate pathology of genetically engineered mice: definitions and classification. The consensus report from the Bar Harbor meeting of the mouse models of human cancer consortium prostate pathology committee. *Cancer Res* 64: 2270 – 2305

Siegel RL, Miller KD, Jemal A (2017) Cancer statistics, 2017. *CA Cancer J Clin* 67: 7 – 30

Spans L, Helsen C, Clinckemalie L, Van den Broeck T, Prekovic S, Joniau S, Lerut E, Claessens F (2014) Comparative genomic and transcriptomic analyses of LNCaP and C4-2B prostate cancer cell lines. *PLoS One* 9: e90002

Subramanian A, Tamayo P, Mootha VK, Mukherjee S, Ebert BL, Gillette MA, Paulovich A, Pomeroy SL, Golub TR, Lander ES *et al* (2005) Gene set enrichment analysis: a knowledge-based approach for interpreting genome-wide expression profiles. *Proc Natl Acad Sci USA* 102: 15545 – 15550

Tang F, Xu D, Wang S, Wong CK, Martinez-Fundichely A, Lee CJ, Cohen S, Park J, Hill CE, Eng K *et al* (2022) Chromatin profiles classify castration-resistant prostate cancers suggesting therapeutic targets. *Science* 376: eabe1505

Wang S, Gao J, Lei Q, Rozengurt N, Pritchard C, Jiao J, Thomas GV, Li G, Roy-Burman P, Nelson PS *et al* (2003) Prostate-specific deletion of the murine Pten tumor suppressor gene leads to metastatic prostate cancer. *Cancer Cell* 4: 209 – 221

Wang X, Cai J, Zhao L, Zhang D, Xu G, Hu J, Zhang T, Jin M (2021) NUMB suppression by miR-9-5P enhances CD44(+) prostate cancer stem cell growth and metastasis. *Sci Rep* 11: 11210

Zhang Z, Karthaus WR, Lee YS, Gao VR, Wu C, Russo JW, Liu M, Mota JM, Abida W, Linton E *et al* (2020) Tumor microenvironment-derived NRG1 promotes antiandrogen resistance in prostate cancer. *Cancer Cell* 38: 279 – 296.e9

License: This is an open access article under the terms of the Creative Commons Attribution License, which permits use, distribution and reproduction in any medium, provided the original work is properly cited.

 © 2023 The Authors

Expanded View Figures

Figure EV1. Characterization of castrated Pten$^{(i)pe-/-}$ mice.

A–C Weight of bulbocavernosus muscles (A), seminal vesicles (B), and prostates (C) of Pten$^{L2/L2}$ (control) and Pten$^{(i)pe-/-}$ mice, sham-operated (sham), or castrated (CTX) at 3 months AGI and analyzed at 1 month after surgery. $N = 3$–12 mice/condition. Data presented are mean ± SEM. P-values were determined by one-way ANOVA followed by a *post hoc* Tuckey test.

D Gland areas in DLP of sham and CTX control Pten$^{L2/L2}$ and Pten$^{(i)pe-/-}$ mice. $N = 3$ mice/condition. Five glands per mouse were quantified. P-values were determined by one-way ANOVA followed by a *post hoc* Tuckey test.

E UMAP depicting the unbiased clusters obtained from scRNA-seq analysis of cells from prostates of Pten$^{(i)pe-/-}$ mice, sham or CTX at 3 months AGI and analyzed 1 month after surgery.

F Stacked bars showing the proportion of the identified cell populations in the prostates of sham and CTX Pten$^{(i)pe-/-}$ mice, based on the scRNA-sequencing analysis.

G Feature plots depicting the expression of Epcam, Ptprc, and Vim in the various cell clusters.

H UMAP depicting luminal-A and luminal-C cells obtained from scRNA-seq analysis of prostates of Pten$^{(i)pe-/-}$ mice, sham or CTX at 3 months AGI and analyzed 1 month after surgery.

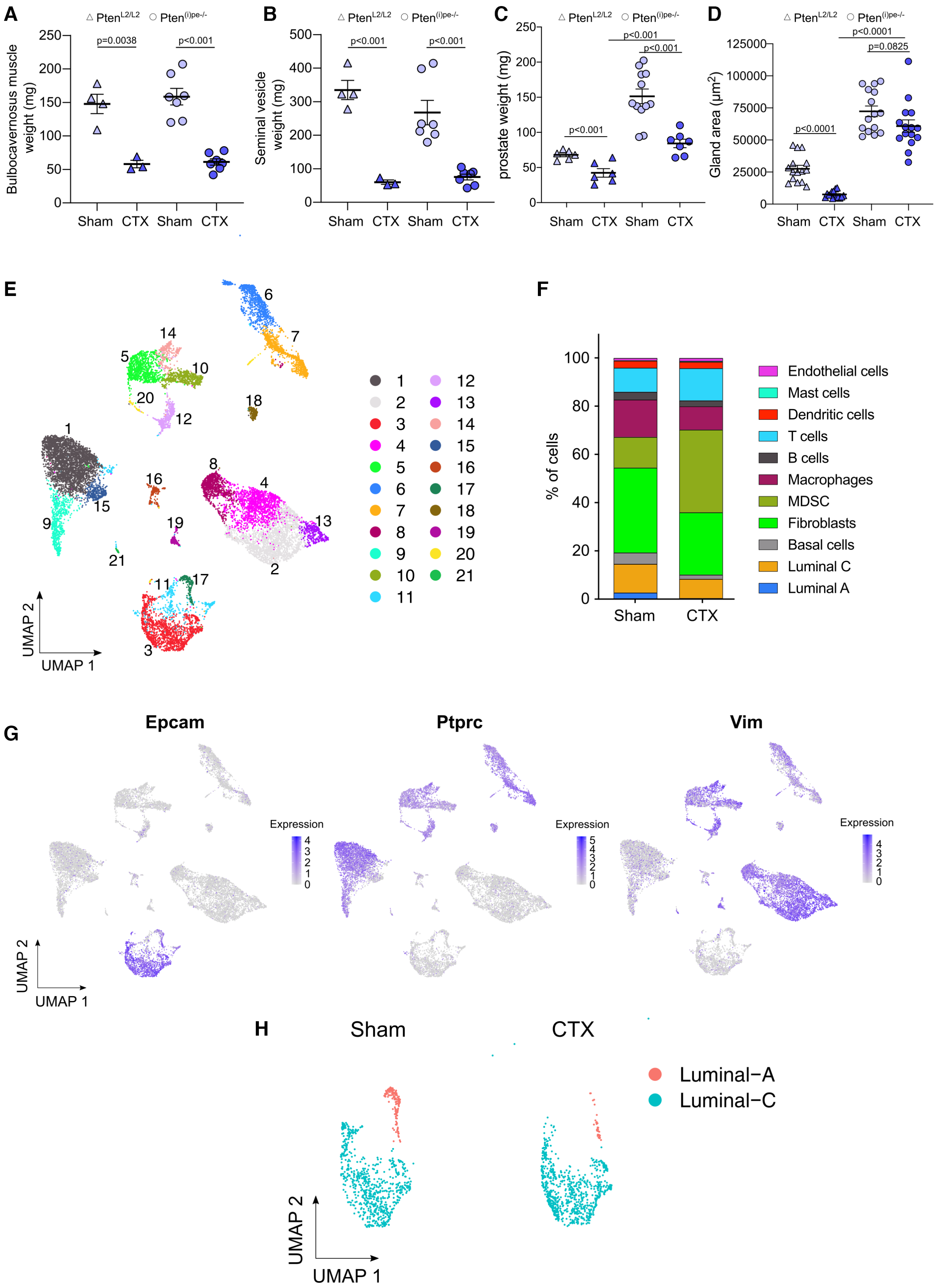

Figure EV1.

© 2023 The Authors

Figure EV2. Characterization of castrated Pten$^{(i)pe-/-}$ and Pten/Hif1a$^{(i)pe-/-}$ mice.

A, B Seminal vesicles weight of Pten$^{(i)pe-/-}$ mice (A) and Pten/Hif1a$^{(i)pe-/-}$ mice (B), sham-operated (sham), or castrated (CTX) at 3 months AGI and analyzed 3 days after surgery. (A) $N = 3$ mice/condition. (B) $N = 3$–4 mice/condition. Data presented are mean ± SEM. *P*-values were determined by one-way ANOVA followed by a *post hoc* Tuckey test.

C Representative immunohistochemical detection of AR in the DLP of Pten$^{(i)pe-/-}$ and Pten/Hif1a$^{(i)pe-/-}$ mice 1 day after sham operation, and 1–3 days after castration, performed 3 months AGI. Scale bars: 100 µm. $N = 3$ mice/condition.

D, E Percentage of Ki67 positive epithelial cells in DLP of Pten$^{(i)pe-/-}$ mice (D) and Pten/Hif1a $^{(i)pe-/-}$ mice (E) 1 day after sham operation and 1, 2, 3 or 30 days after castration, performed at 3 months AGI. $N = 3$–4 mice/condition. Five glands per mouse were quantified. *P*-values were determined by unpaired *t*-tests.

F–H Representative immunohistochemical detection of cleaved PARP (CPARP) in DLP of sham and CTX Pten$^{(i)pe-/-}$ and Pten/Hif1a$^{(i)pe-/-}$ mice (F) and quantification of CPARP-positive prostatic epithelial cells in DLP of Pten$^{(i)pe-/-}$mice (G) and Pten/Hif1a$^{(i)pe-/-}$ (H) mice. Surgery was performed at 3 months AGI. Sham mice were analyzed 1 day later and CTX mice 1, 2, 3, and 30 days later. $N = 4$ mice/condition. (F) Scale bars: 100 µm. Five glands per mouse were quantified in (G) and (H). Data presented are mean ± SEM. *P*-values were determined by unpaired *t*-tests.

© 2023 The Authors

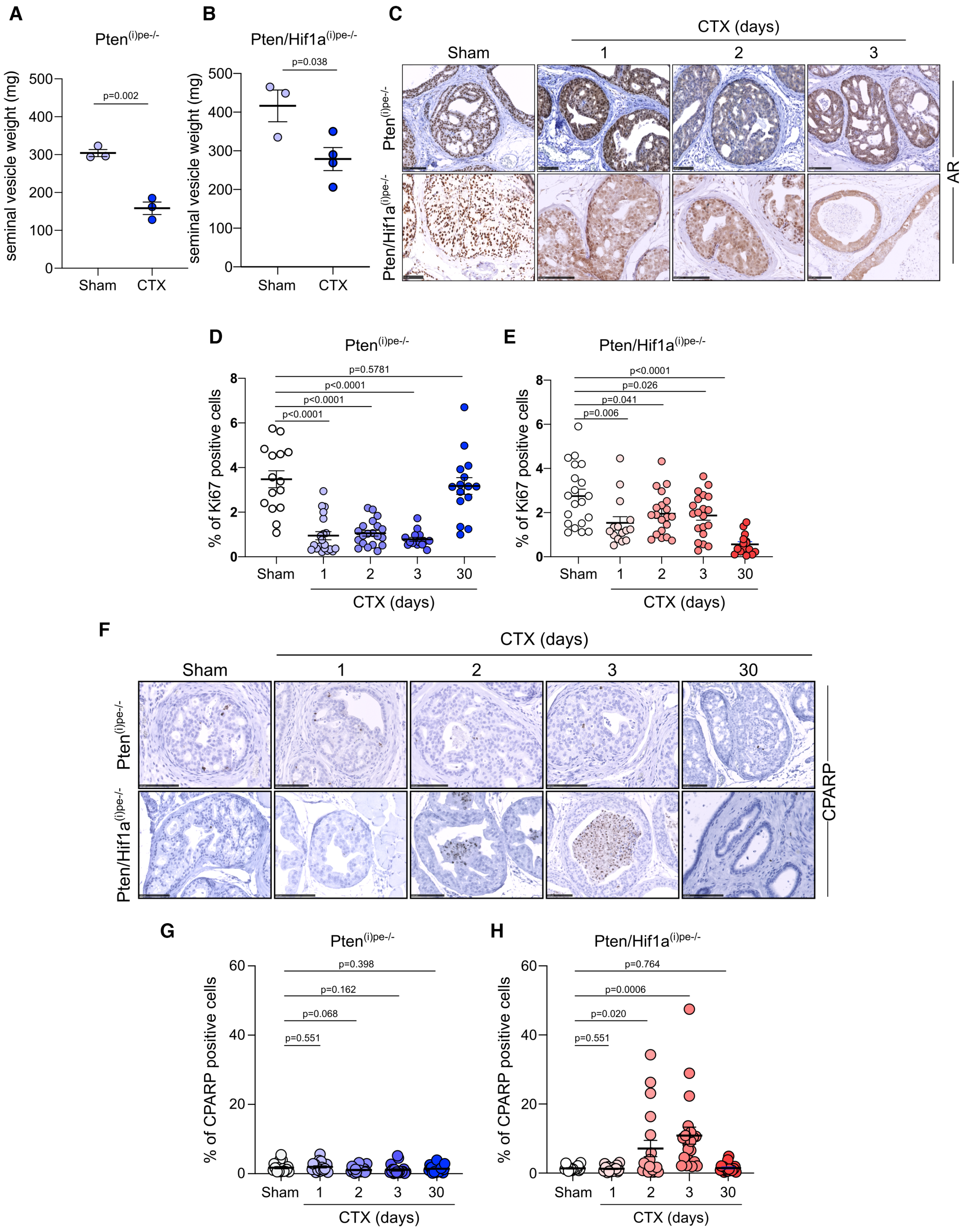

Figure EV2.

 © 2023 The Authors

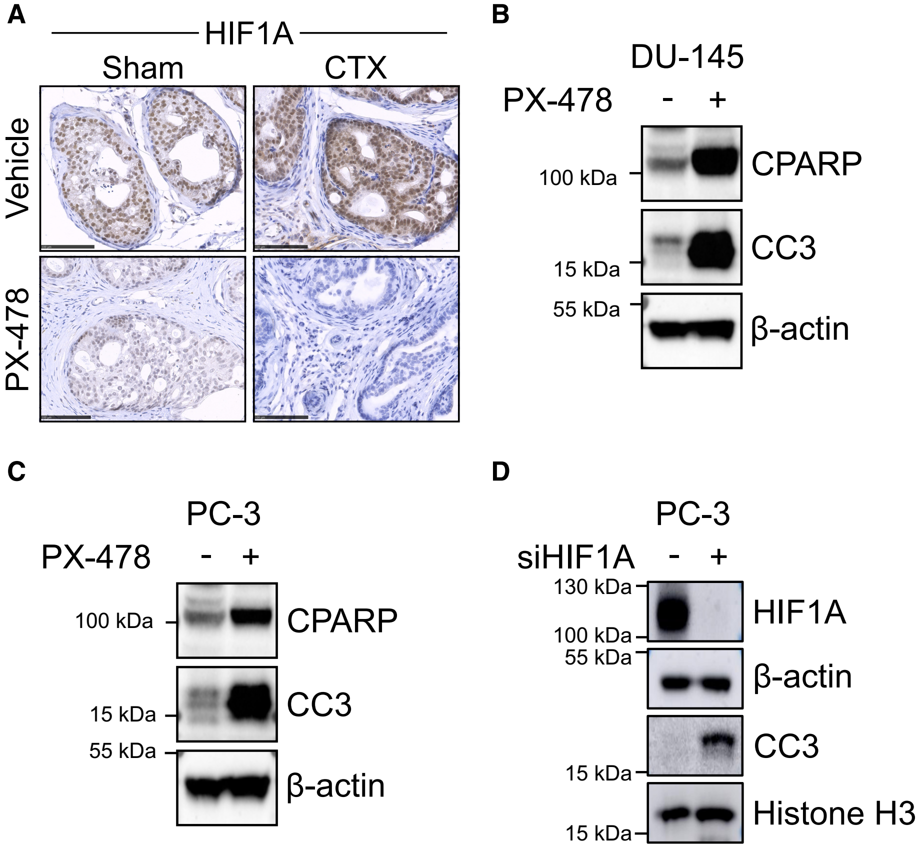

Figure EV3. Effects of HIF1A inhibition on prostate of castrated Pten$^{(i)pe-/-}$ mice and human CRPC cell lines.

A Representative immunohistochemical analysis of HIF1A in DLP of sham and CTX Pten$^{(i)pe-/-}$ mice treated with vehicle or PX-478 as described in Fig 4A, and analyzed at day 25. Scale bar: 100 µm. *N* = 3–4 mice per group.

B, C Western blot analysis of CPARP and CC3 in protein lysates of DU-145 (B) and PC-3 (C) human prostate cell lines treated with vehicle (–) or PX-478 (50 µM) for 24 h. β-actin was used as a loading control. Data are representative of two independent biological replicates.

D Western blot analysis of HIF1A and CC3 in protein lysates of PC-3 cells silenced or not for HIF1A. Histone H3 and β-actin were used as a loading control.
