# Supplementary figures and images for: Hypoxia‐inducible factor 1A inhibition overcomes castration resistance of prostate tumors
Source: EMBO Mol Med. 2023 Apr 18;15(6):e17209. doi: 10.15252/emmm.202217209 (PMC10245031; doi:10.15252/emmm.202217209)

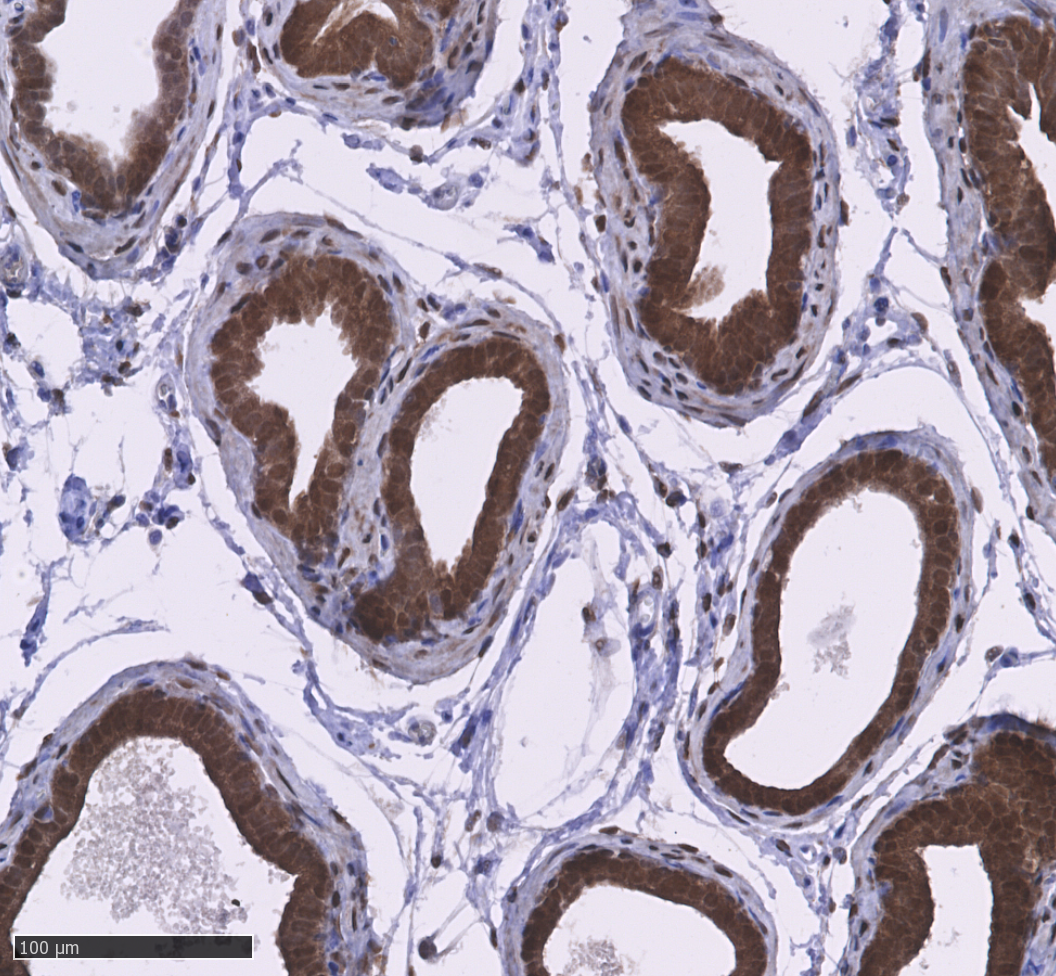

Supplement: Supplementary file 5 — Source Data for Figure 1 [file EMMM-15-e17209-s007.zip › Fig1/1_A/CTX_control_AR 20x.tif]

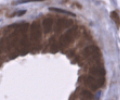

Supplement: Supplementary file 5 — Source Data for Figure 1 [file EMMM-15-e17209-s007.zip › Fig1/1_A/CTX_control_AR zoom.tif]

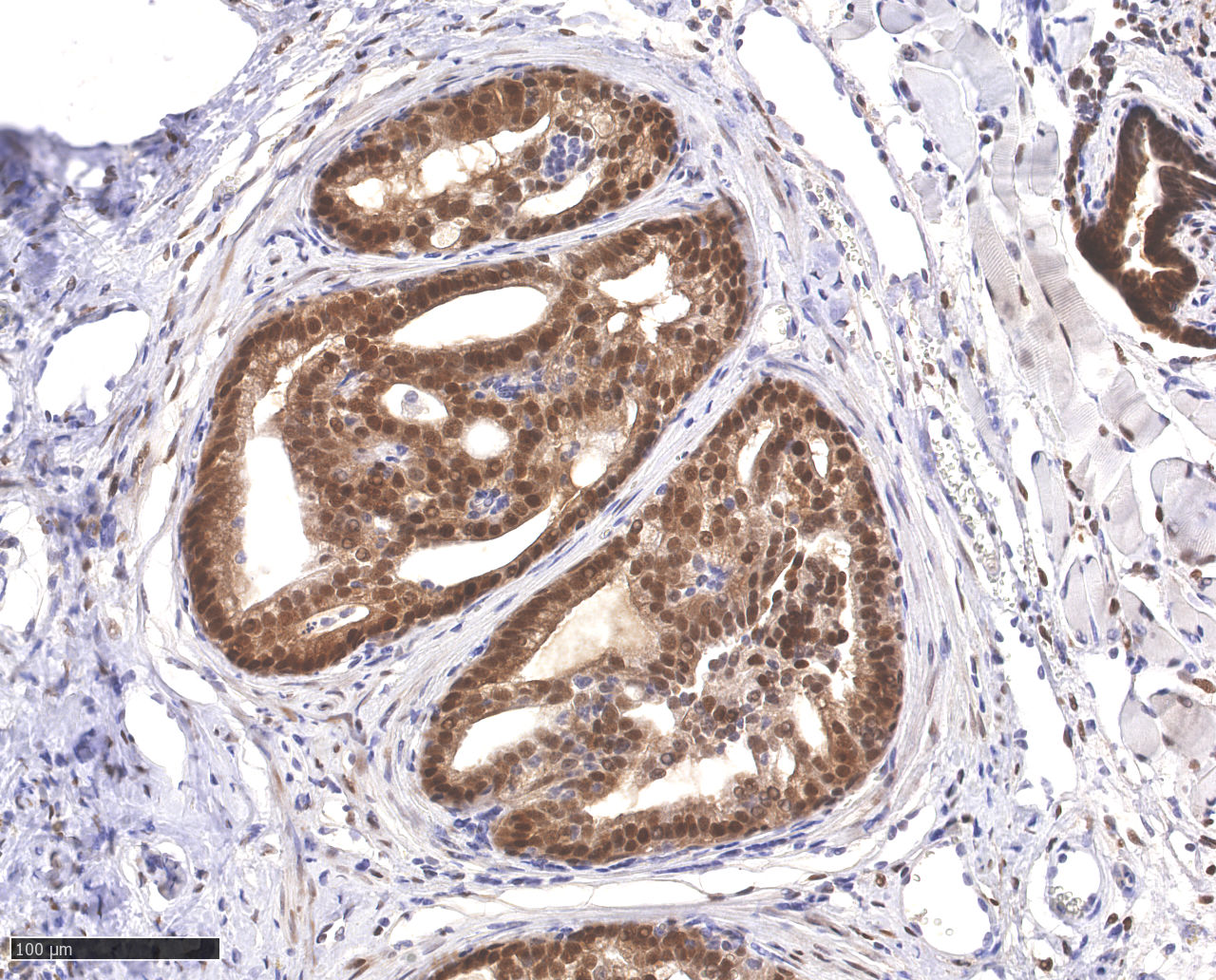

Supplement: Supplementary file 5 — Source Data for Figure 1 [file EMMM-15-e17209-s007.zip › Fig1/1_A/CTX_pten_AR.tiff]

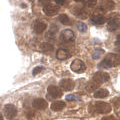

Supplement: Supplementary file 5 — Source Data for Figure 1 [file EMMM-15-e17209-s007.zip › Fig1/1_A/CTX_pten_AR_zoom.tif]

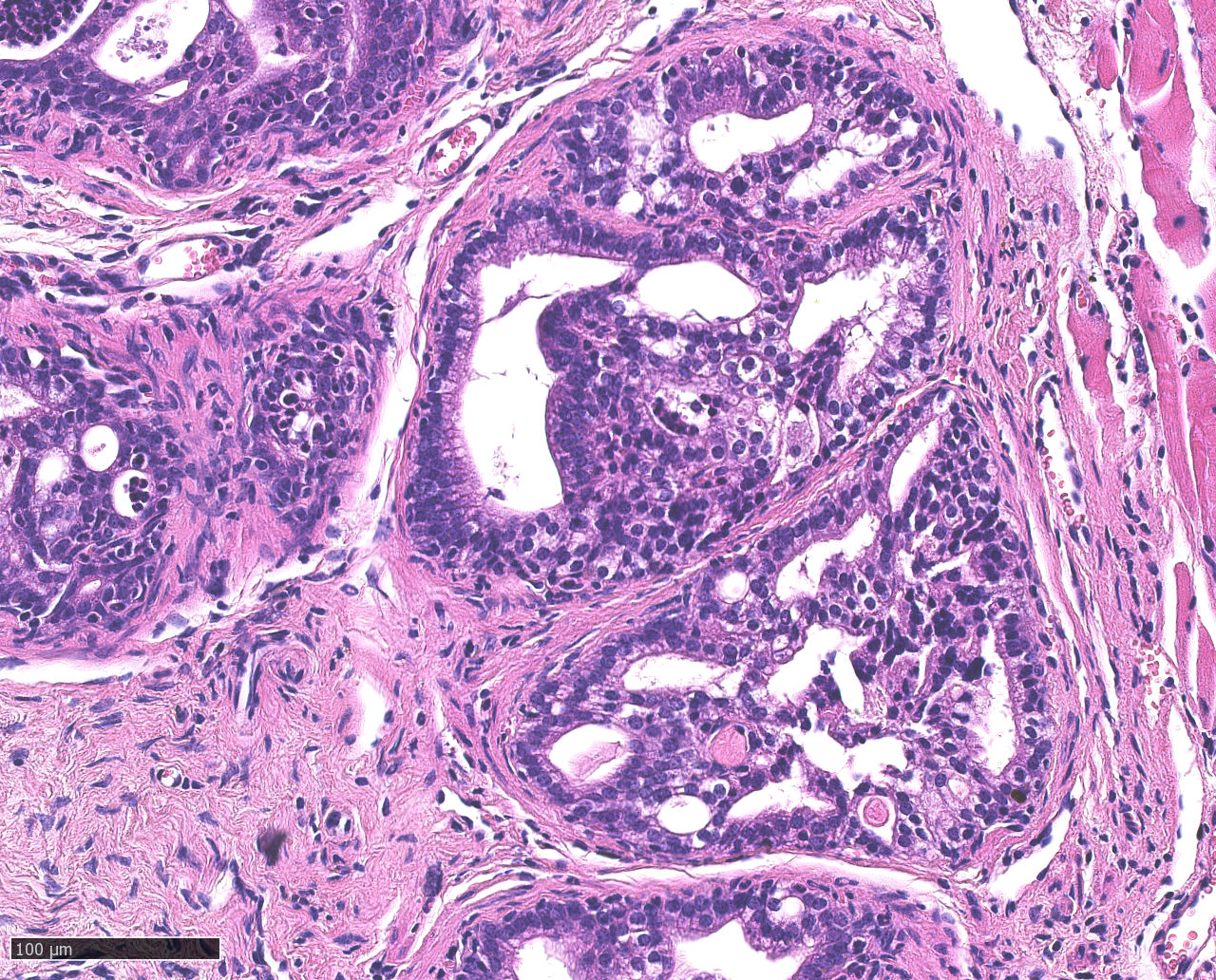

Supplement: Supplementary file 5 — Source Data for Figure 1 [file EMMM-15-e17209-s007.zip › Fig1/1_A/CTX_pten_HE.tiff]

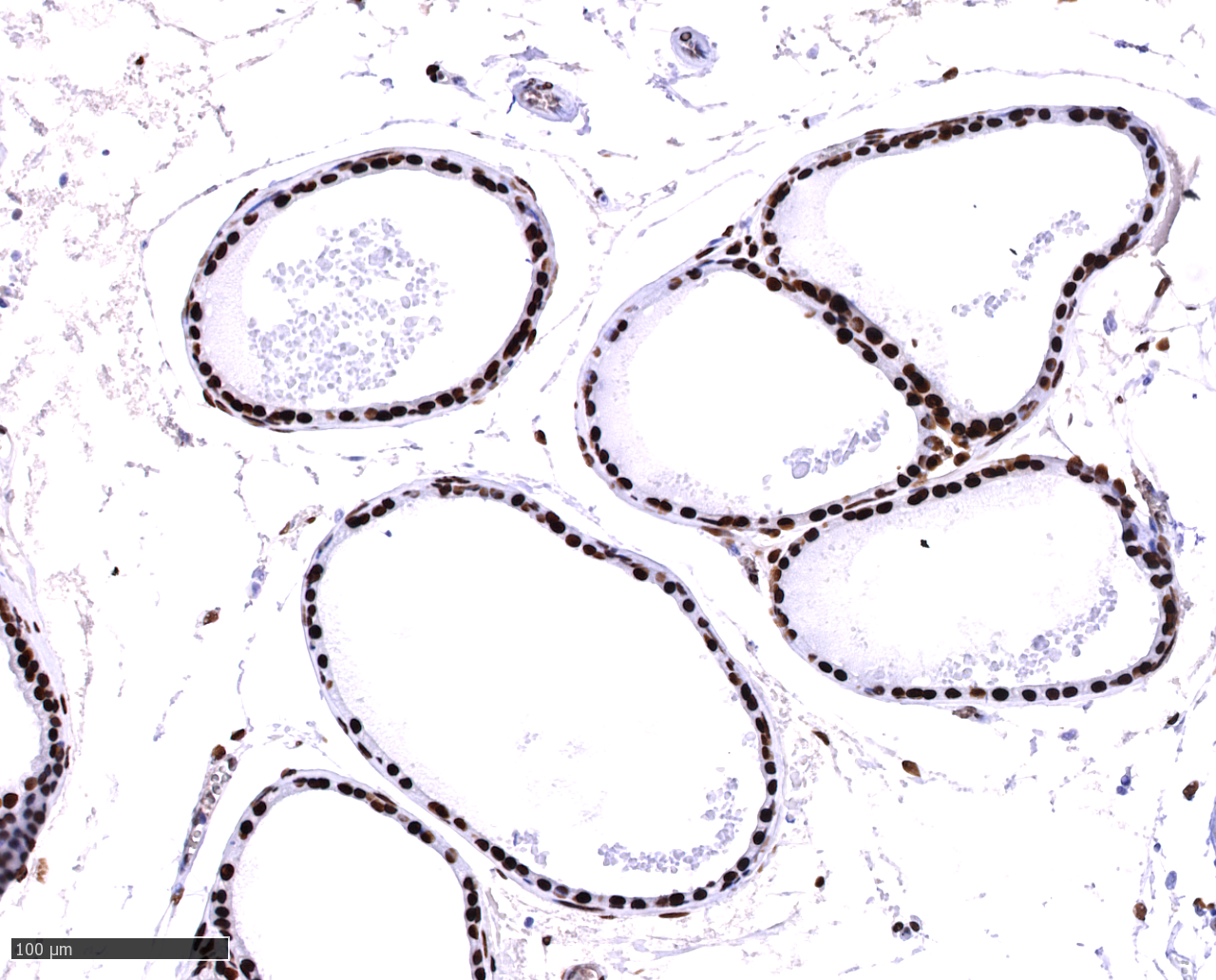

Supplement: Supplementary file 5 — Source Data for Figure 1 [file EMMM-15-e17209-s007.zip › Fig1/1_A/sham_control_AR.tif]

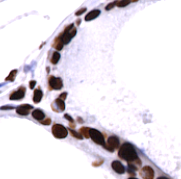

Supplement: Supplementary file 5 — Source Data for Figure 1 [file EMMM-15-e17209-s007.zip › Fig1/1_A/sham_control_AR_zoom.tif]

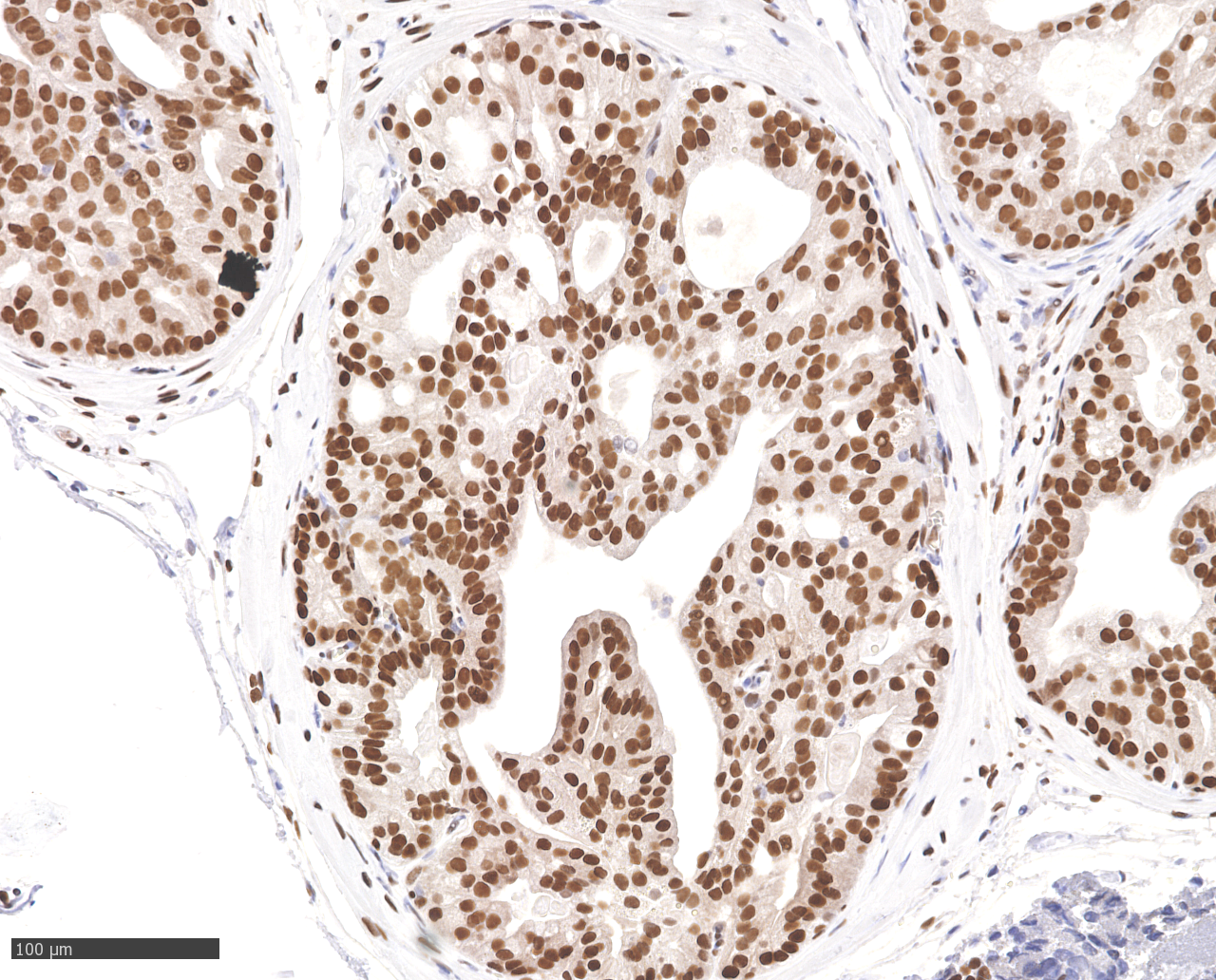

Supplement: Supplementary file 5 — Source Data for Figure 1 [file EMMM-15-e17209-s007.zip › Fig1/1_A/sham_pten_AR.tif]

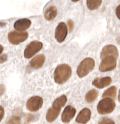

Supplement: Supplementary file 5 — Source Data for Figure 1 [file EMMM-15-e17209-s007.zip › Fig1/1_A/sham_pten_AR_zoom.tif]

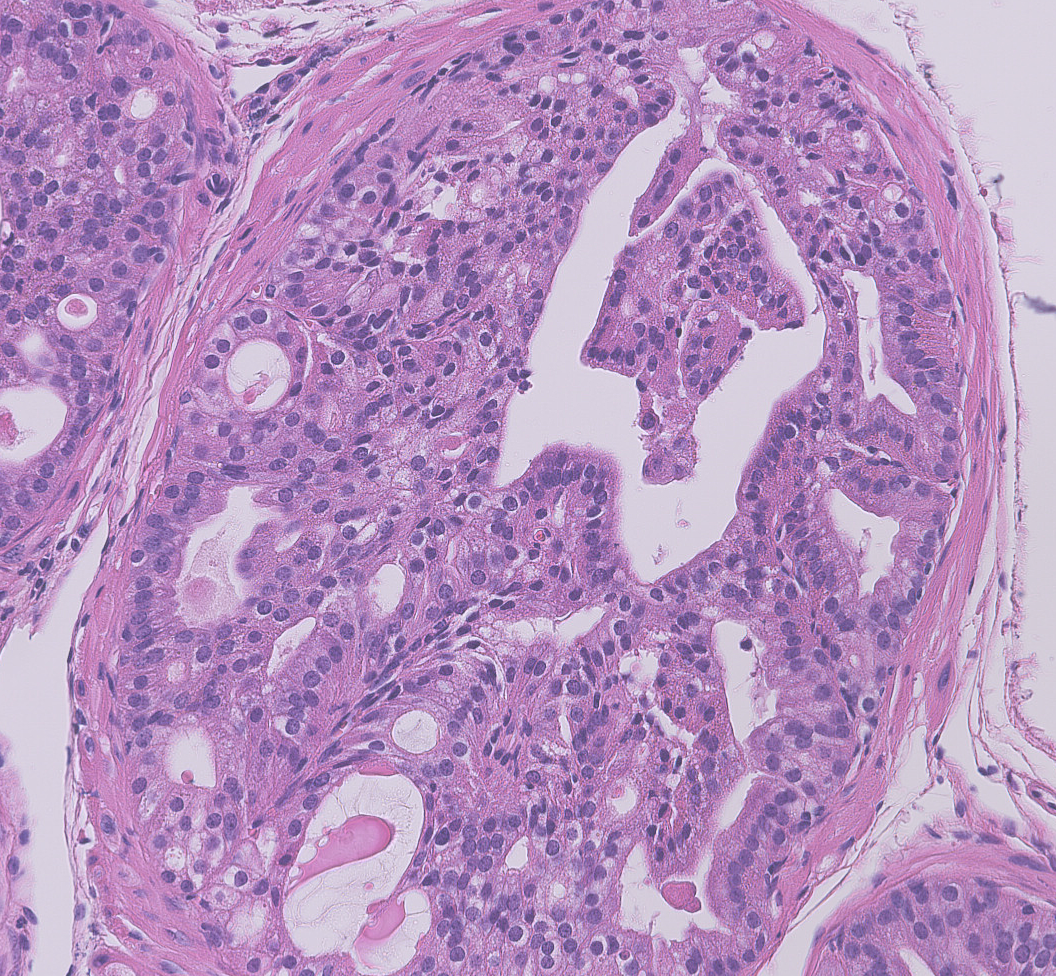

Supplement: Supplementary file 5 — Source Data for Figure 1 [file EMMM-15-e17209-s007.zip › Fig1/1_A/sham_pten_HE_20x.tif]

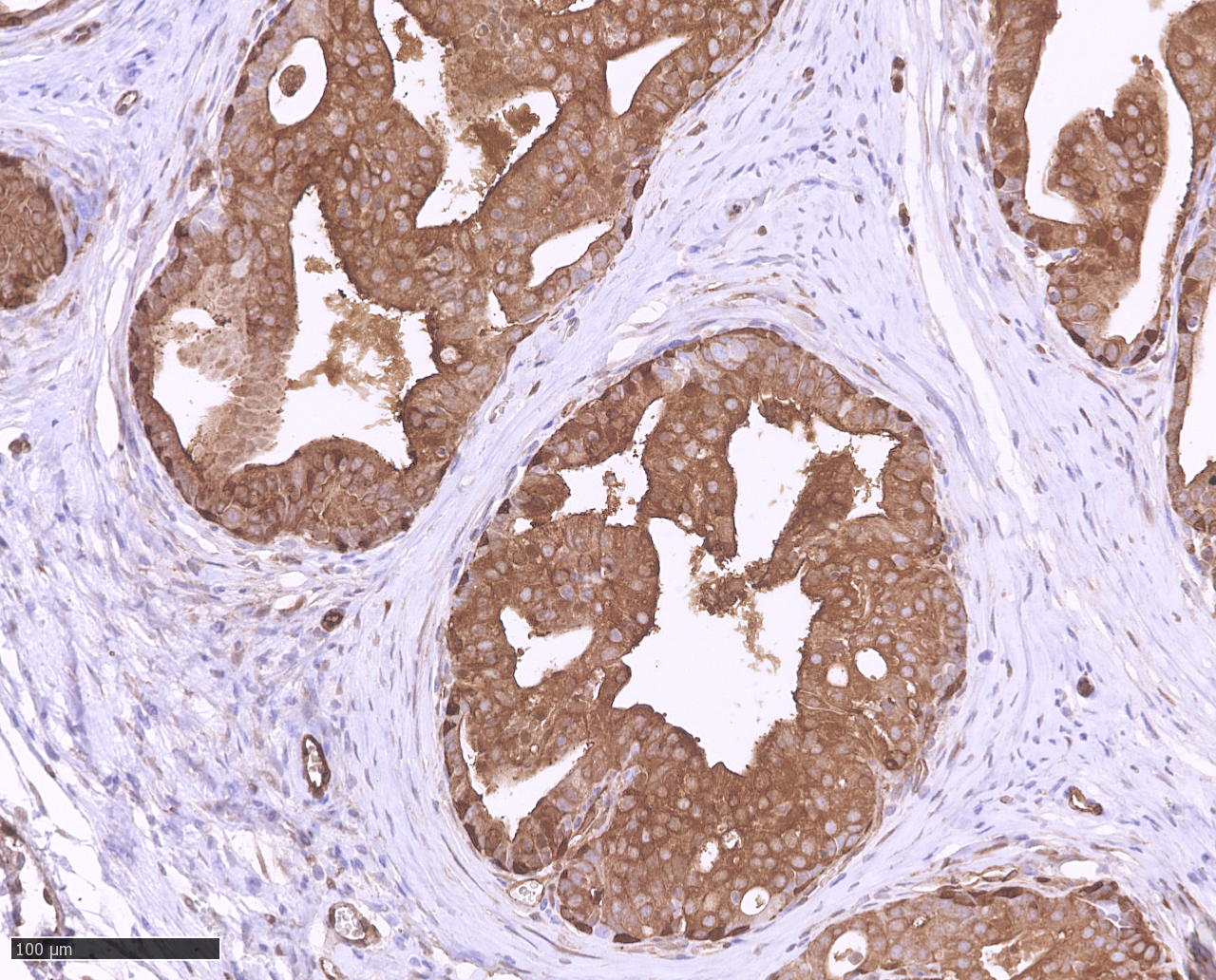

Supplement: Supplementary file 5 — Source Data for Figure 1 [file EMMM-15-e17209-s007.zip › Fig1/1_L/CTX_PTEN_tgm2_20x.tif]

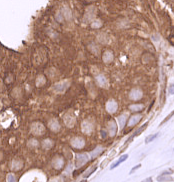

Supplement: Supplementary file 5 — Source Data for Figure 1 [file EMMM-15-e17209-s007.zip › Fig1/1_L/CTX_PTEN_tgm2_zoom.tif]

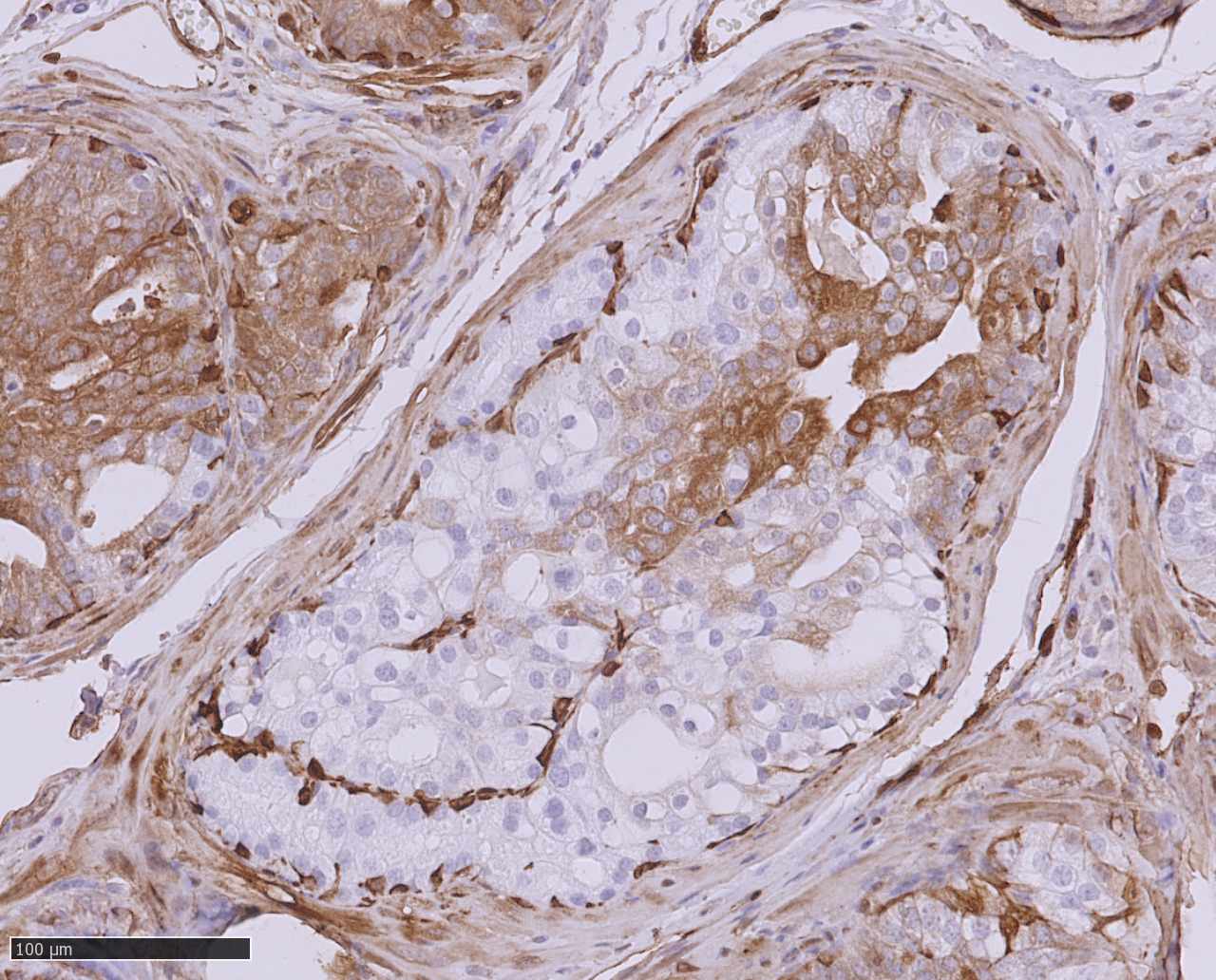

Supplement: Supplementary file 5 — Source Data for Figure 1 [file EMMM-15-e17209-s007.zip › Fig1/1_L/sham_pten_tgm2_20x.tif]

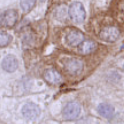

Supplement: Supplementary file 5 — Source Data for Figure 1 [file EMMM-15-e17209-s007.zip › Fig1/1_L/sham_pten_tgm2_20xzoom.tif]

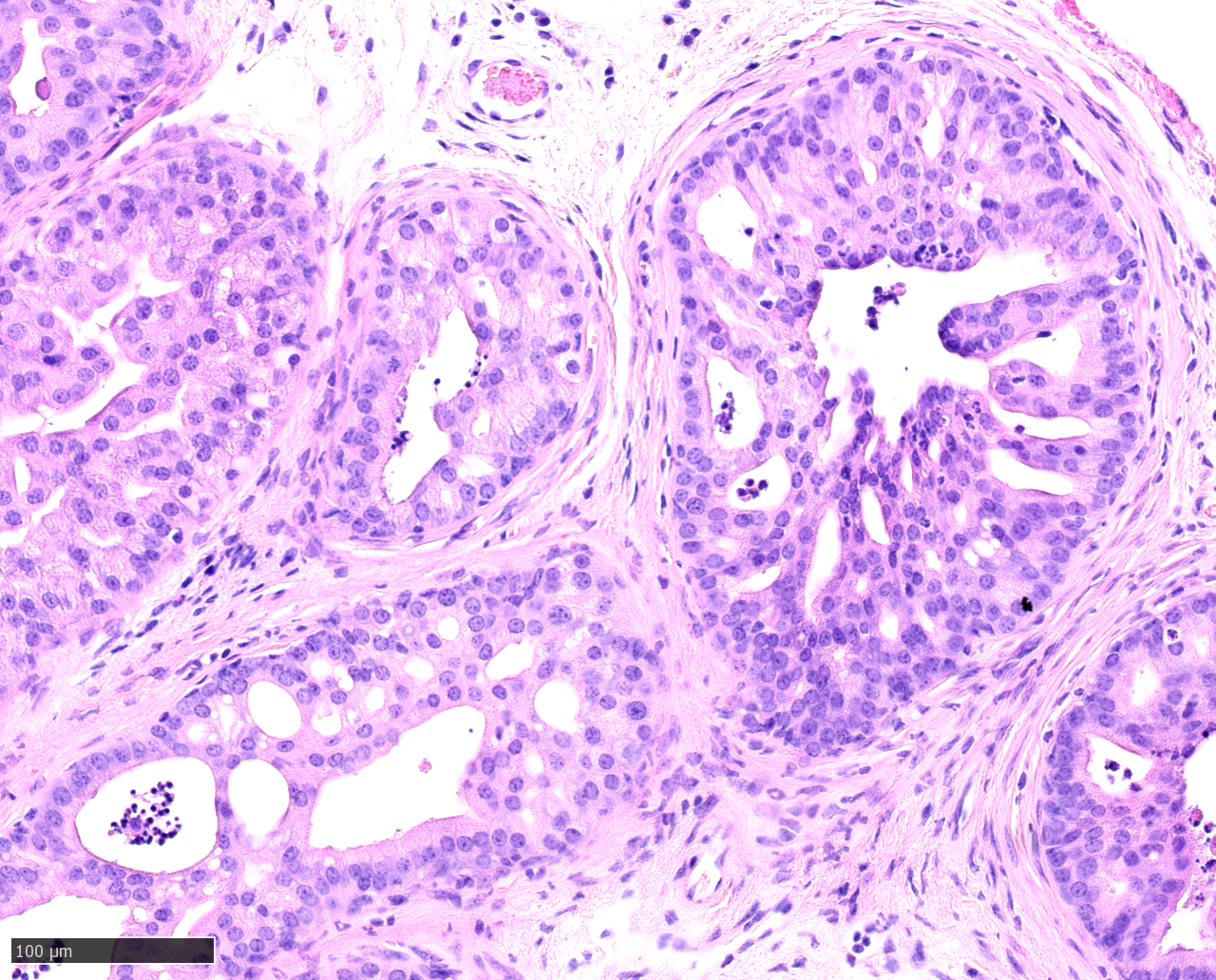

Supplement: Supplementary file 6 — Source Data for Figure 2 [file EMMM-15-e17209-s004.zip › Fig 2/2_B/2.HE_PTENHIF_tg_sham.tif]

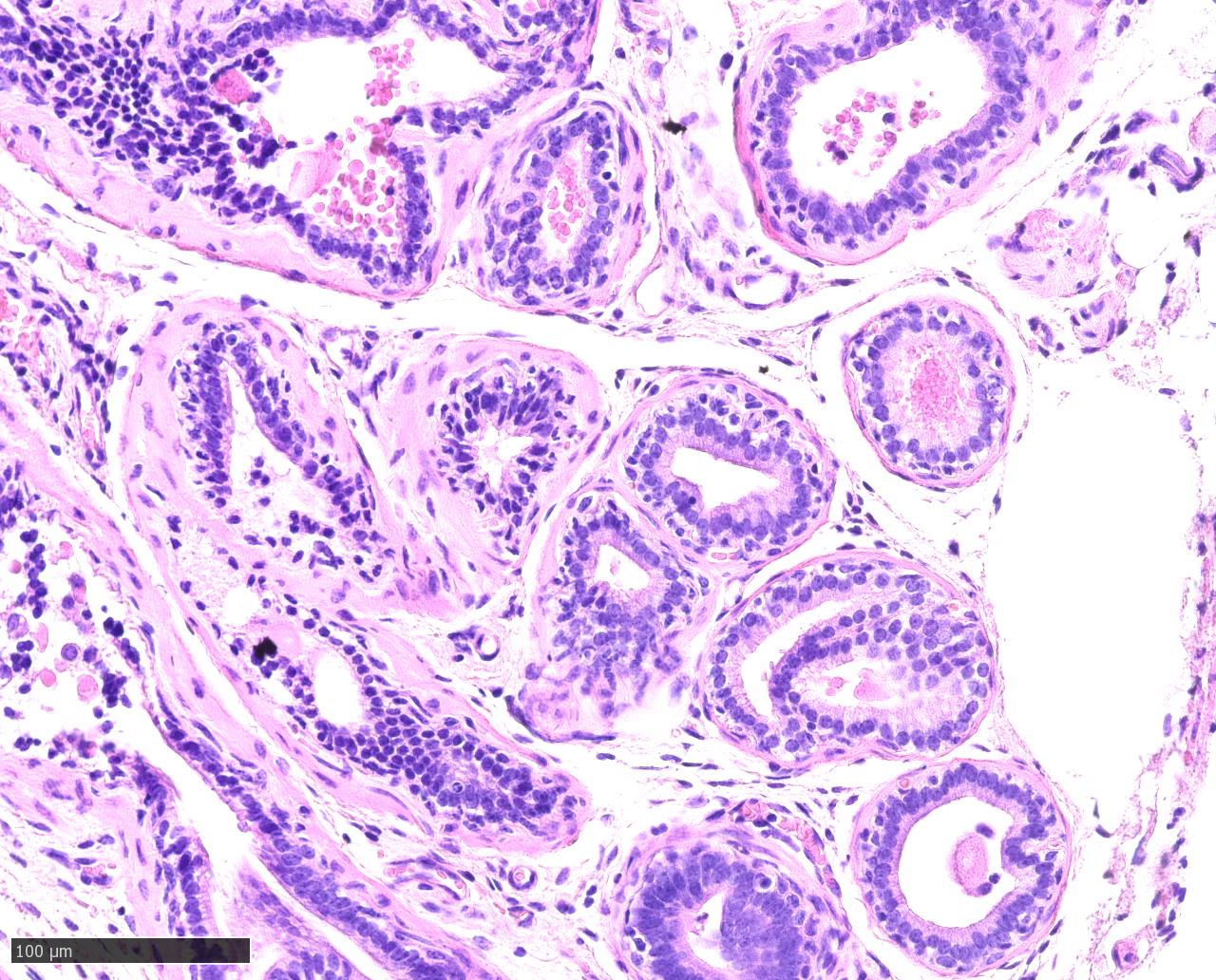

Supplement: Supplementary file 6 — Source Data for Figure 2 [file EMMM-15-e17209-s004.zip › Fig 2/2_B/3.HE_PTENHIF_tg_CTX.tif]

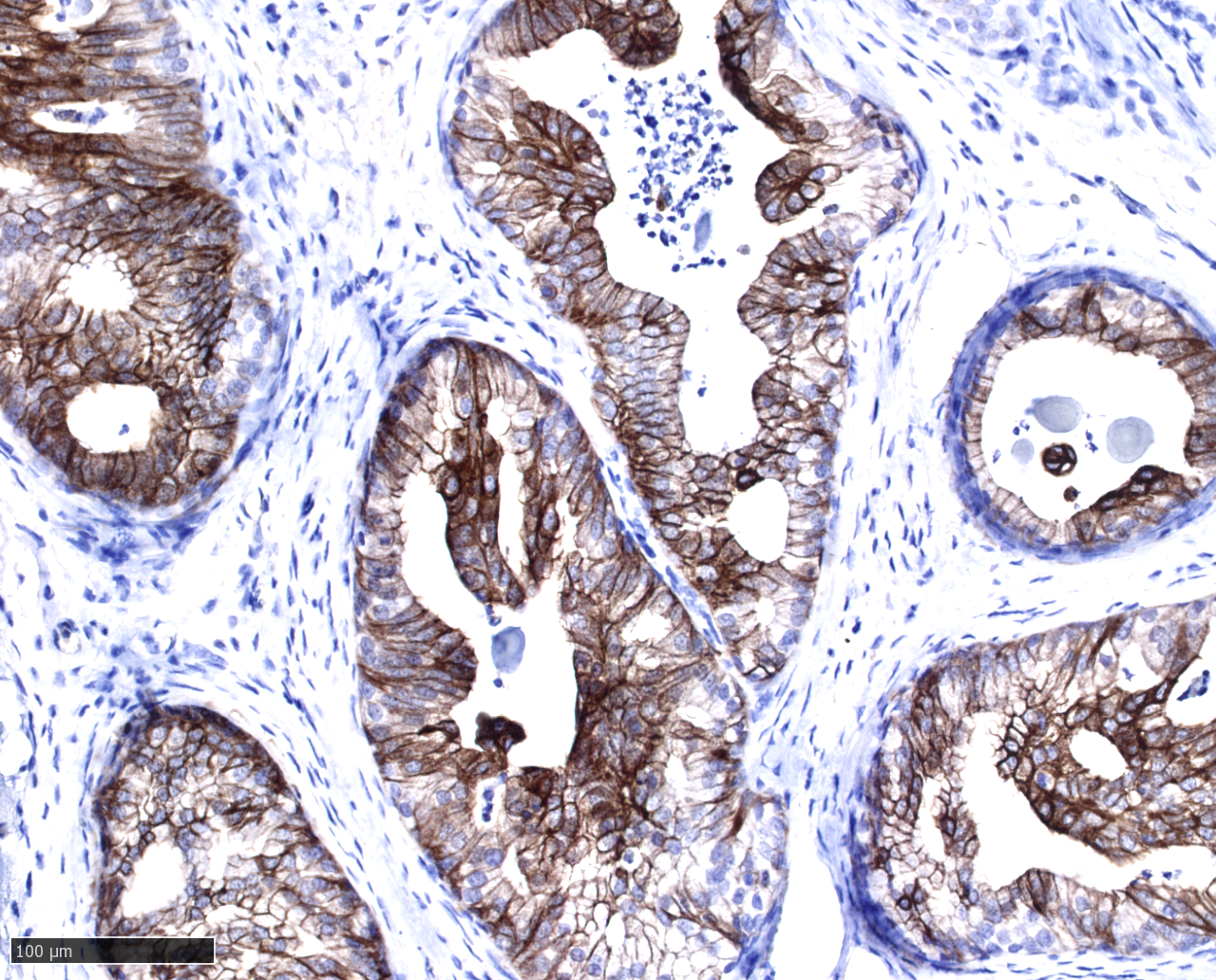

Supplement: Supplementary file 6 — Source Data for Figure 2 [file EMMM-15-e17209-s004.zip › Fig 2/2_E/PTENHIF_SHAM_CTX__trop2.tif]

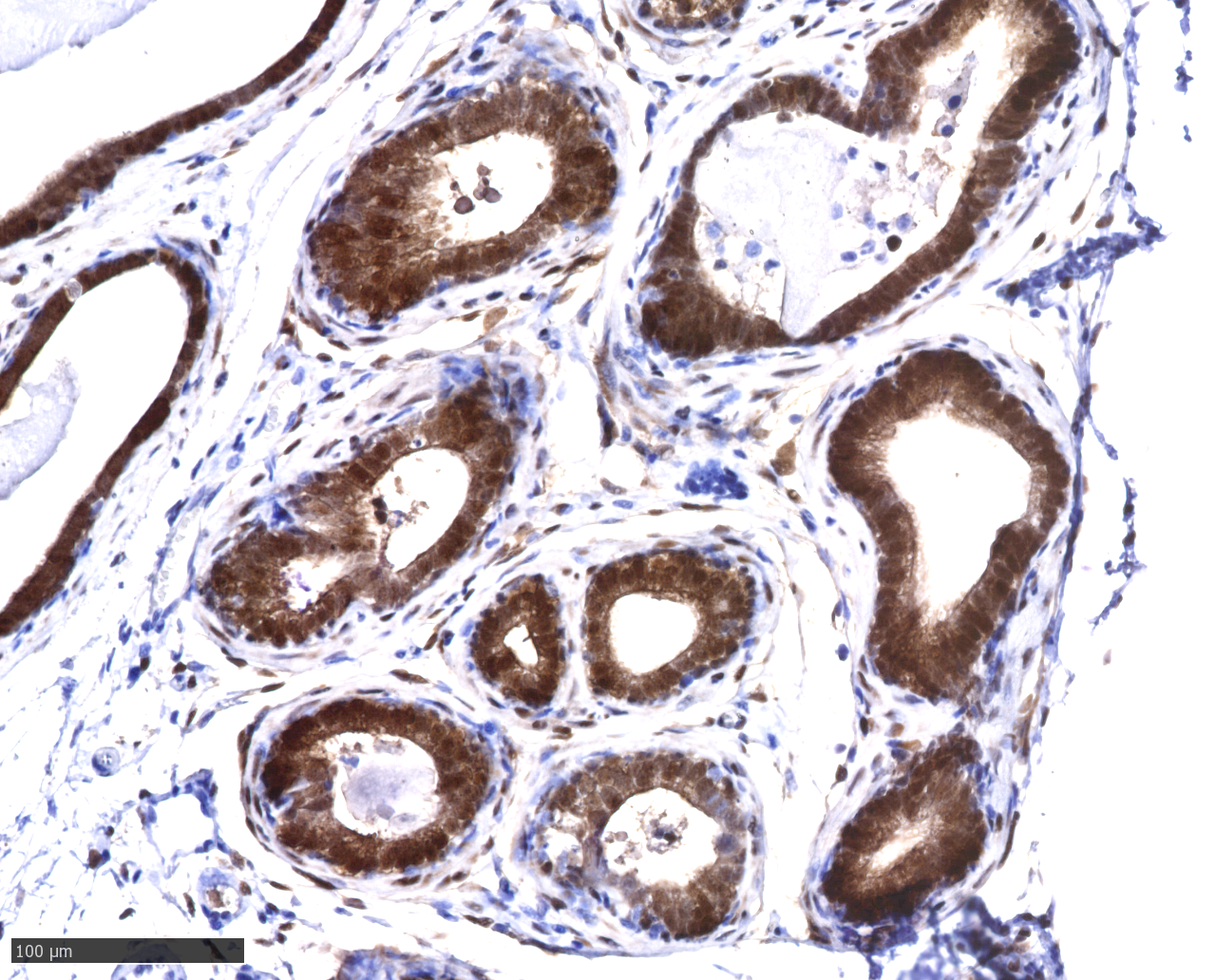

Supplement: Supplementary file 6 — Source Data for Figure 2 [file EMMM-15-e17209-s004.zip › Fig 2/2_E/PTENHIF_Tg_CTX__AR.tif]

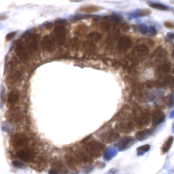

Supplement: Supplementary file 6 — Source Data for Figure 2 [file EMMM-15-e17209-s004.zip › Fig 2/2_E/PTENHIF_Tg_CTX__ARzoom.tif]

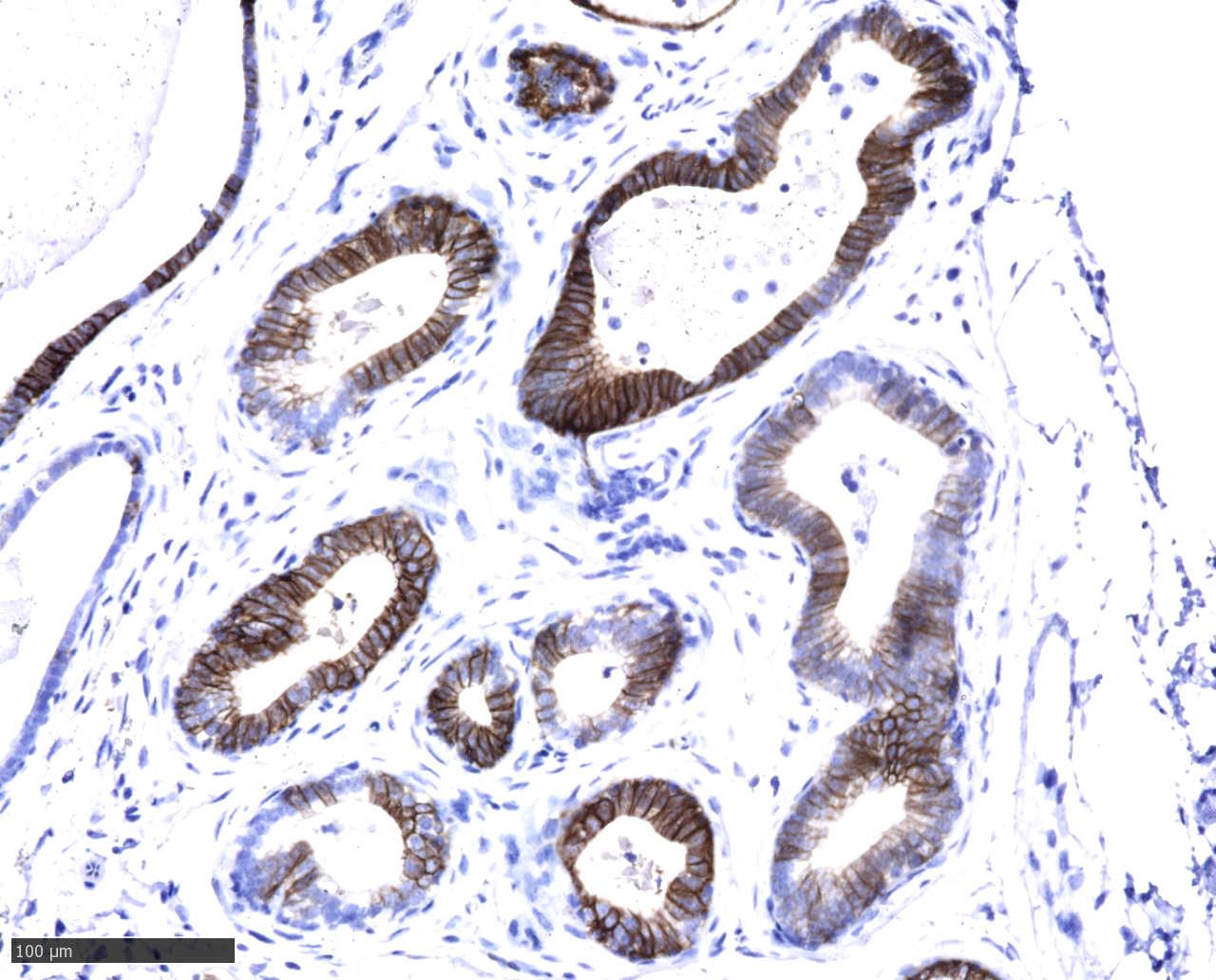

Supplement: Supplementary file 6 — Source Data for Figure 2 [file EMMM-15-e17209-s004.zip › Fig 2/2_E/PTENHIF_Tg_CTX__trop2.tif]

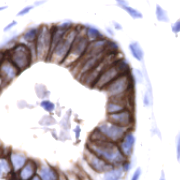

Supplement: Supplementary file 6 — Source Data for Figure 2 [file EMMM-15-e17209-s004.zip › Fig 2/2_E/PTENHIF_Tg_CTX__trop2_zoom.tif]

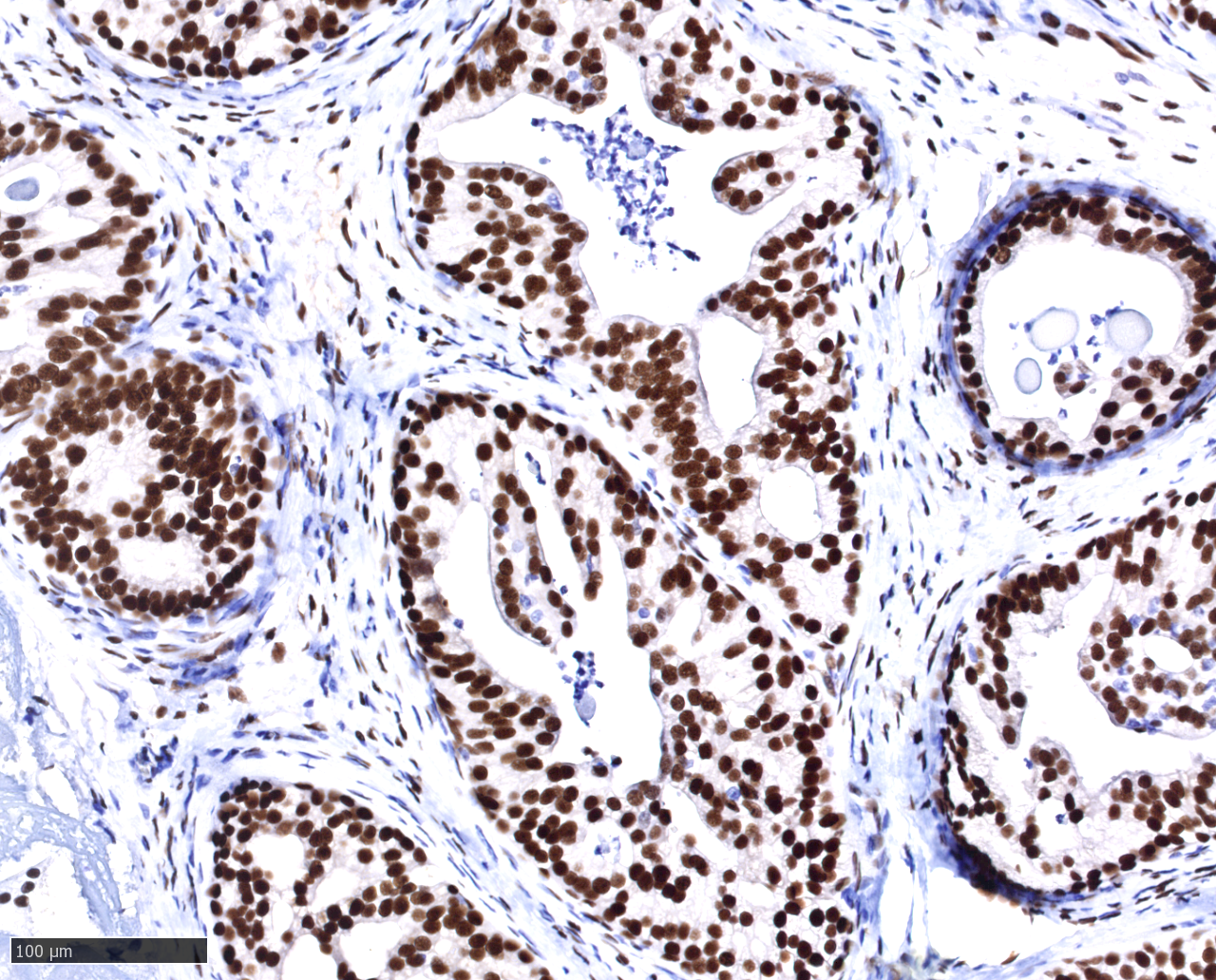

Supplement: Supplementary file 6 — Source Data for Figure 2 [file EMMM-15-e17209-s004.zip › Fig 2/2_E/PTENHIF_Tg_SHAM__AR.tif]

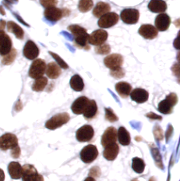

Supplement: Supplementary file 6 — Source Data for Figure 2 [file EMMM-15-e17209-s004.zip › Fig 2/2_E/PTENHIF_Tg_sham__AR_zoom.tif]

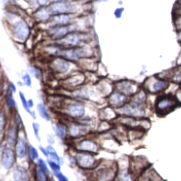

Supplement: Supplementary file 6 — Source Data for Figure 2 [file EMMM-15-e17209-s004.zip › Fig 2/2_E/PTENHIF_Tg_sham__trop2_zoom.tif]

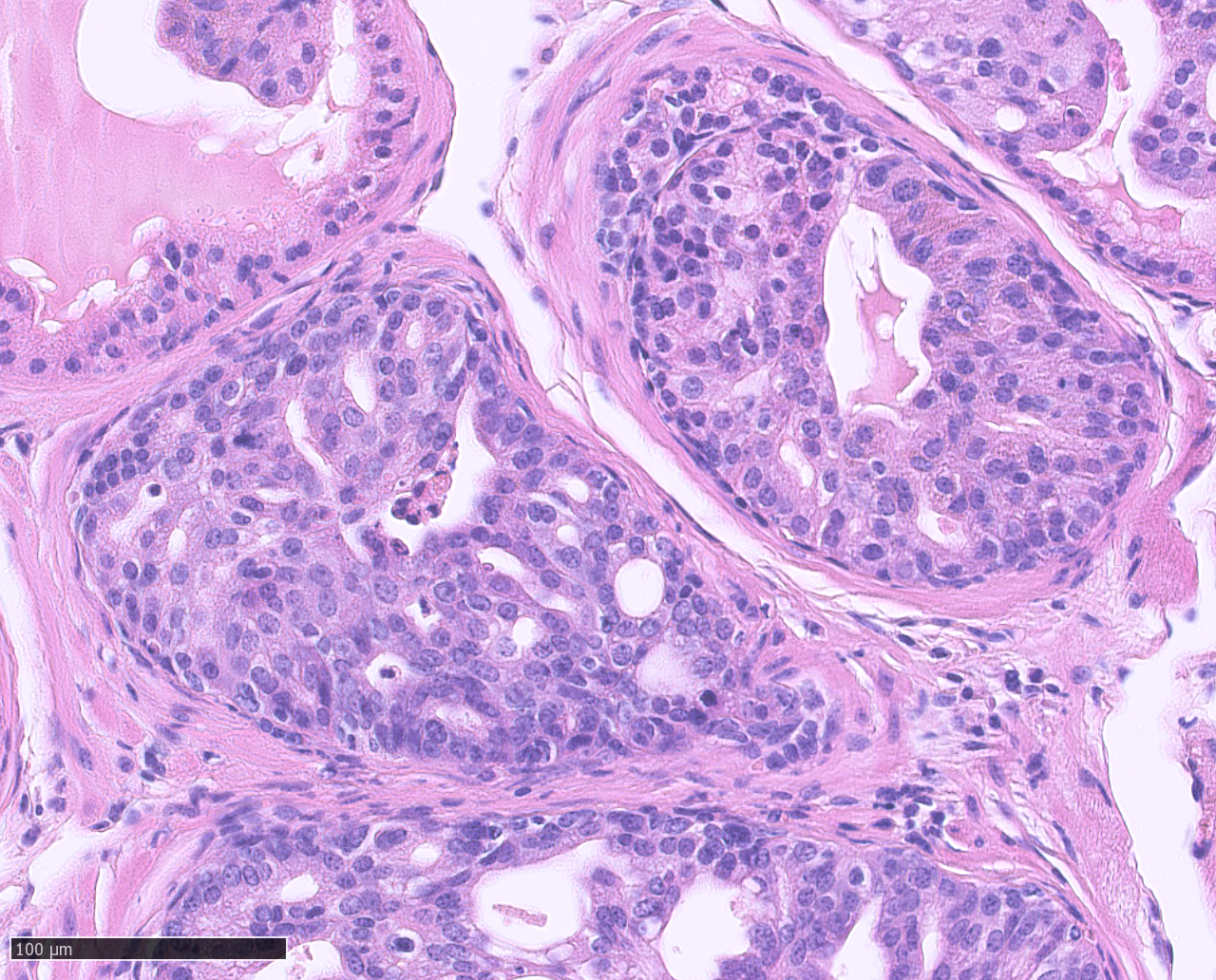

Supplement: Supplementary file 6 — Source Data for Figure 2 [file EMMM-15-e17209-s004.zip › Fig 2/2_F/PTENHIF_CTX_24h.tif]

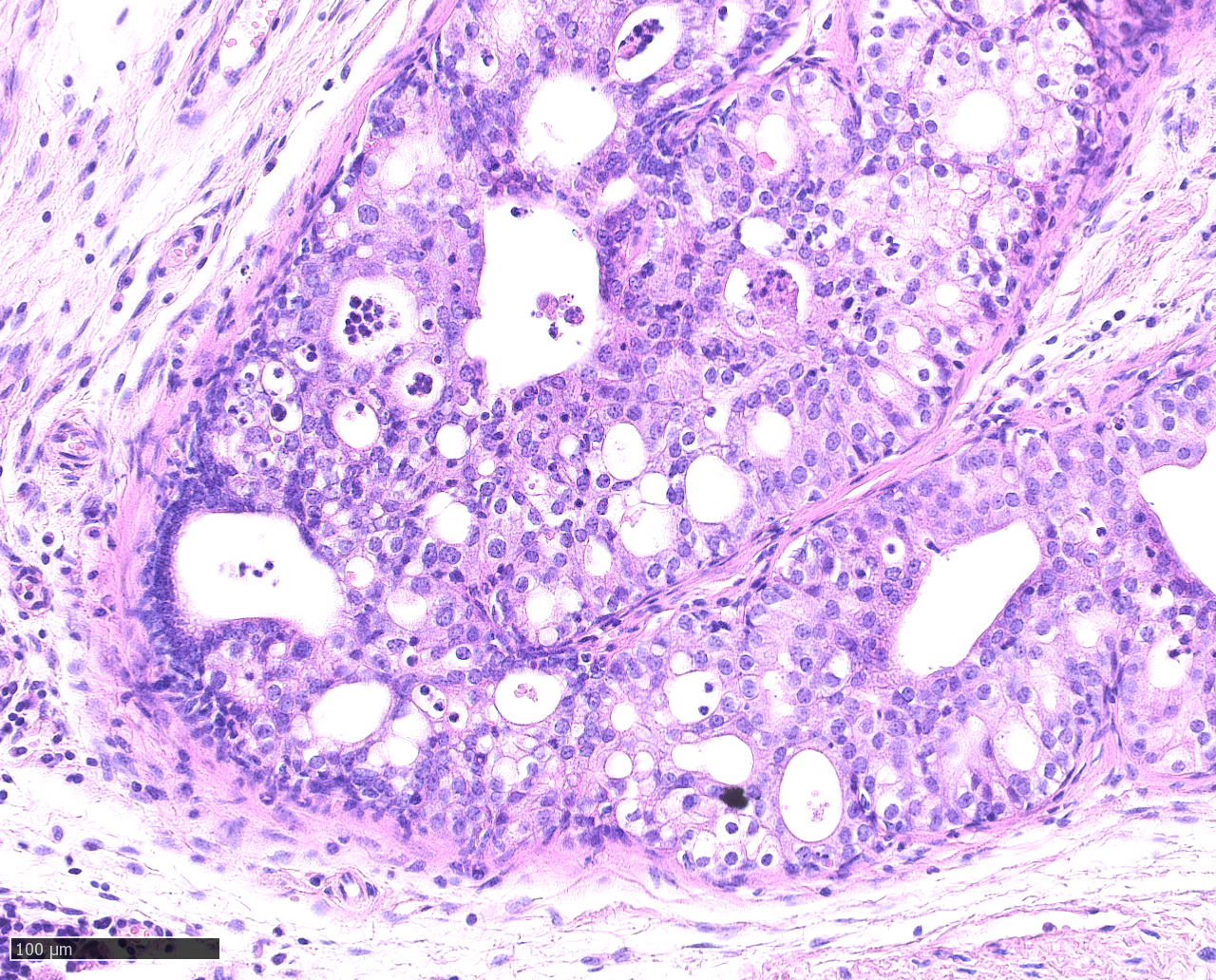

Supplement: Supplementary file 6 — Source Data for Figure 2 [file EMMM-15-e17209-s004.zip › Fig 2/2_F/PTENHIF_CTX_48h.tif]

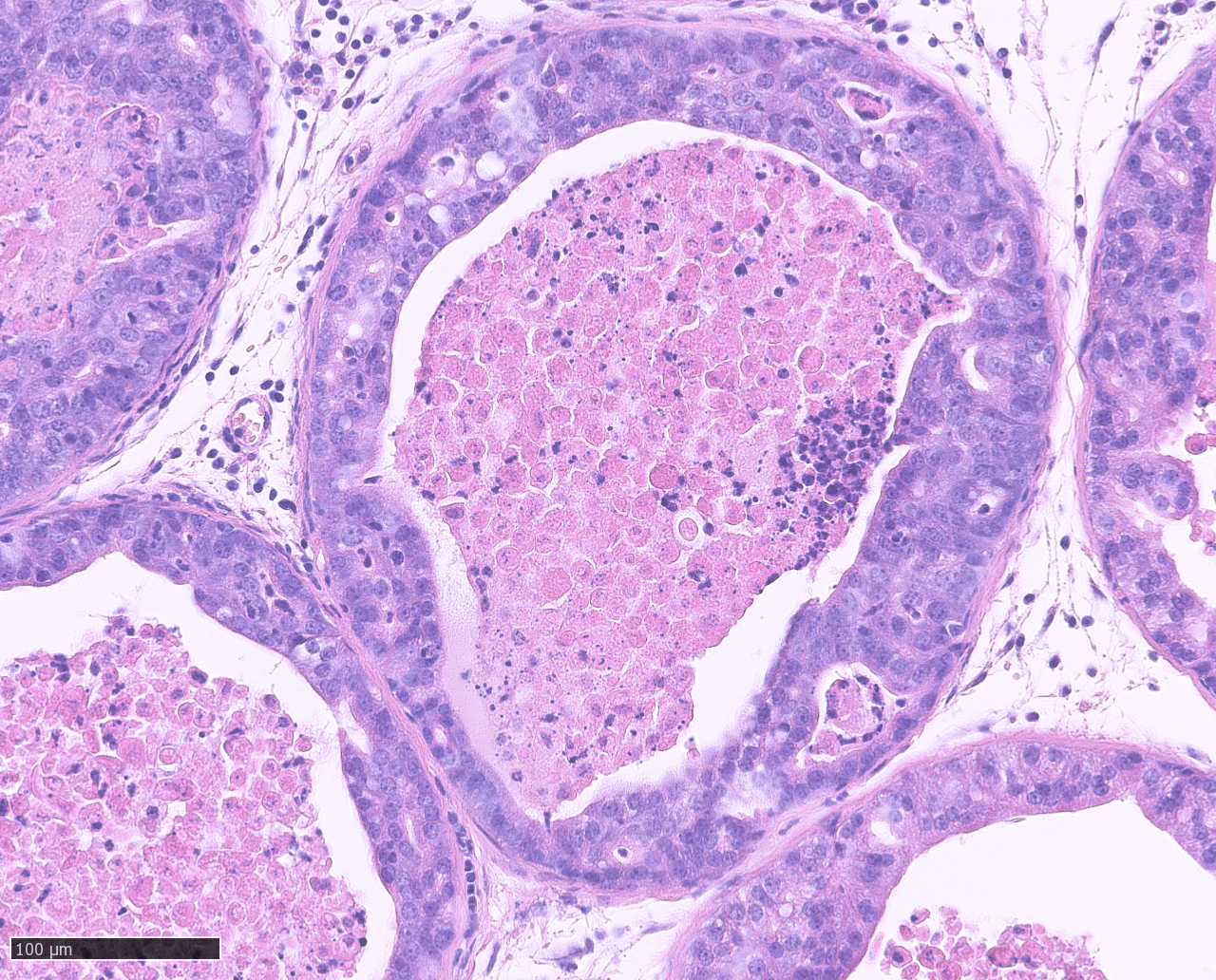

Supplement: Supplementary file 6 — Source Data for Figure 2 [file EMMM-15-e17209-s004.zip › Fig 2/2_F/PTENHIF_CTX_72h.tif]

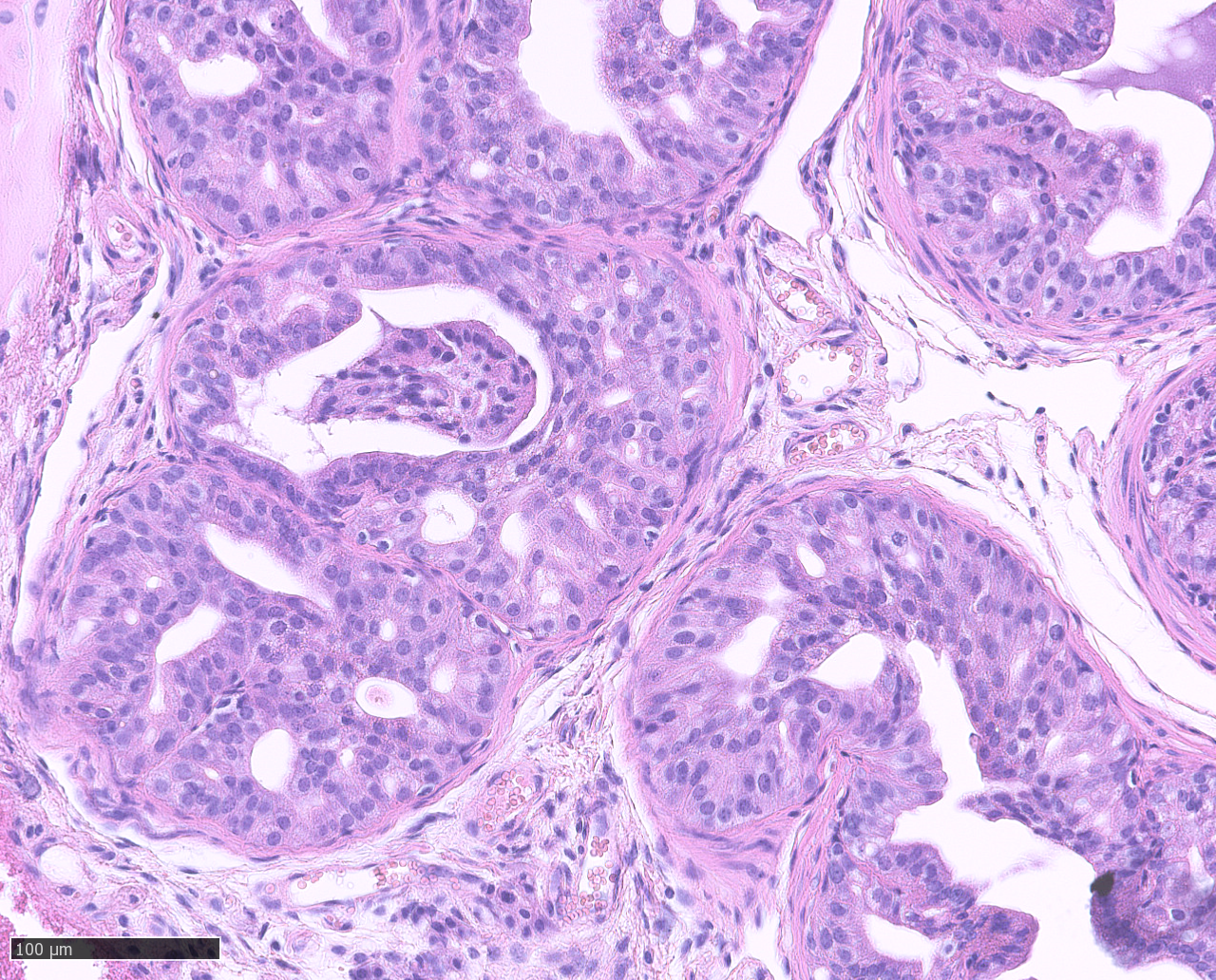

Supplement: Supplementary file 6 — Source Data for Figure 2 [file EMMM-15-e17209-s004.zip › Fig 2/2_F/PTENHIF_sham_24h.tif]

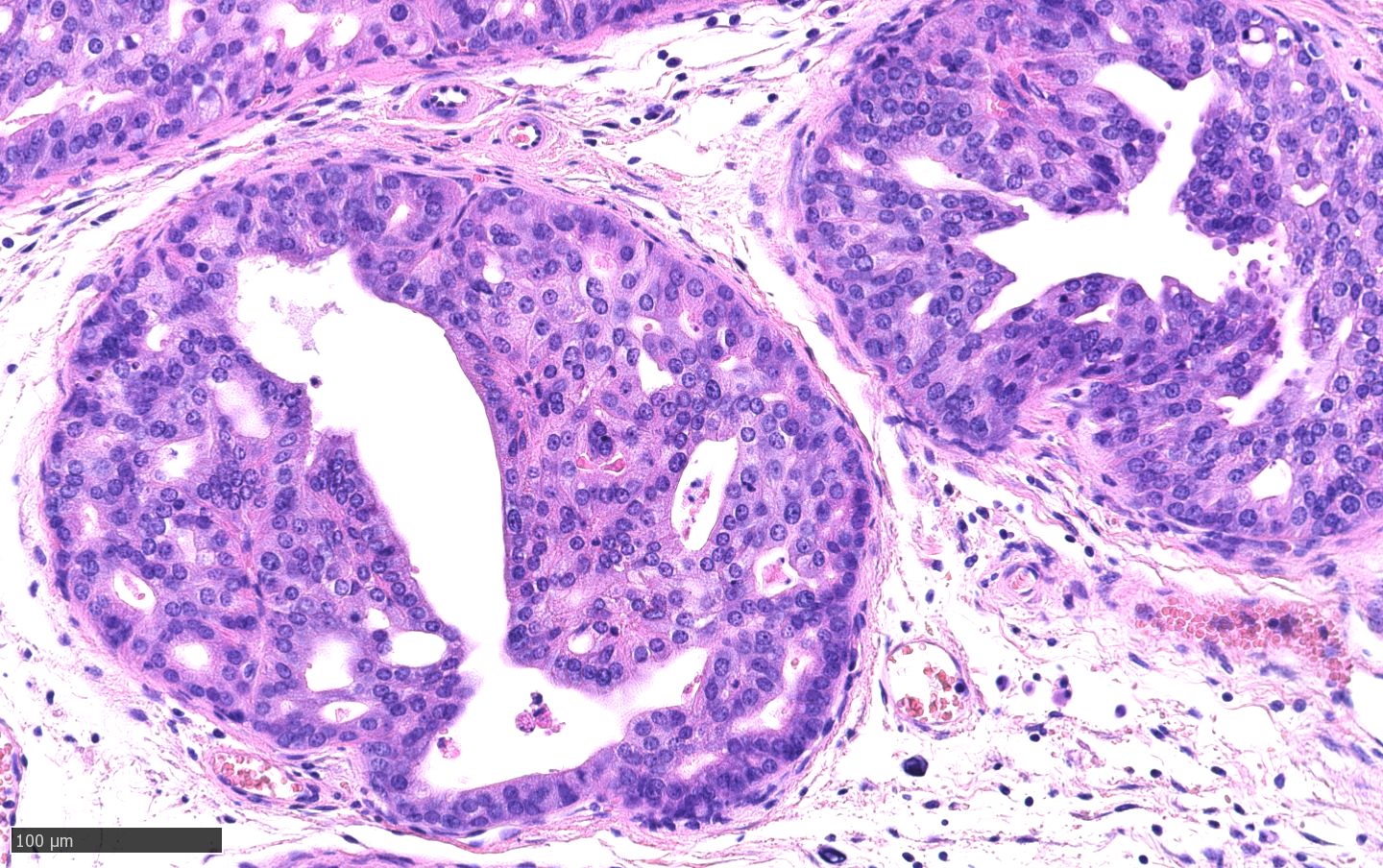

Supplement: Supplementary file 6 — Source Data for Figure 2 [file EMMM-15-e17209-s004.zip › Fig 2/2_F/PTEN_CTX_24h_HE.tif]

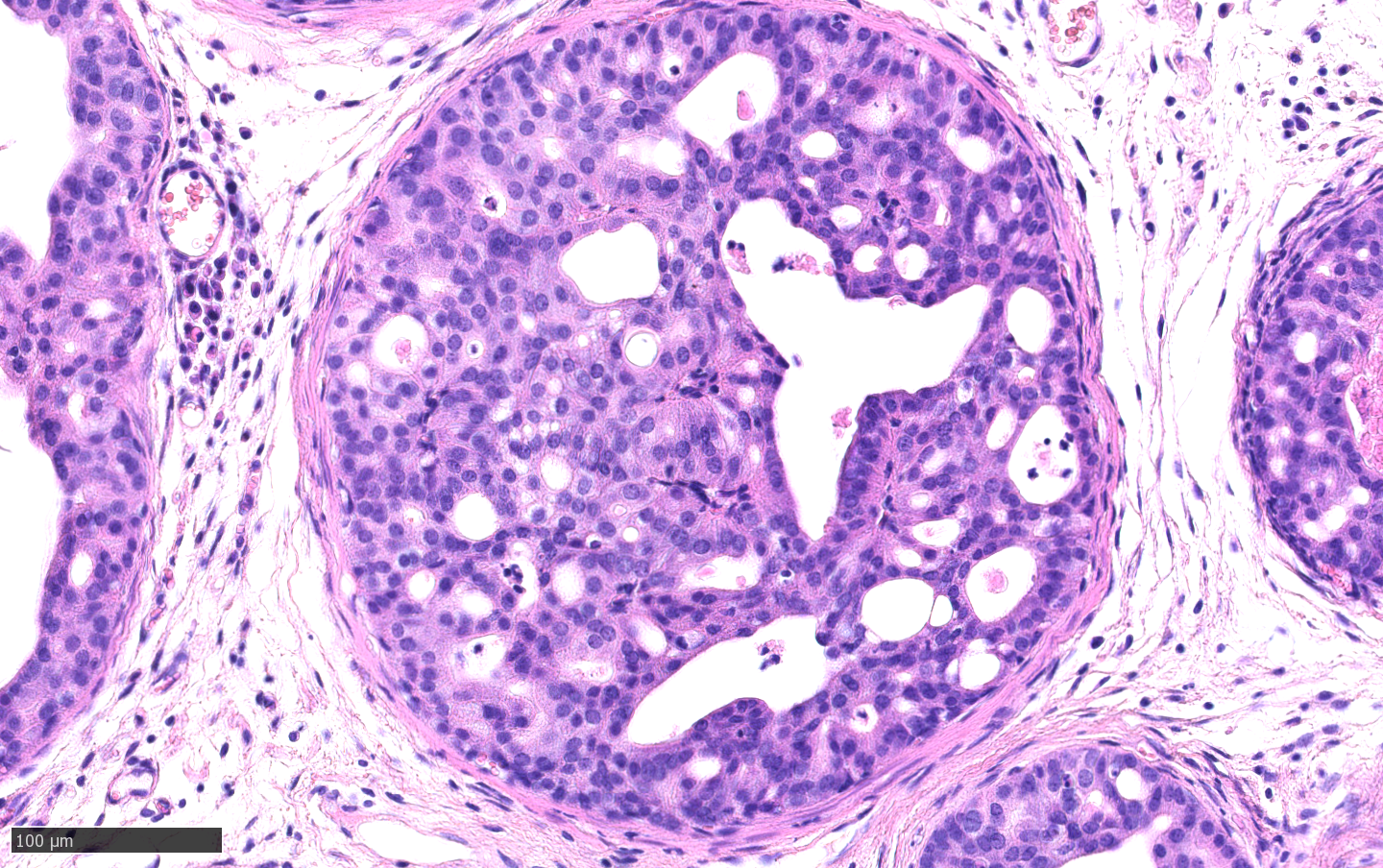

Supplement: Supplementary file 6 — Source Data for Figure 2 [file EMMM-15-e17209-s004.zip › Fig 2/2_F/PTEN_CTX_48h_HE.tif]

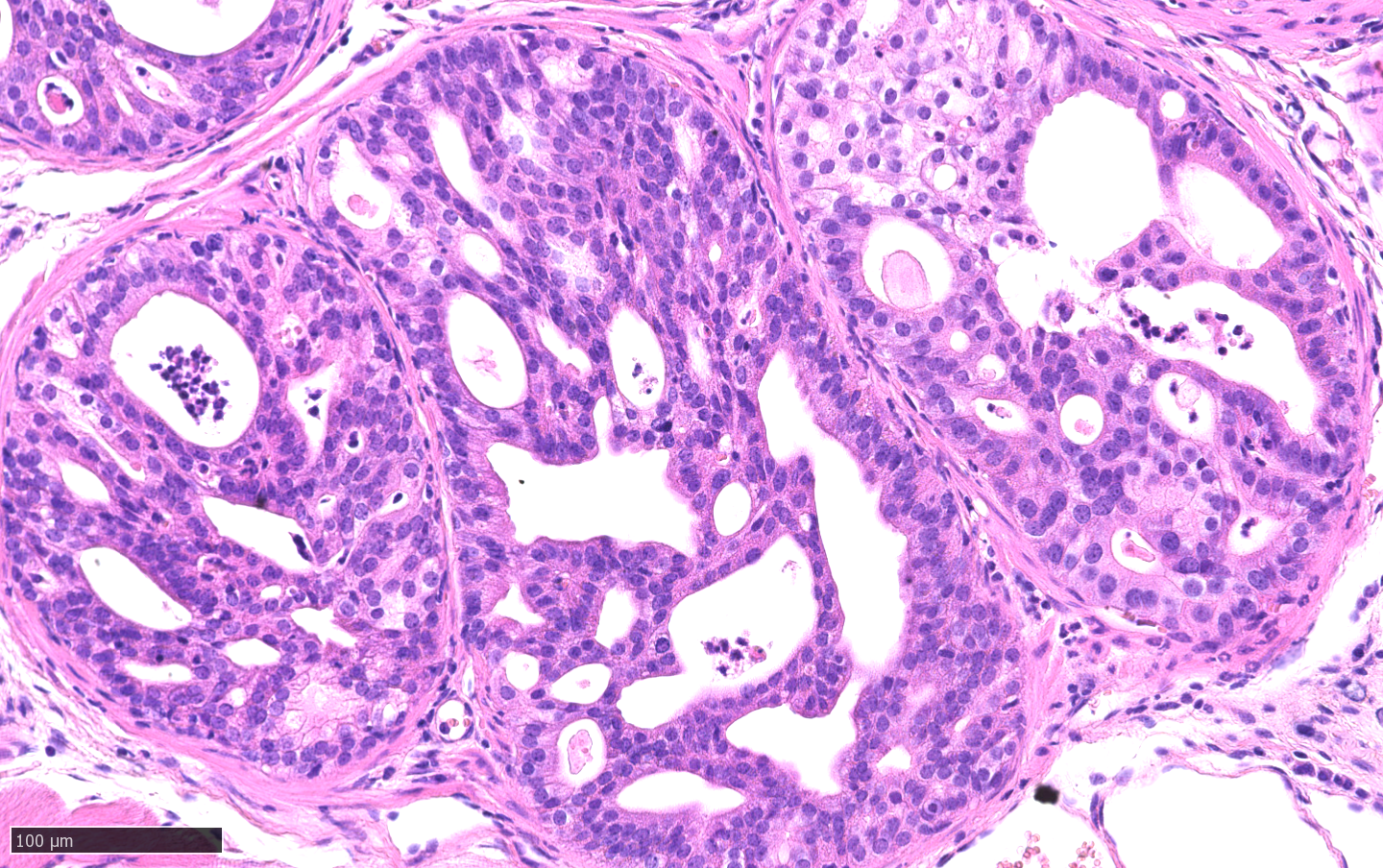

Supplement: Supplementary file 6 — Source Data for Figure 2 [file EMMM-15-e17209-s004.zip › Fig 2/2_F/PTEN_CTX_72h_HE.tif]

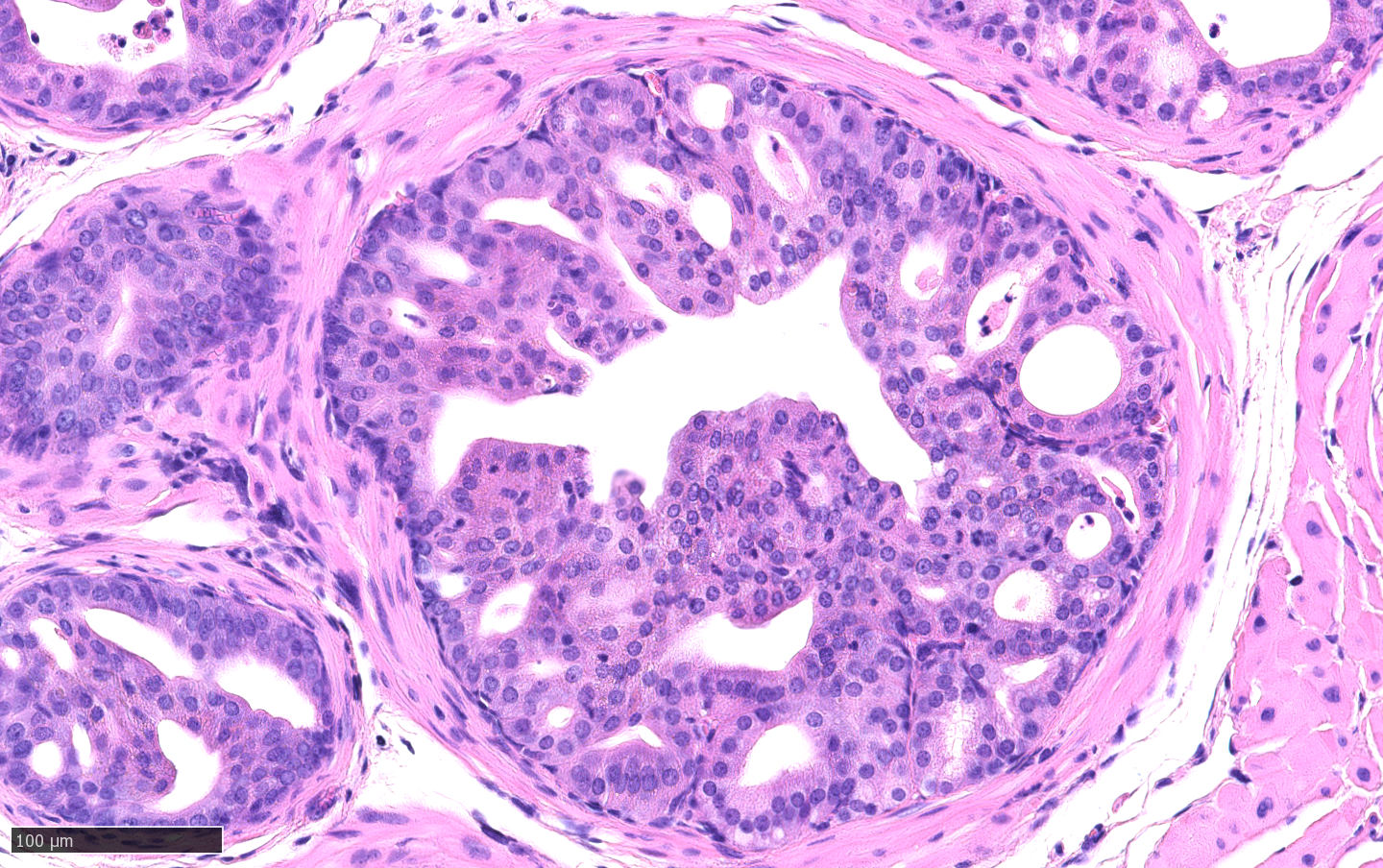

Supplement: Supplementary file 6 — Source Data for Figure 2 [file EMMM-15-e17209-s004.zip › Fig 2/2_F/PTEN_sham_24h_HE.tif]

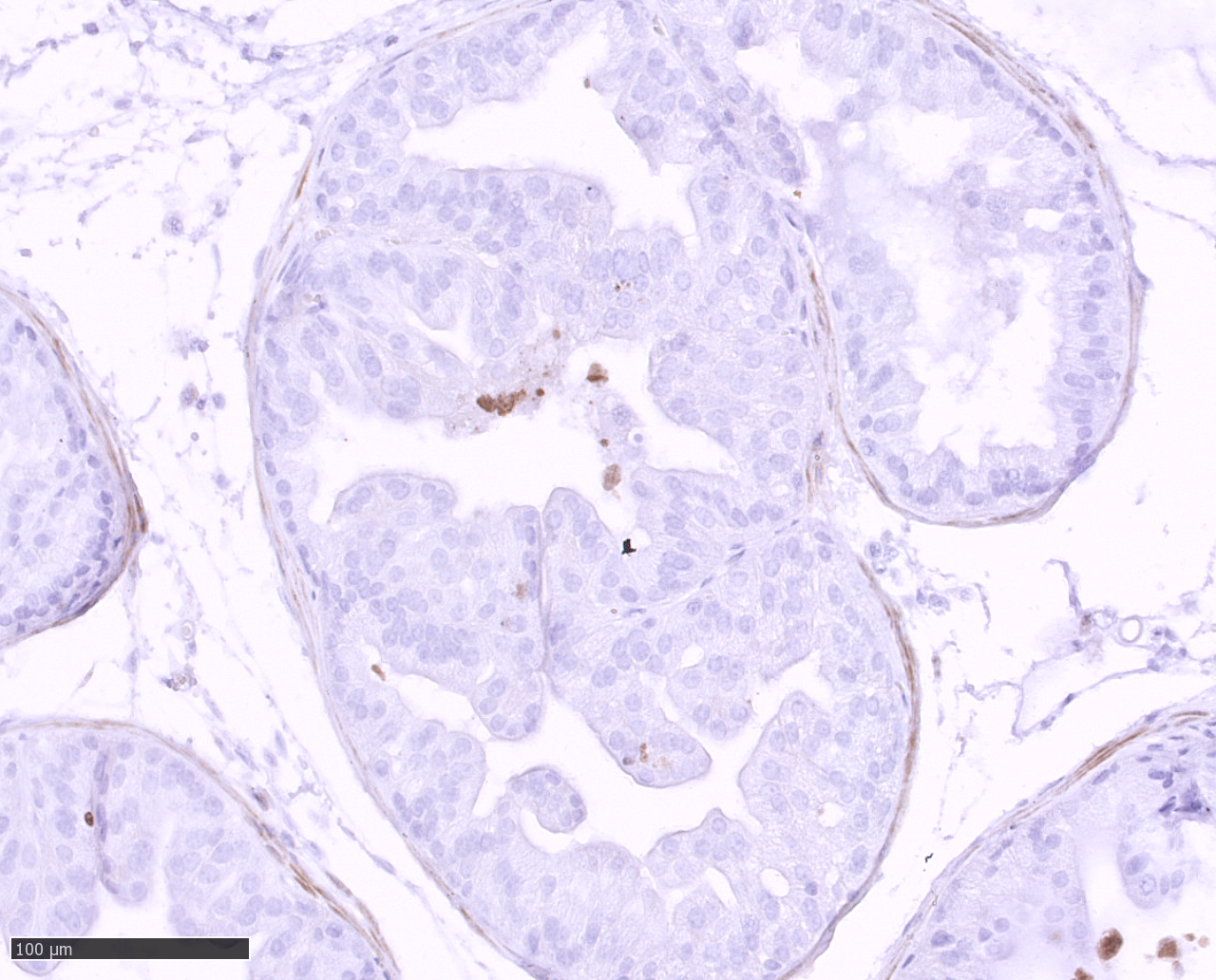

Supplement: Supplementary file 6 — Source Data for Figure 2 [file EMMM-15-e17209-s004.zip › Fig 2/2_G/ptenhif_CTX_1D_CC3.tif]

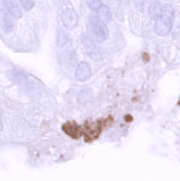

Supplement: Supplementary file 6 — Source Data for Figure 2 [file EMMM-15-e17209-s004.zip › Fig 2/2_G/ptenhif_CTX_1D_CC3_zoom.tif]

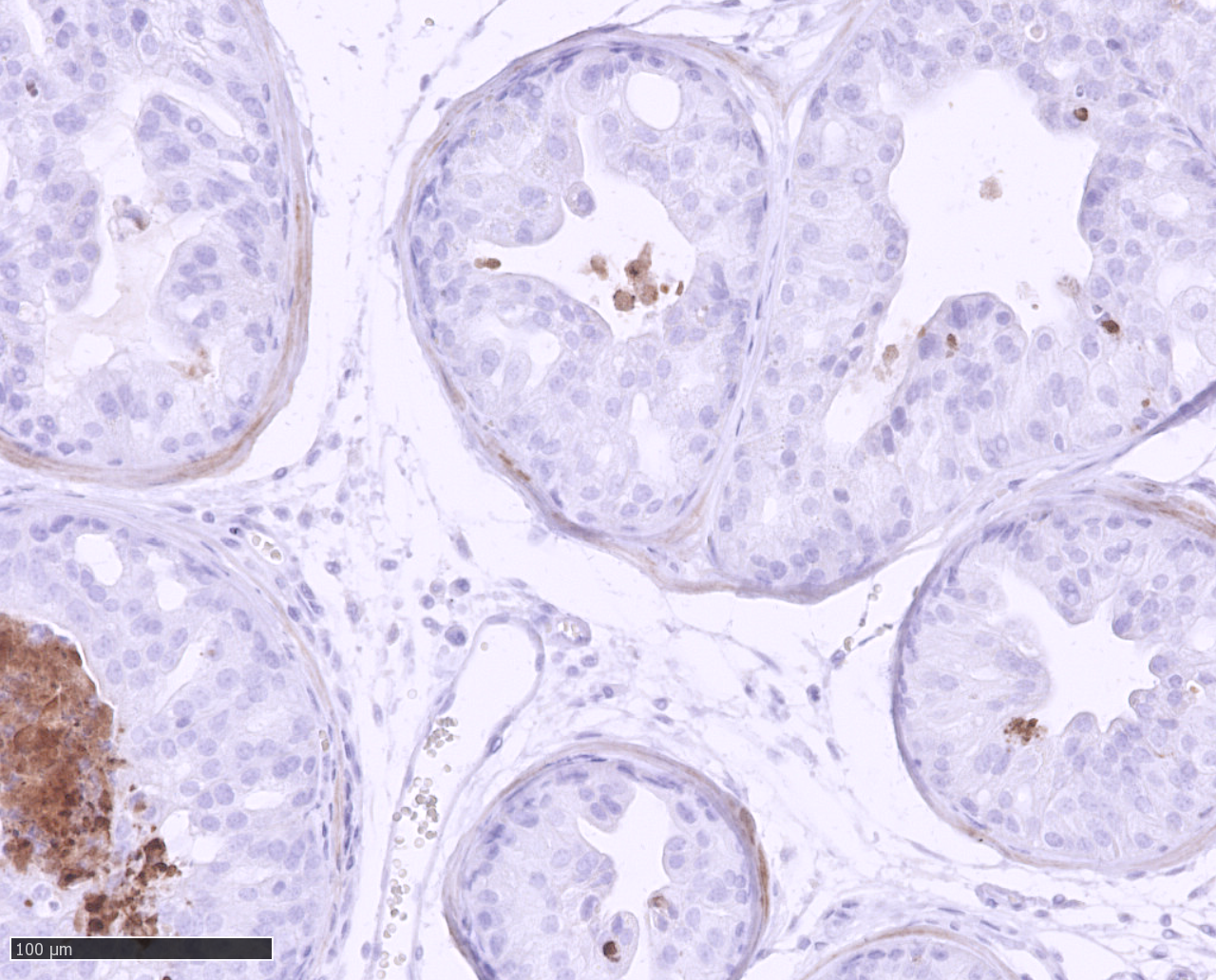

Supplement: Supplementary file 6 — Source Data for Figure 2 [file EMMM-15-e17209-s004.zip › Fig 2/2_G/ptenhif_CTX_2D_CC3.tif]

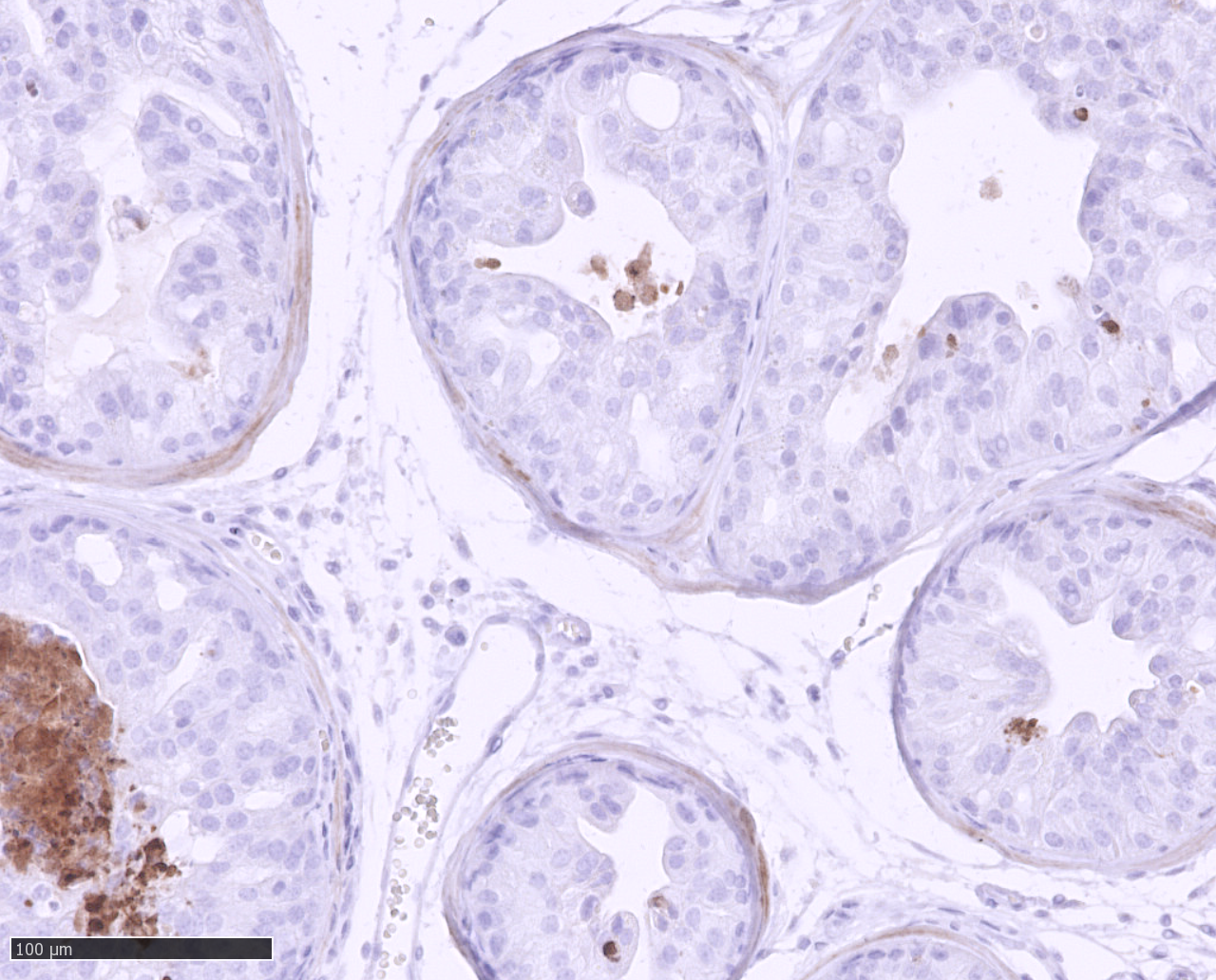

Supplement: Supplementary file 6 — Source Data for Figure 2 [file EMMM-15-e17209-s004.zip › Fig 2/2_G/ptenhif_CTX_2D_CC3_zoom.tif]

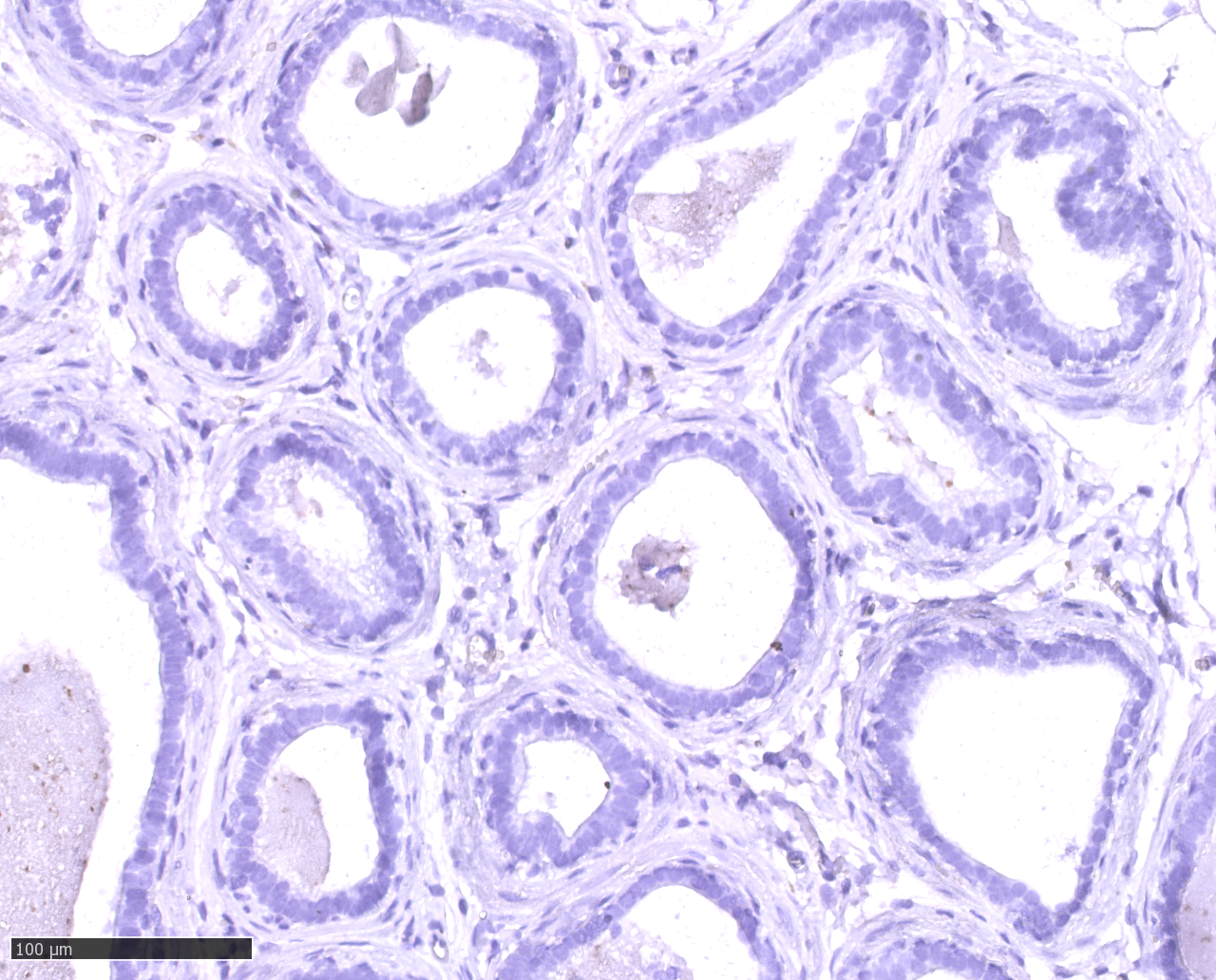

Supplement: Supplementary file 6 — Source Data for Figure 2 [file EMMM-15-e17209-s004.zip › Fig 2/2_G/ptenhif_CTX_30D_CC3.tif]

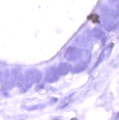

Supplement: Supplementary file 6 — Source Data for Figure 2 [file EMMM-15-e17209-s004.zip › Fig 2/2_G/ptenhif_CTX_30D_CC3zoom.tif]

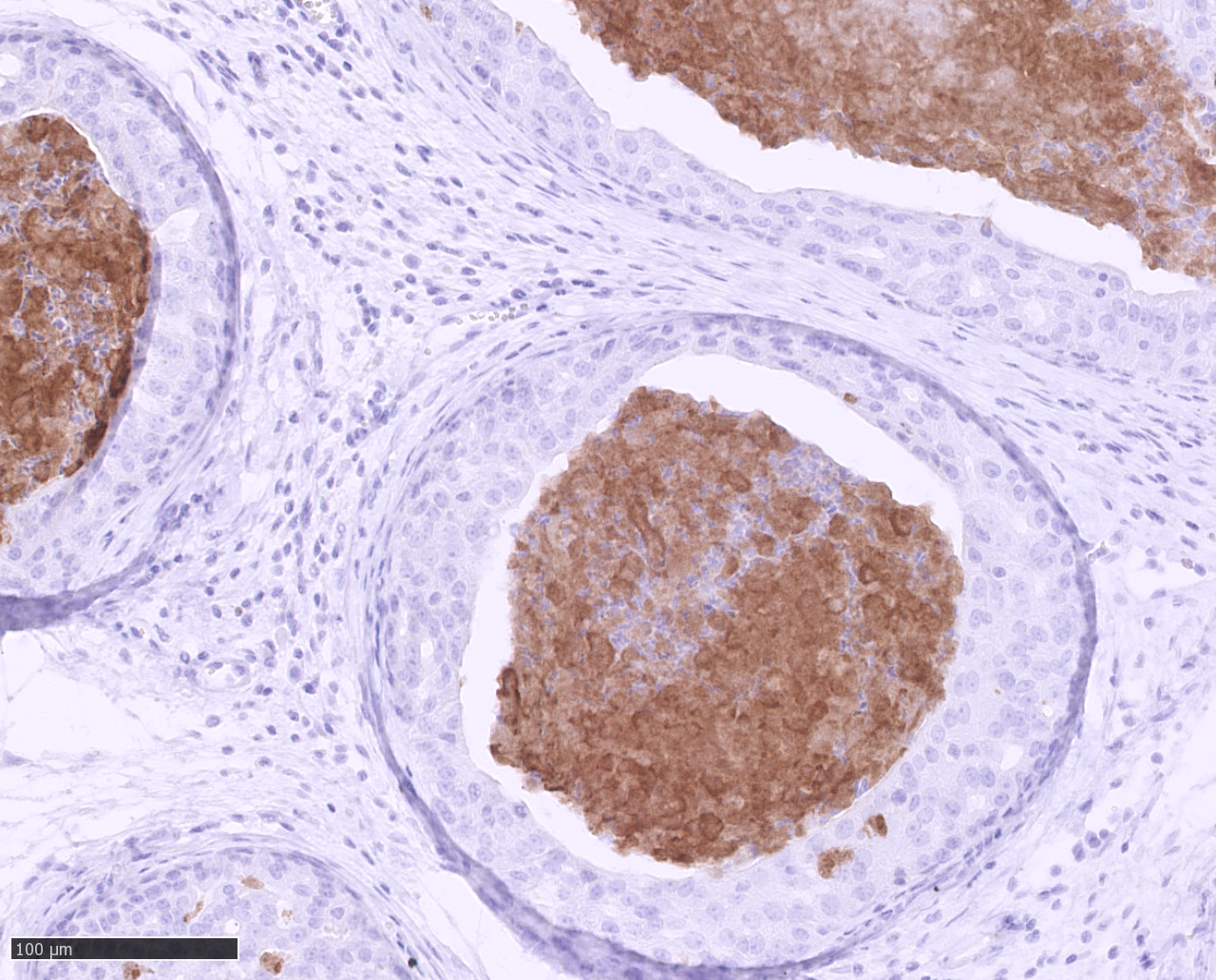

Supplement: Supplementary file 6 — Source Data for Figure 2 [file EMMM-15-e17209-s004.zip › Fig 2/2_G/ptenhif_CTX_3D_CC3.tif]

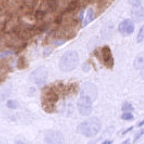

Supplement: Supplementary file 6 — Source Data for Figure 2 [file EMMM-15-e17209-s004.zip › Fig 2/2_G/ptenhif_CTX_3D_CC3_zoomtif.tif]

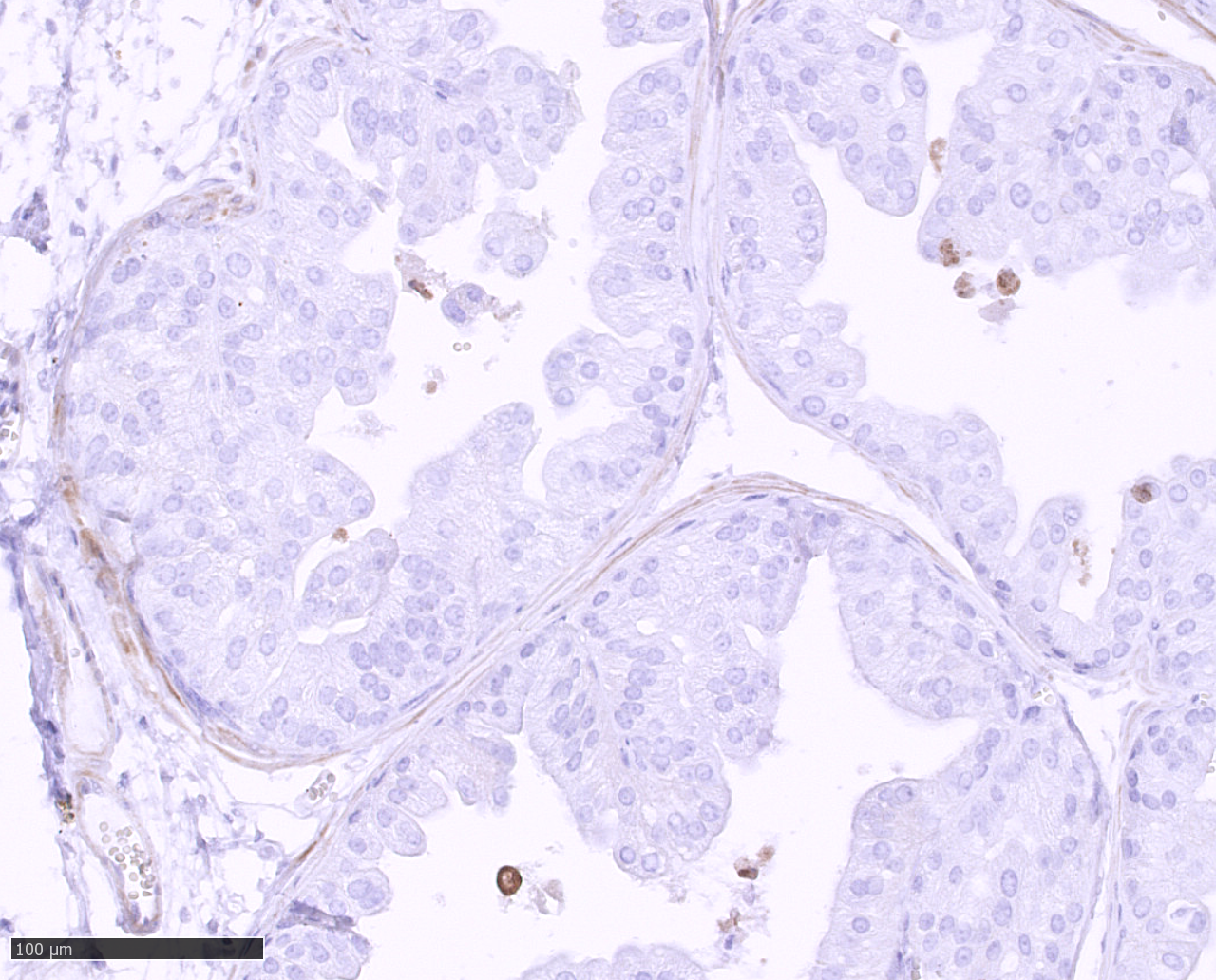

Supplement: Supplementary file 6 — Source Data for Figure 2 [file EMMM-15-e17209-s004.zip › Fig 2/2_G/ptenhif_SHAM_1D_CC3.tif]

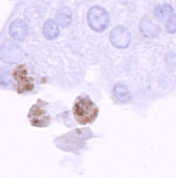

Supplement: Supplementary file 6 — Source Data for Figure 2 [file EMMM-15-e17209-s004.zip › Fig 2/2_G/ptenhif_SHAM_1D_CC3_zoom.tif]

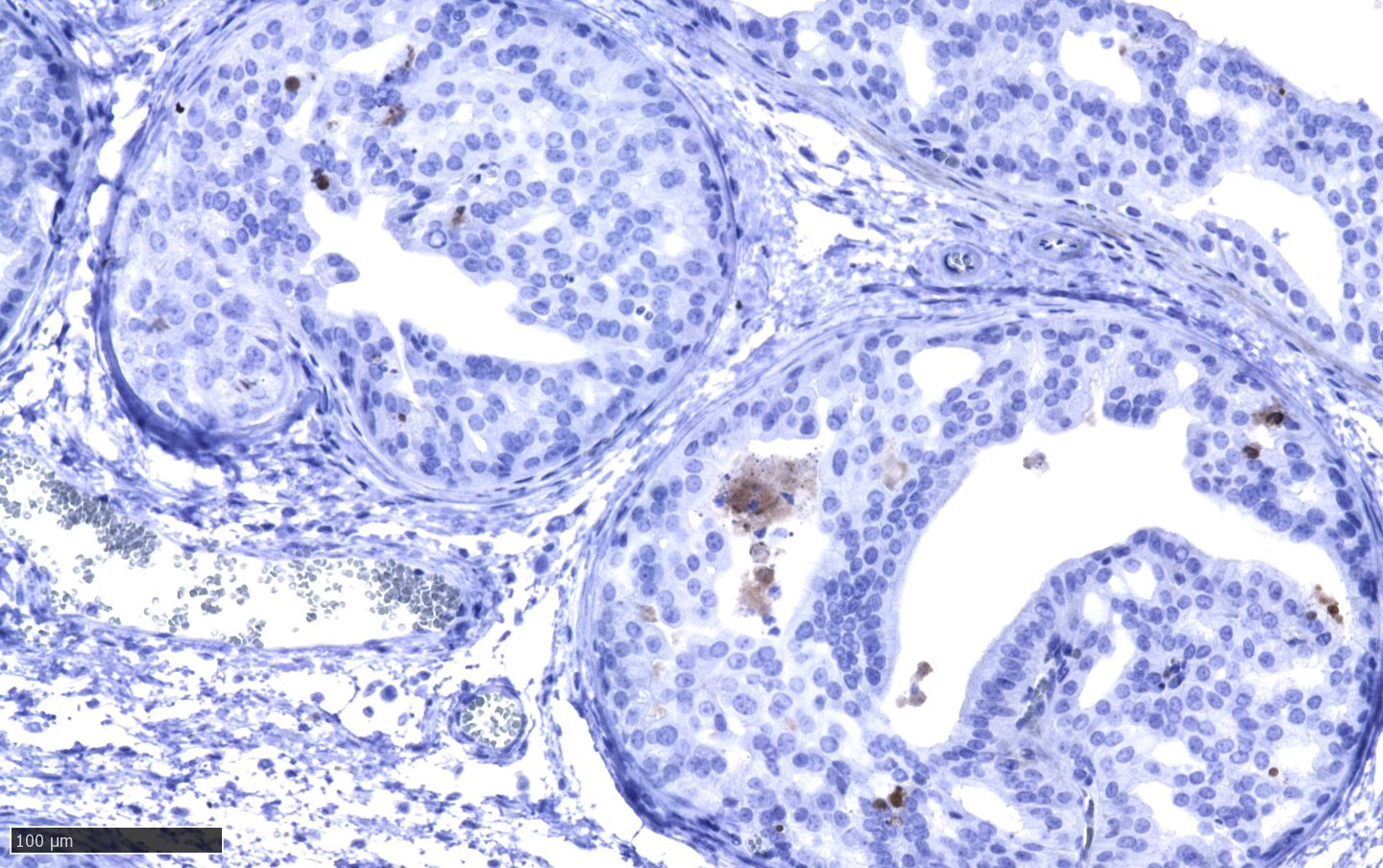

Supplement: Supplementary file 6 — Source Data for Figure 2 [file EMMM-15-e17209-s004.zip › Fig 2/2_G/PTEN_CTX_1D_CC3.tif]

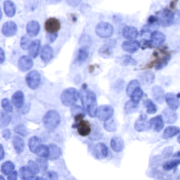

Supplement: Supplementary file 6 — Source Data for Figure 2 [file EMMM-15-e17209-s004.zip › Fig 2/2_G/PTEN_CTX_1D_zoomCC3.tif]

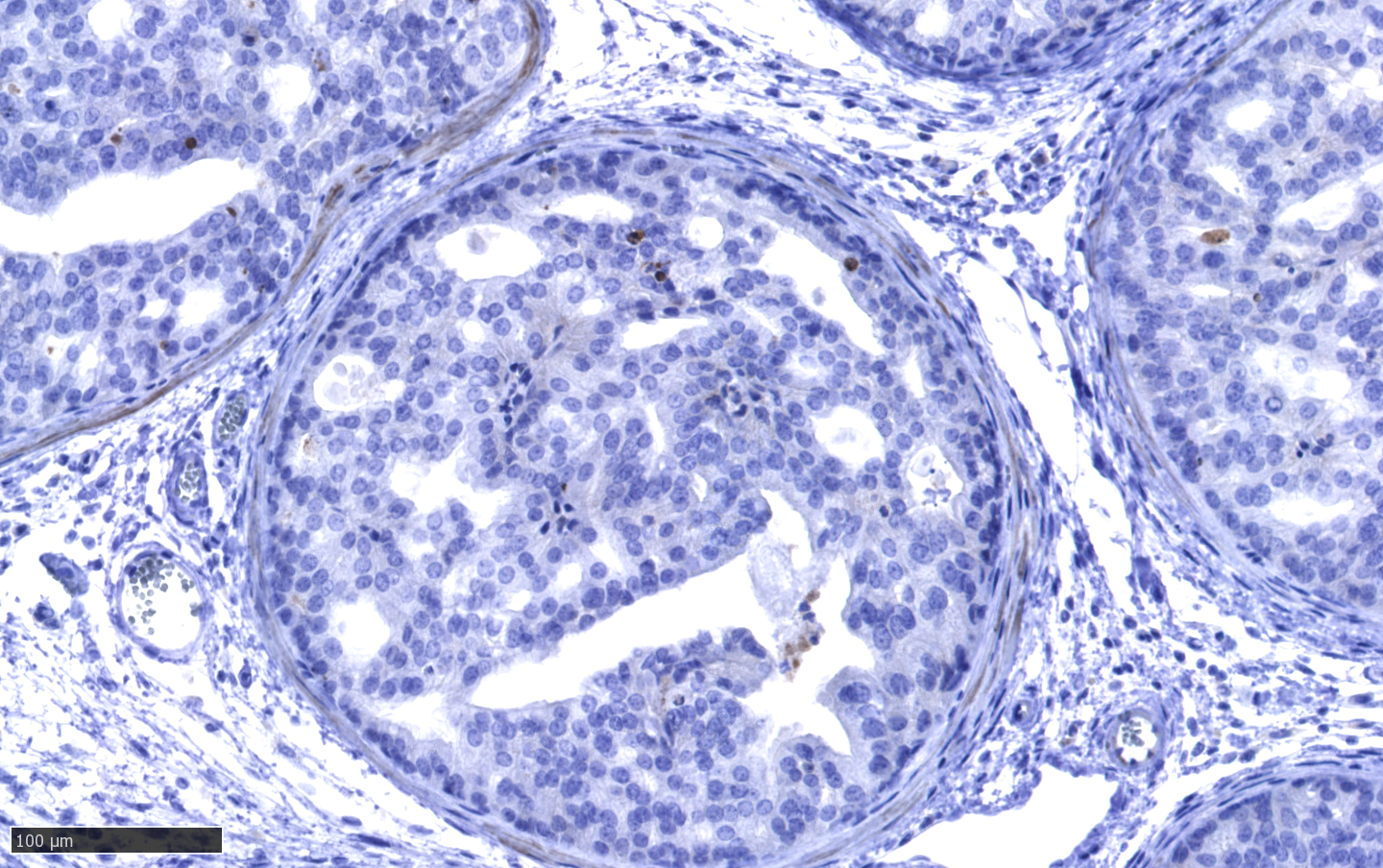

Supplement: Supplementary file 6 — Source Data for Figure 2 [file EMMM-15-e17209-s004.zip › Fig 2/2_G/PTEN_CTX_2D_CC3.tif]

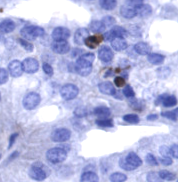

Supplement: Supplementary file 6 — Source Data for Figure 2 [file EMMM-15-e17209-s004.zip › Fig 2/2_G/PTEN_CTX_2D_CC3_zoom.tif]

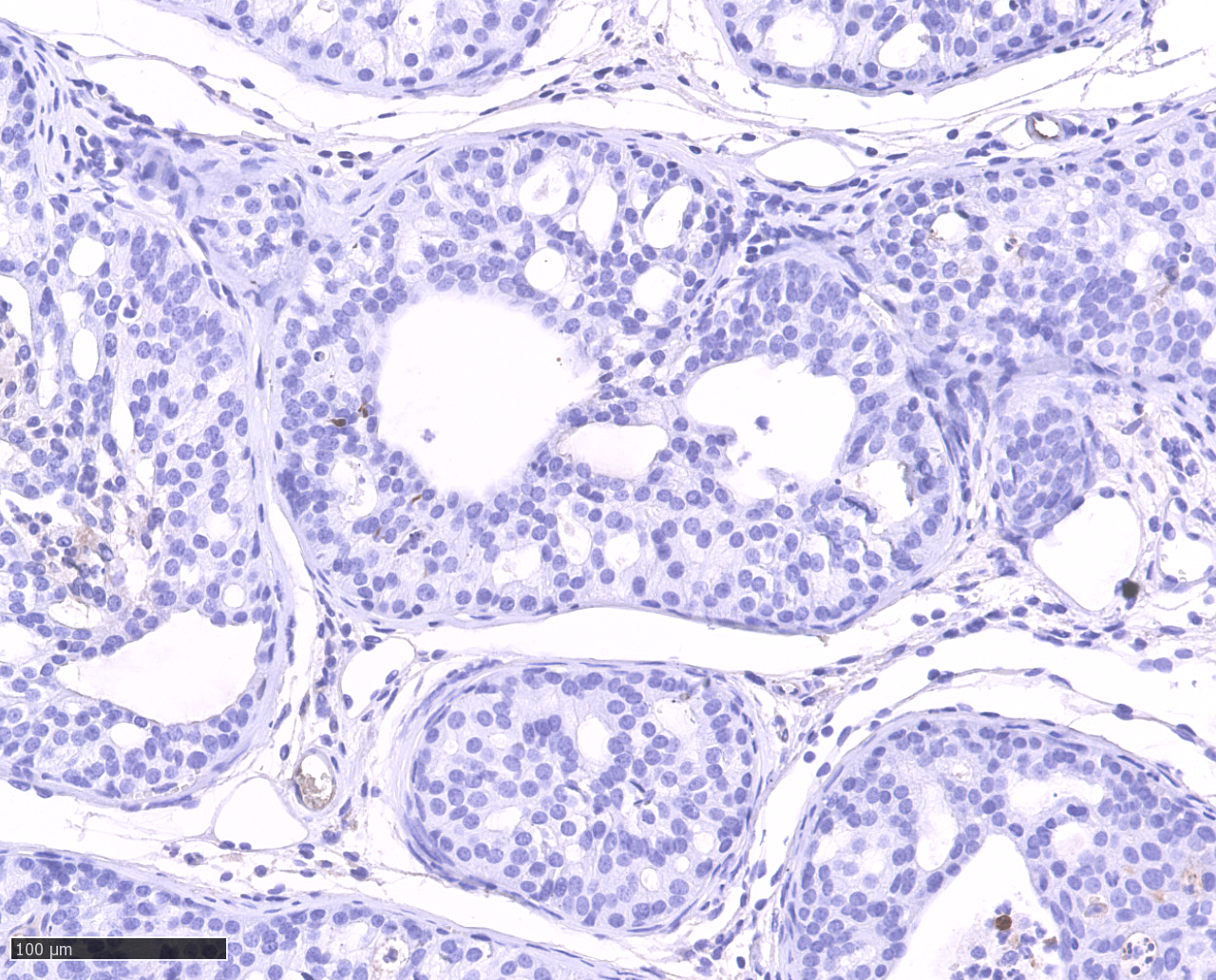

Supplement: Supplementary file 6 — Source Data for Figure 2 [file EMMM-15-e17209-s004.zip › Fig 2/2_G/PTEN_CTX_30D_CC3.tif]

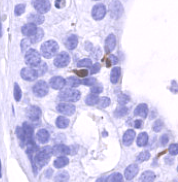

Supplement: Supplementary file 6 — Source Data for Figure 2 [file EMMM-15-e17209-s004.zip › Fig 2/2_G/PTEN_CTX_30D_CC3_zoom.tif]

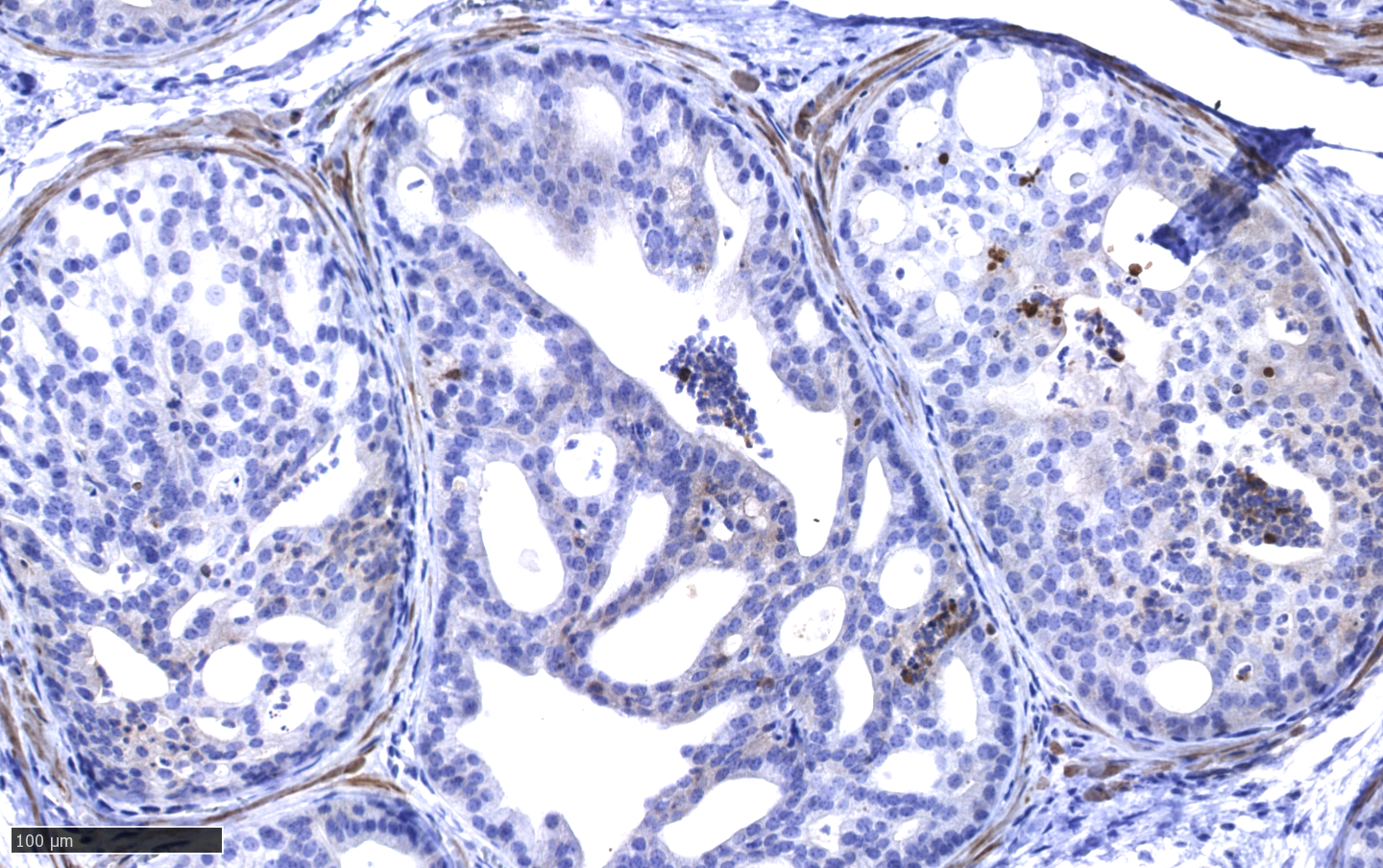

Supplement: Supplementary file 6 — Source Data for Figure 2 [file EMMM-15-e17209-s004.zip › Fig 2/2_G/PTEN_CTX_3D_CC3.tif]

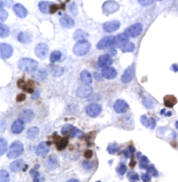

Supplement: Supplementary file 6 — Source Data for Figure 2 [file EMMM-15-e17209-s004.zip › Fig 2/2_G/PTEN_CTX_3D_CC3_zoom.tif]

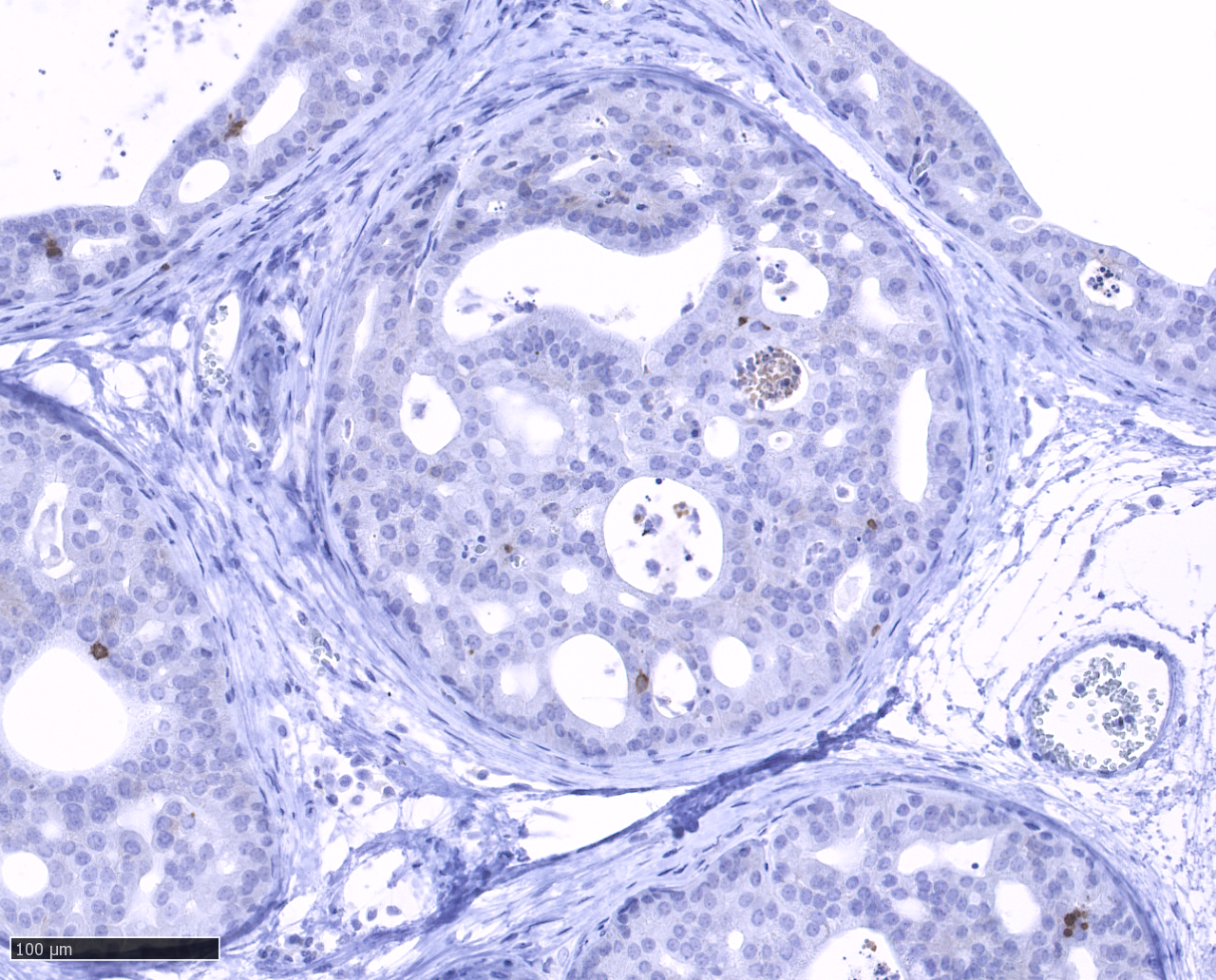

Supplement: Supplementary file 6 — Source Data for Figure 2 [file EMMM-15-e17209-s004.zip › Fig 2/2_G/PTEN_sham_1D_CC3.tif]

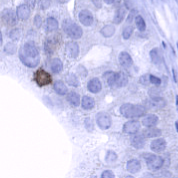

Supplement: Supplementary file 6 — Source Data for Figure 2 [file EMMM-15-e17209-s004.zip › Fig 2/2_G/PTEN_sham_1D_CC3_zoom.tif]

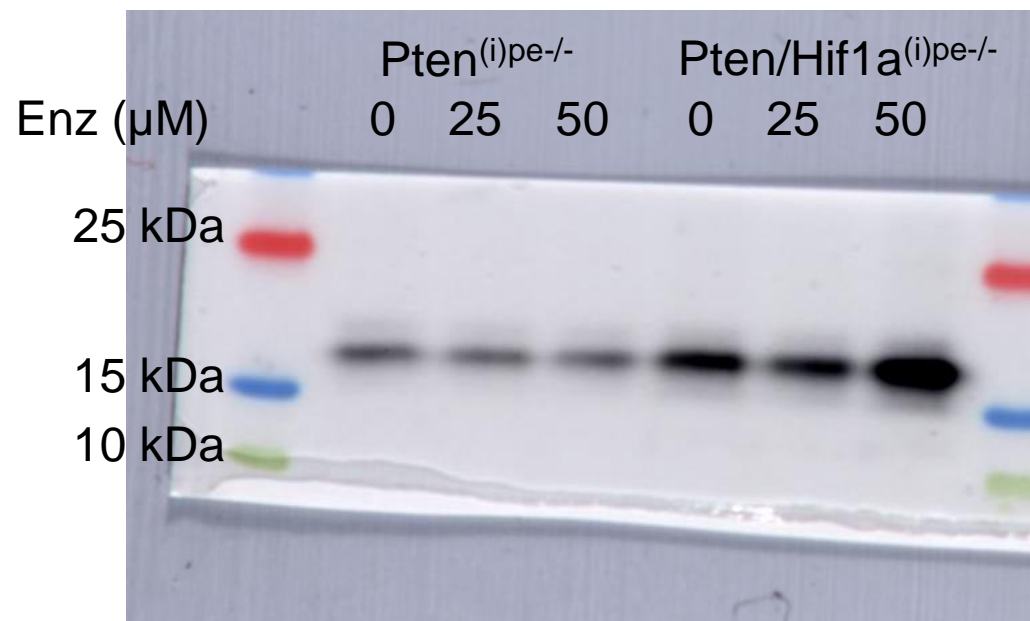

Cleaved caspase 3

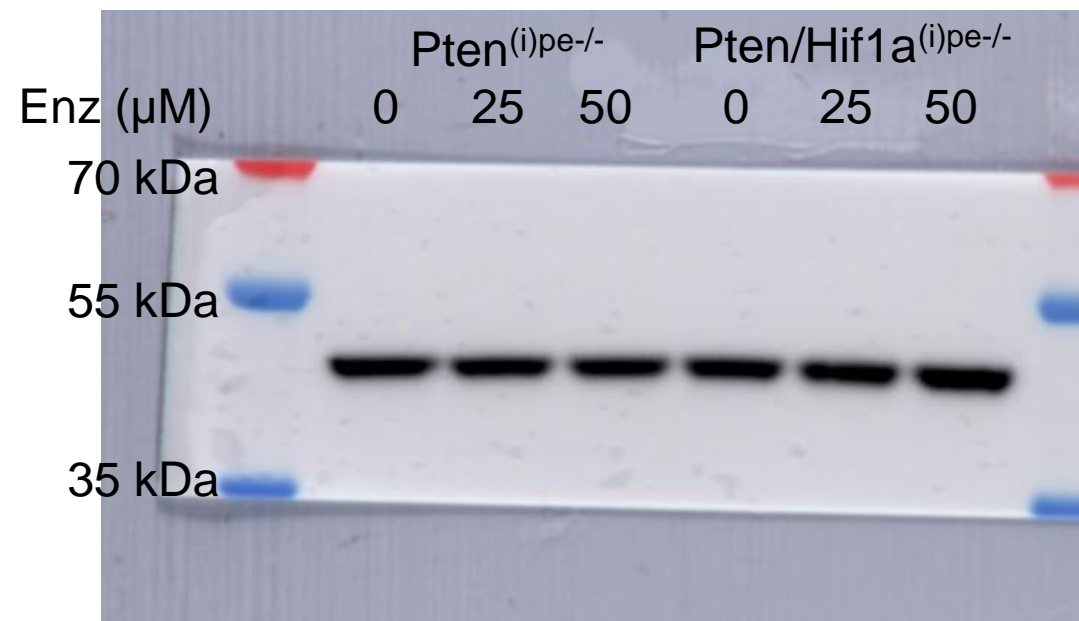

Beta-actin

Supplement: Supplementary file 6 — Source Data for Figure 2 [file EMMM-15-e17209-s004.zip › Fig 2/2_I/Fig 2I_source data.pdf]

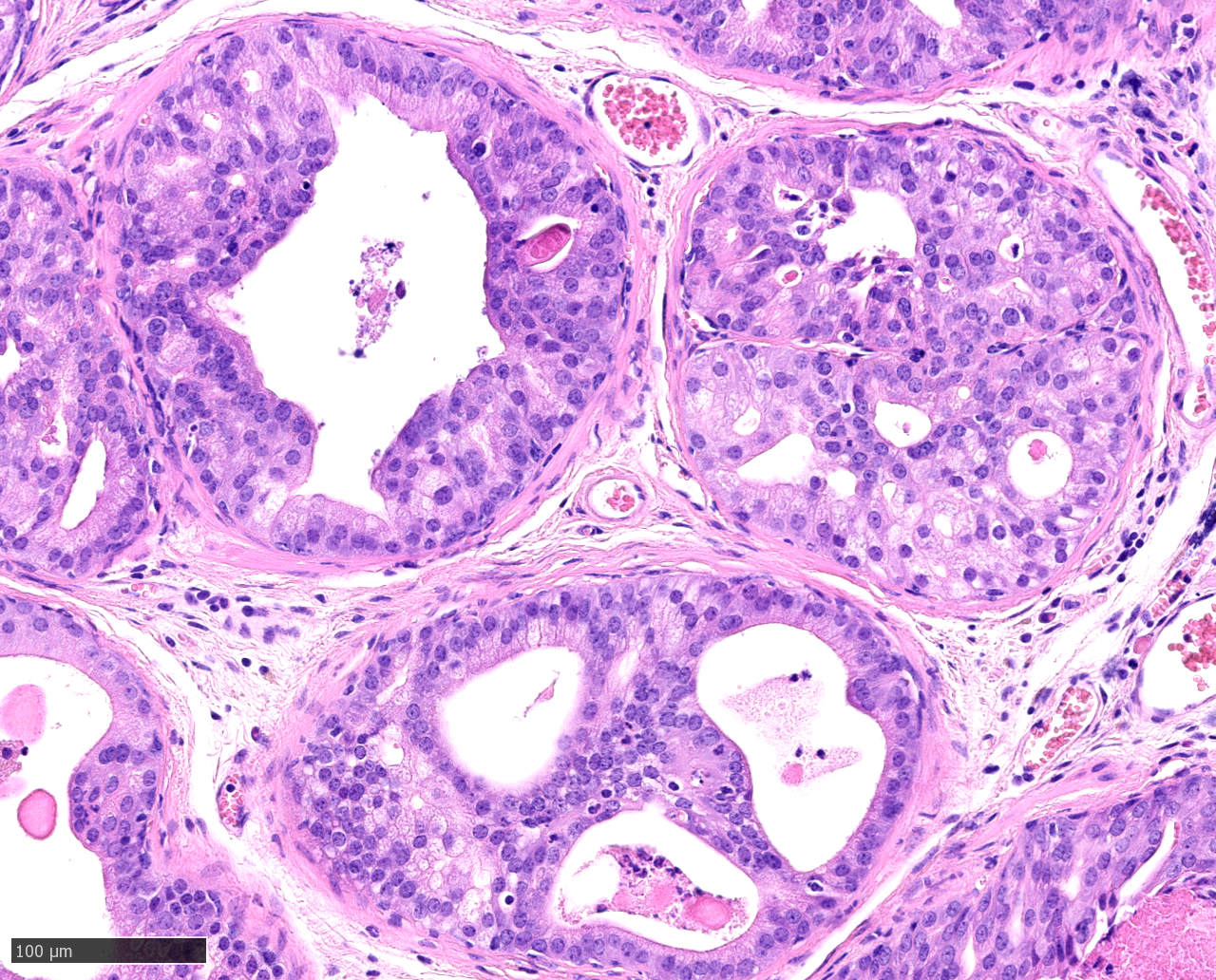

Supplement: Supplementary file 7 — Source Data for Figure 3 [file EMMM-15-e17209-s008.zip › Fig 3/3_B/HE_PTENHIF_3S5.tif]

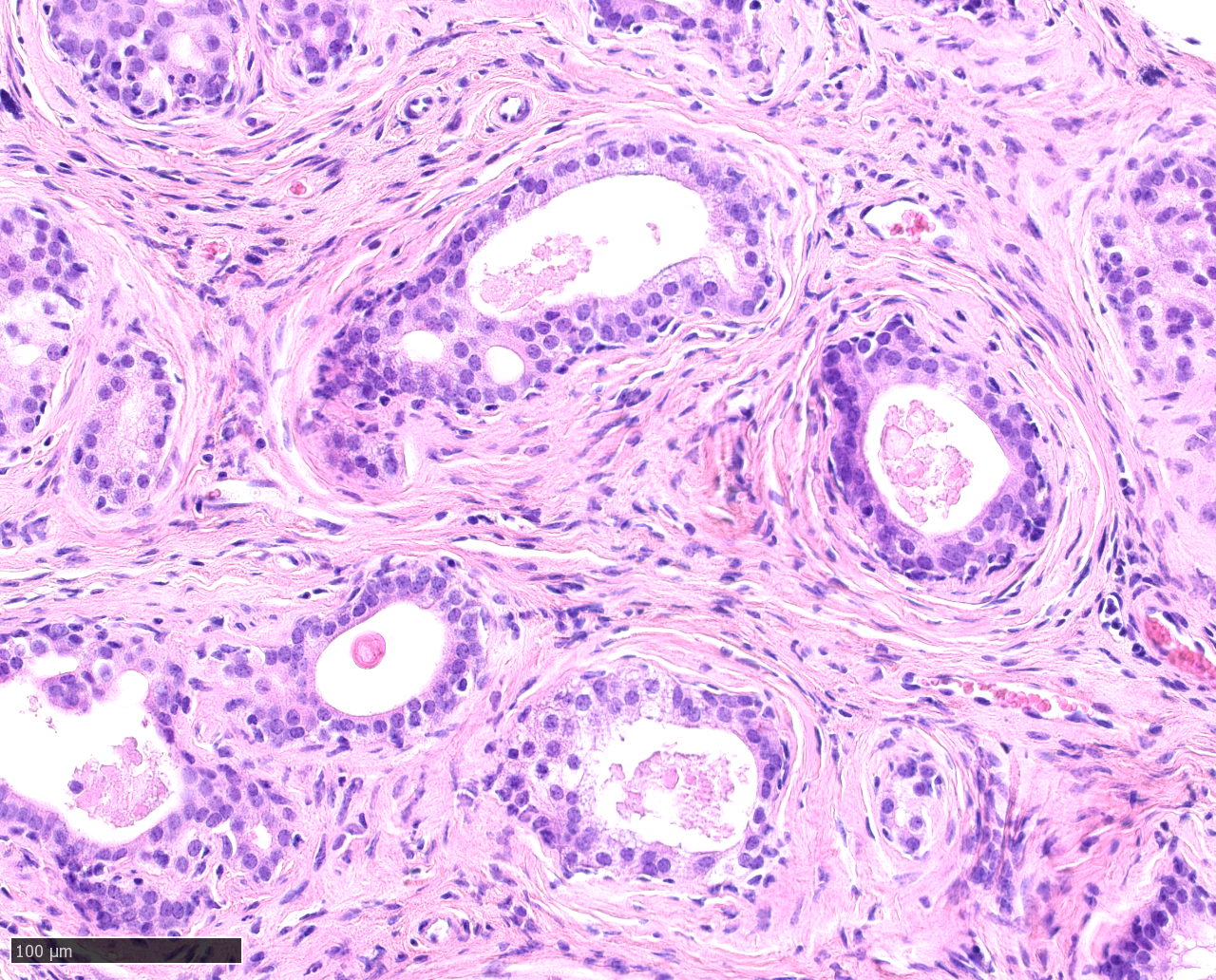

Supplement: Supplementary file 7 — Source Data for Figure 3 [file EMMM-15-e17209-s008.zip › Fig 3/3_B/PTENHIF_3C5_HE.tif]

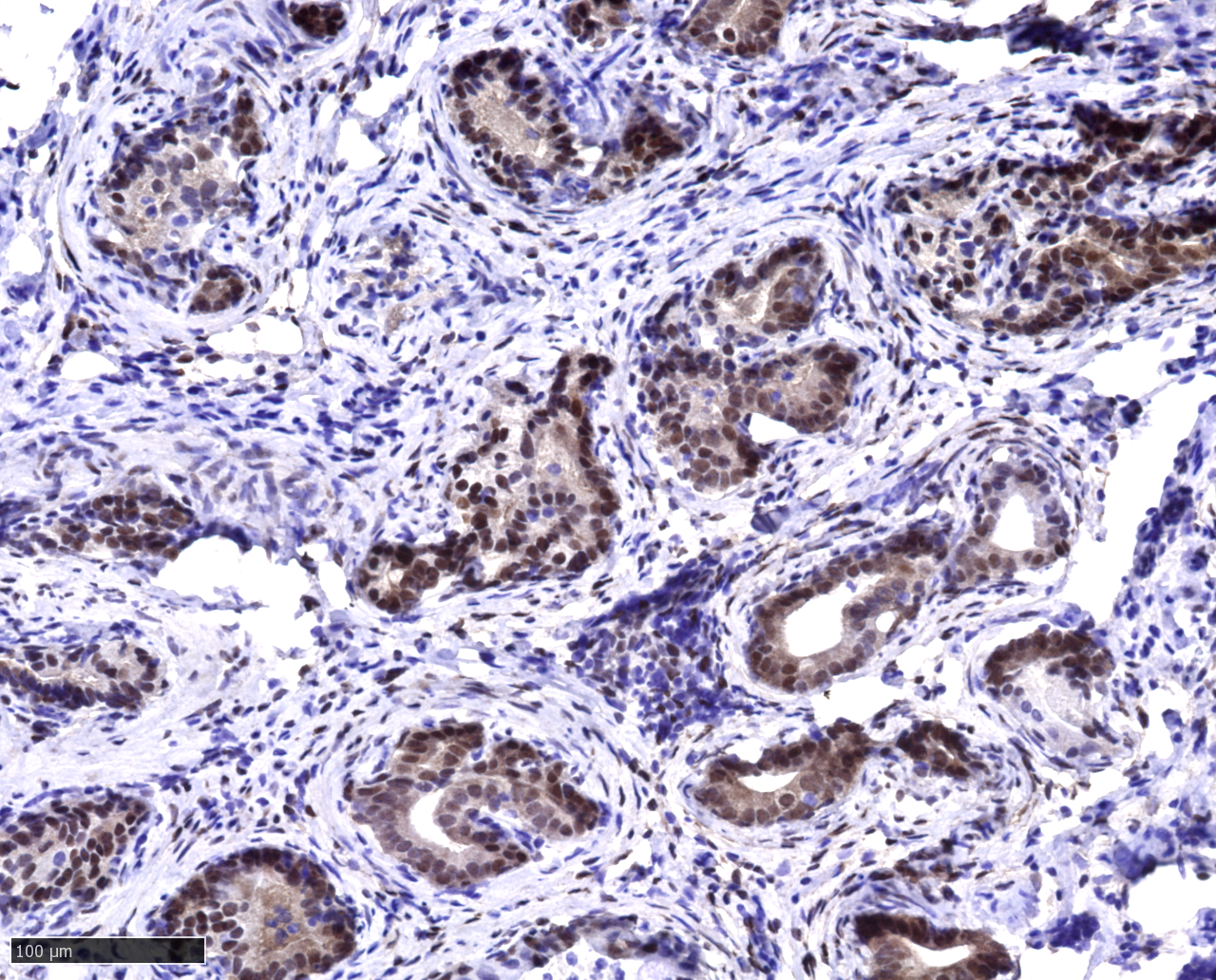

Supplement: Supplementary file 7 — Source Data for Figure 3 [file EMMM-15-e17209-s008.zip › Fig 3/3_E/PTENHIF_3C5_ar.tif]

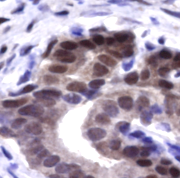

Supplement: Supplementary file 7 — Source Data for Figure 3 [file EMMM-15-e17209-s008.zip › Fig 3/3_E/PTENHIF_3C5_ar_zoom.tif]

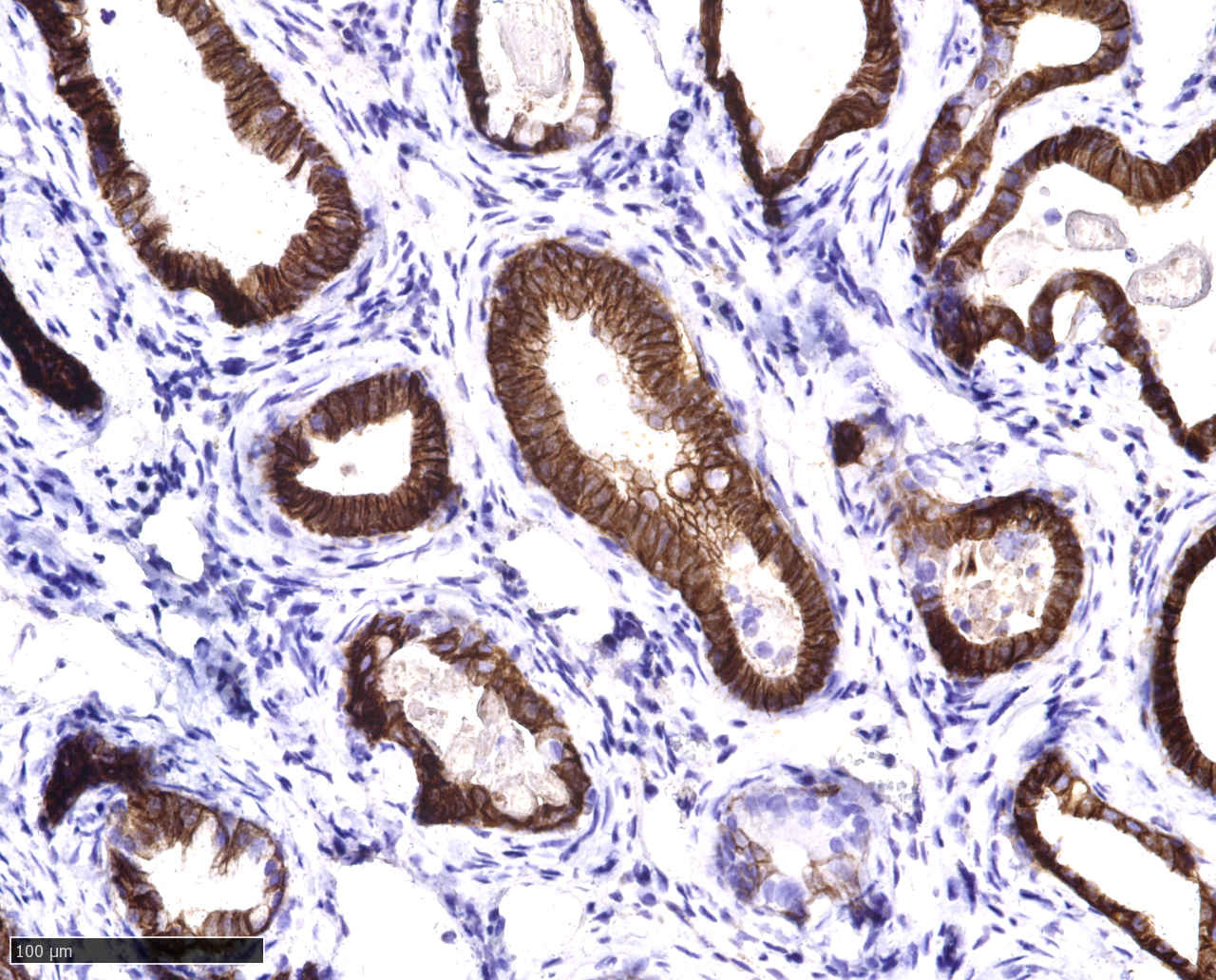

Supplement: Supplementary file 7 — Source Data for Figure 3 [file EMMM-15-e17209-s008.zip › Fig 3/3_E/PTENHIF_3C5_trop2.tif]

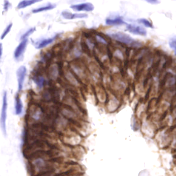

Supplement: Supplementary file 7 — Source Data for Figure 3 [file EMMM-15-e17209-s008.zip › Fig 3/3_E/PTENHIF_3C5_Trop2zoom.tif]

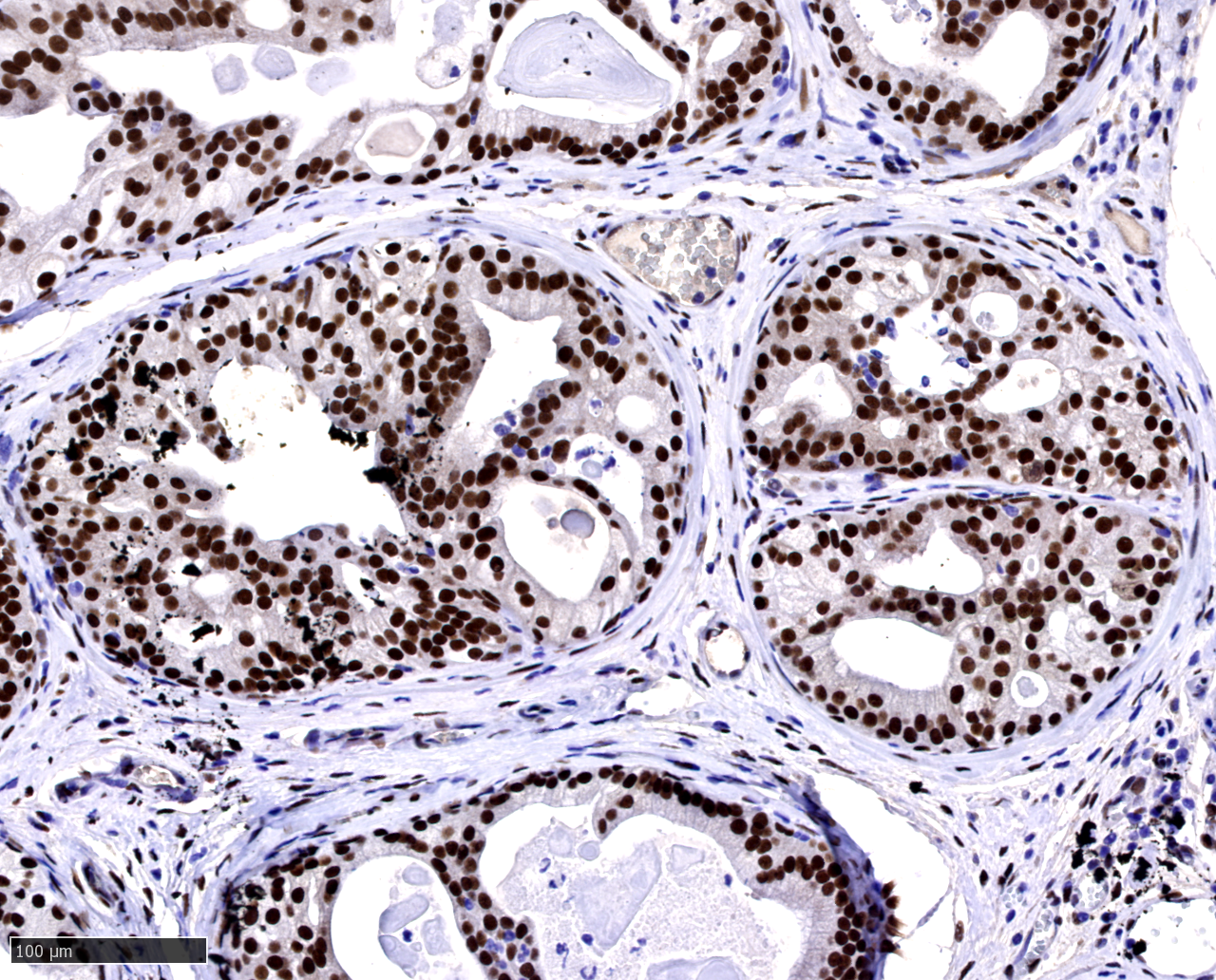

Supplement: Supplementary file 7 — Source Data for Figure 3 [file EMMM-15-e17209-s008.zip › Fig 3/3_E/PTENHIF_3S5_Ar.tif]

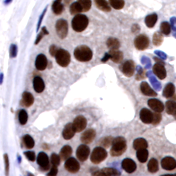

Supplement: Supplementary file 7 — Source Data for Figure 3 [file EMMM-15-e17209-s008.zip › Fig 3/3_E/PTENHIF_3S5_Ar_zoom.tif]

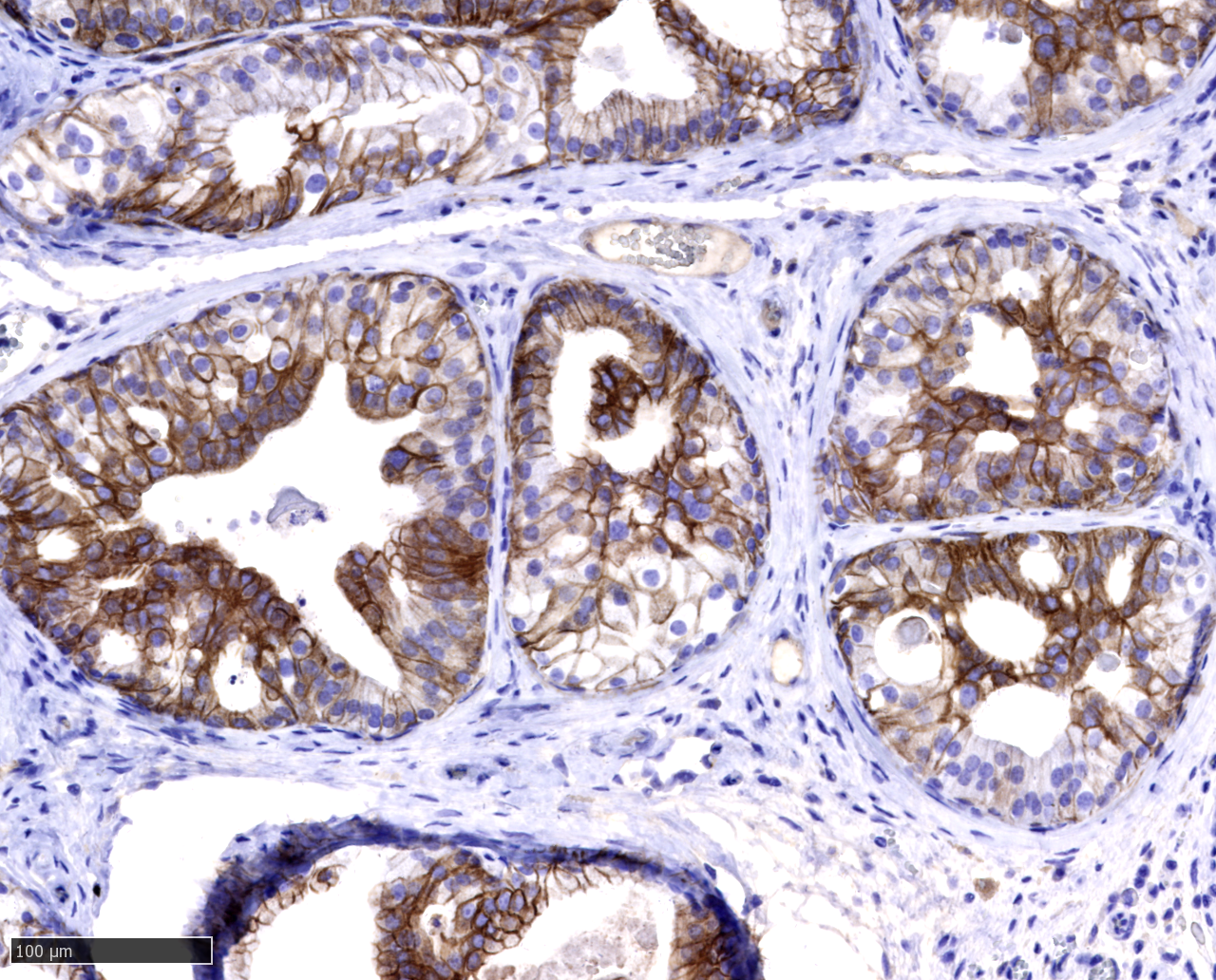

Supplement: Supplementary file 7 — Source Data for Figure 3 [file EMMM-15-e17209-s008.zip › Fig 3/3_E/PTENHIF_3S5_trop2.tif]

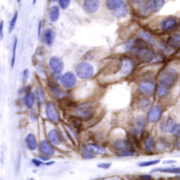

Supplement: Supplementary file 7 — Source Data for Figure 3 [file EMMM-15-e17209-s008.zip › Fig 3/3_E/PTENHIF_3S5_trop2_zoom.tif]

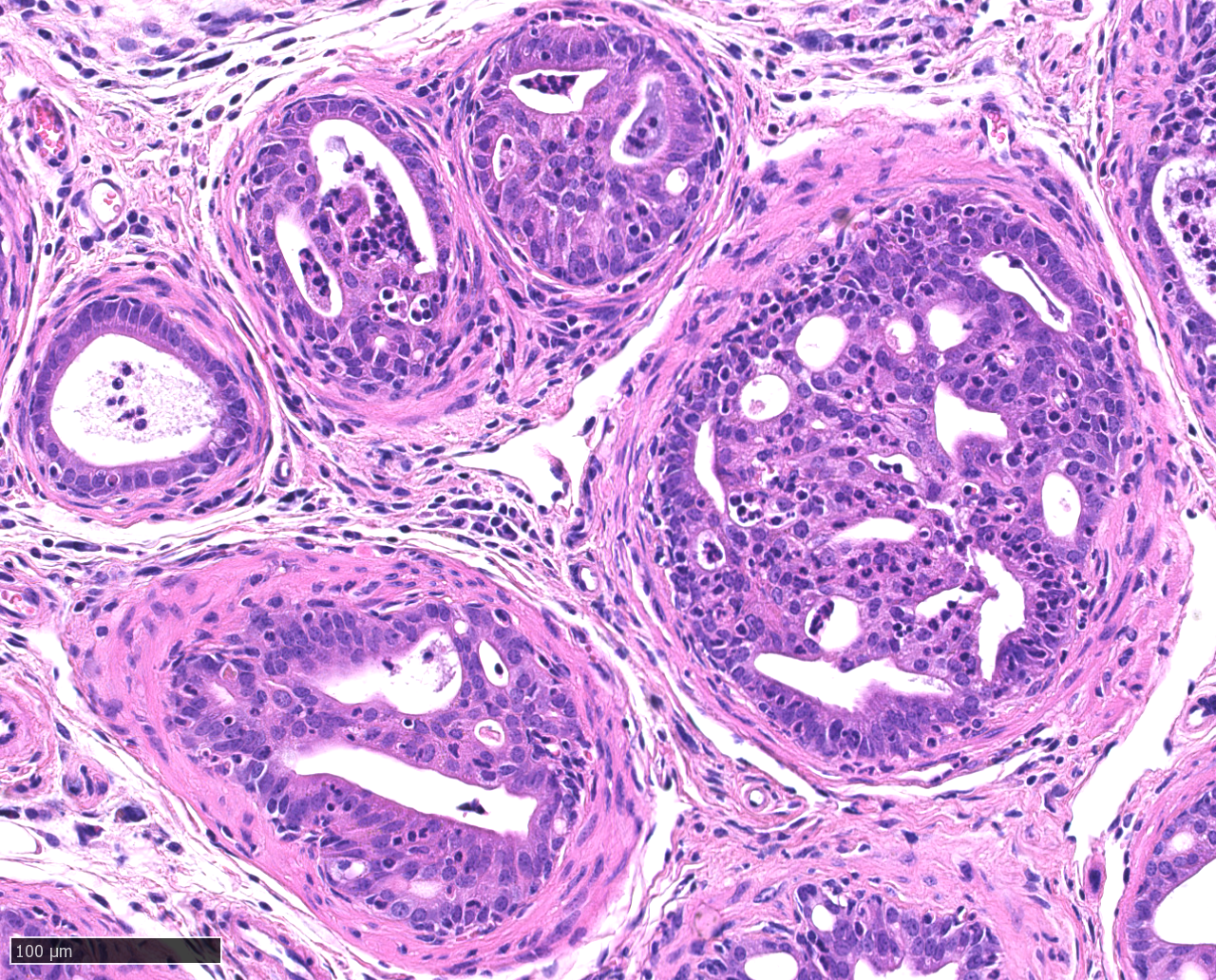

Supplement: Supplementary file 8 — Source Data for Figure 4 [file EMMM-15-e17209-s002.zip › Fig 4/4_B/PTEN_3C1_Tg_100_HE - 2021-11-18 16.58.48.tif]

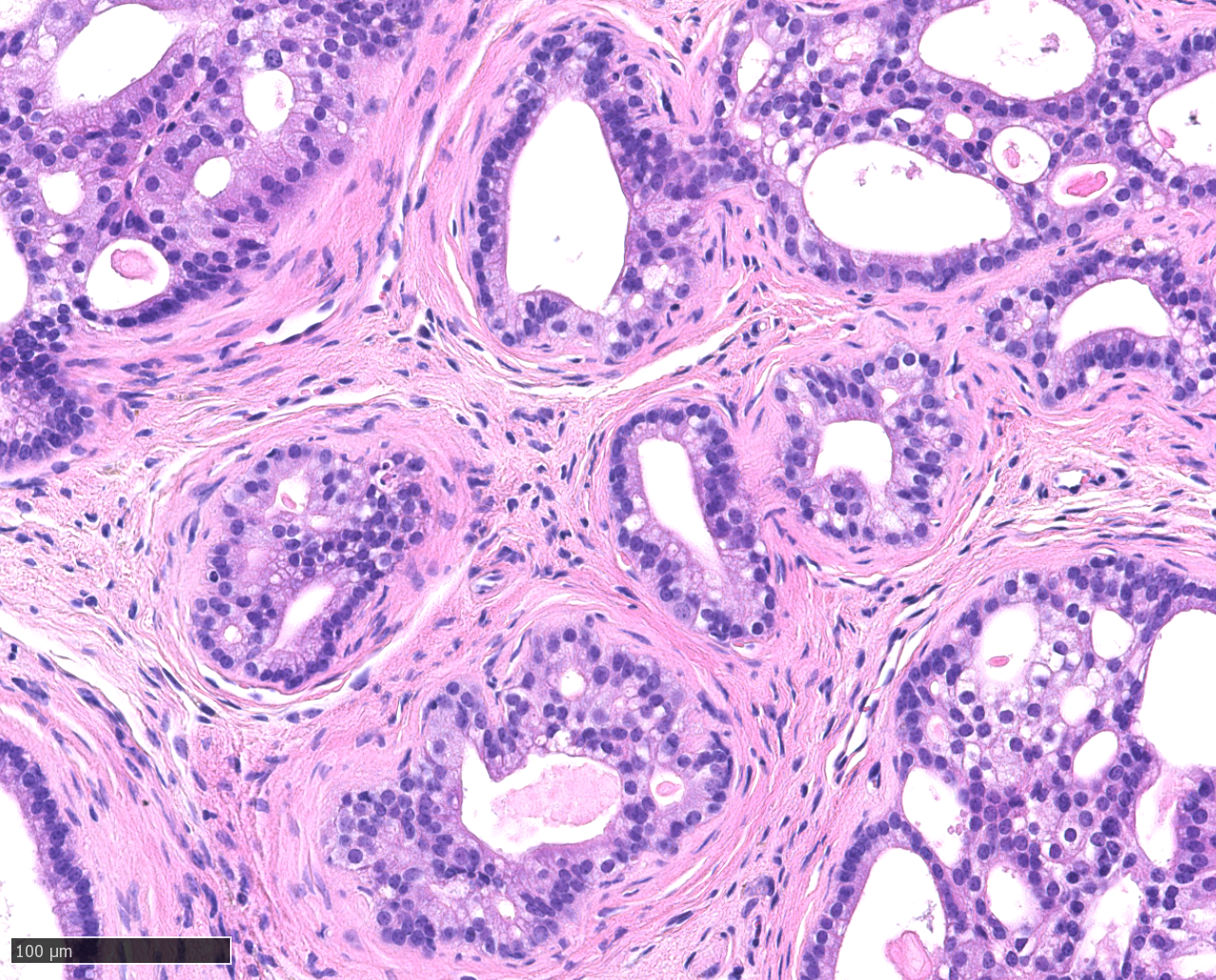

Supplement: Supplementary file 8 — Source Data for Figure 4 [file EMMM-15-e17209-s002.zip › Fig 4/4_B/PTEN_4C1_Px+pimo_tg166_HE - 2022-02-08 18.41.21_2.tif]

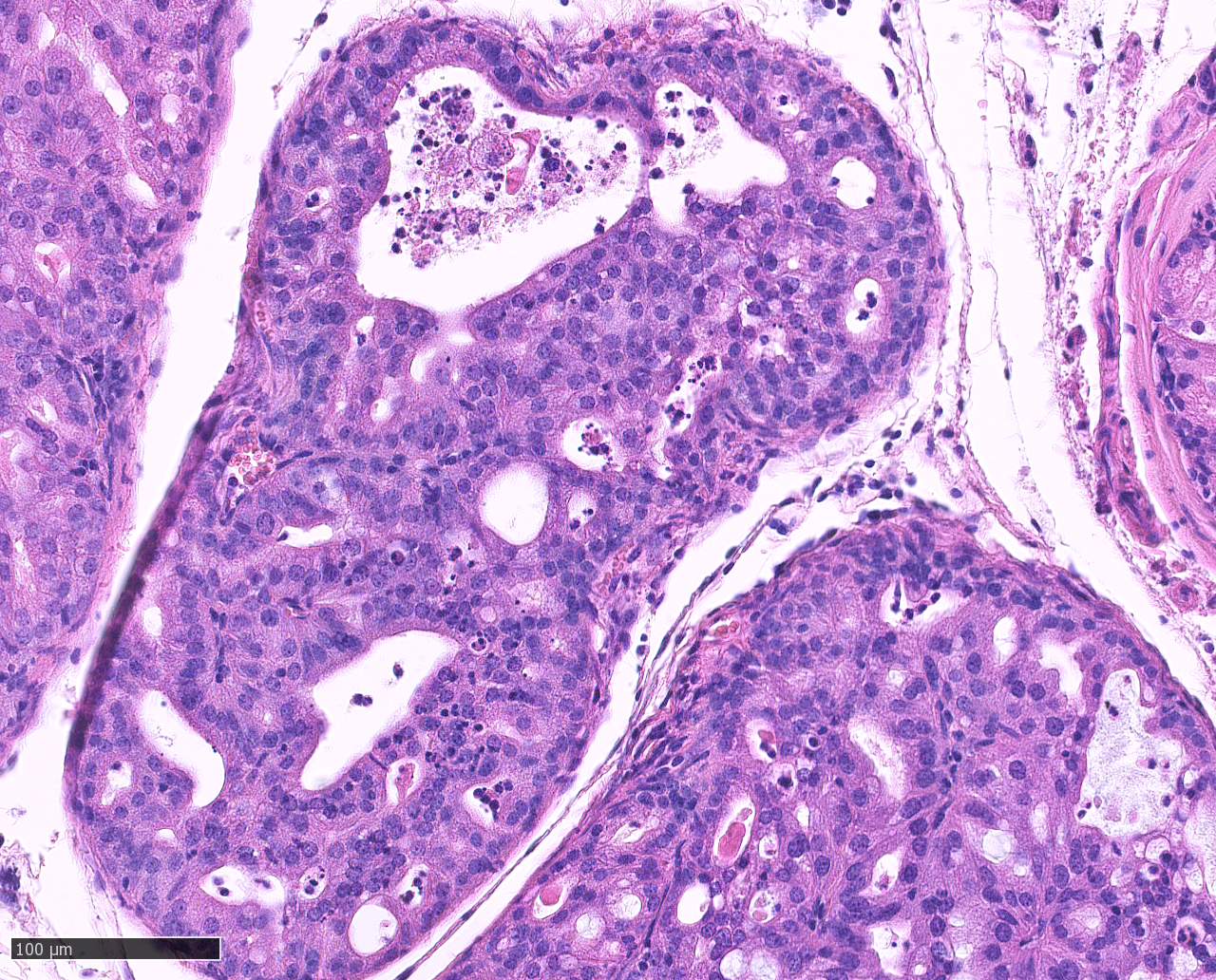

Supplement: Supplementary file 8 — Source Data for Figure 4 [file EMMM-15-e17209-s002.zip › Fig 4/4_B/PTEN_sham_vehicle_HE_20x.tif]

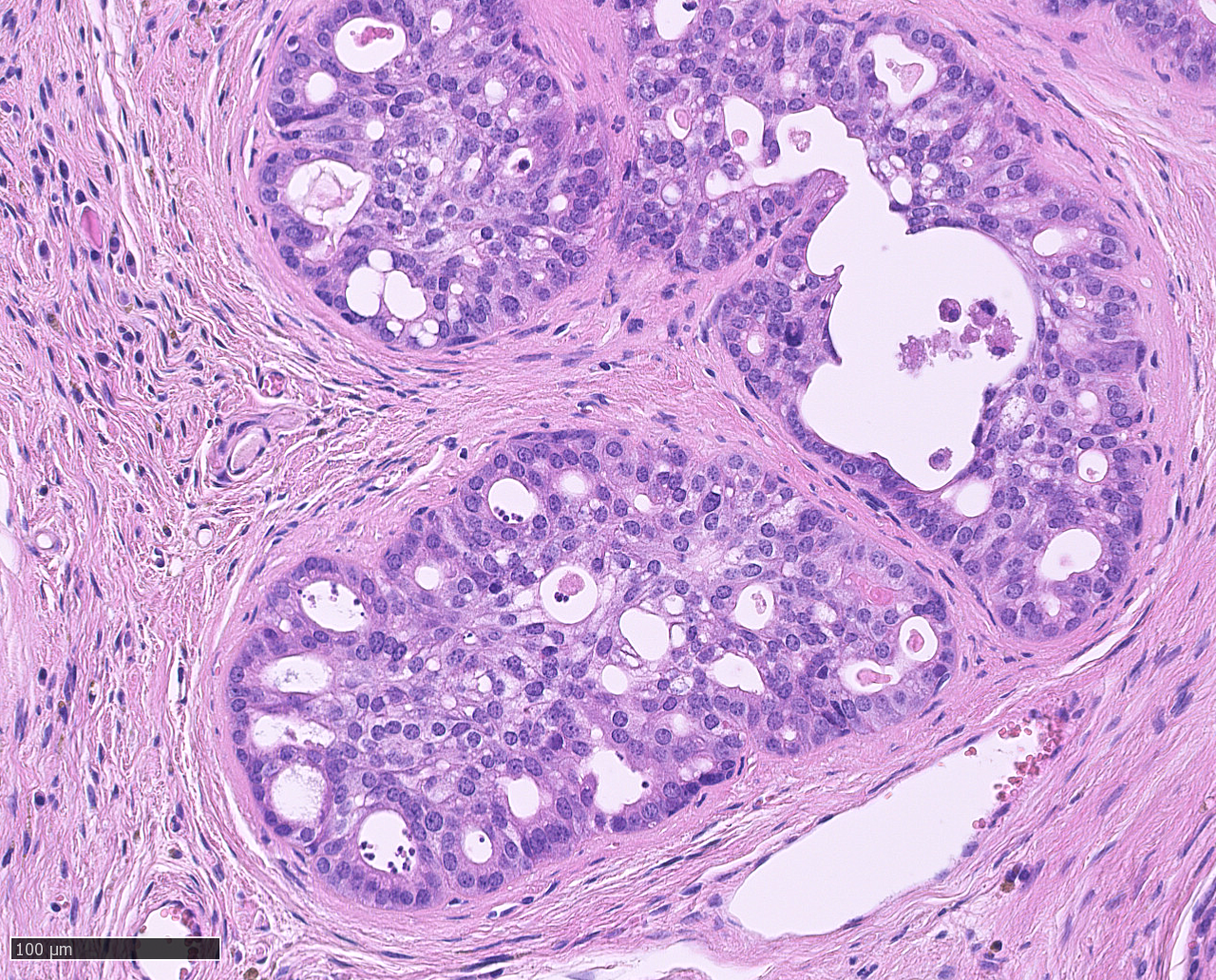

Supplement: Supplementary file 8 — Source Data for Figure 4 [file EMMM-15-e17209-s002.zip › Fig 4/4_B/Px_sham_Tg173_4mo_HE.tif]

C4-2B

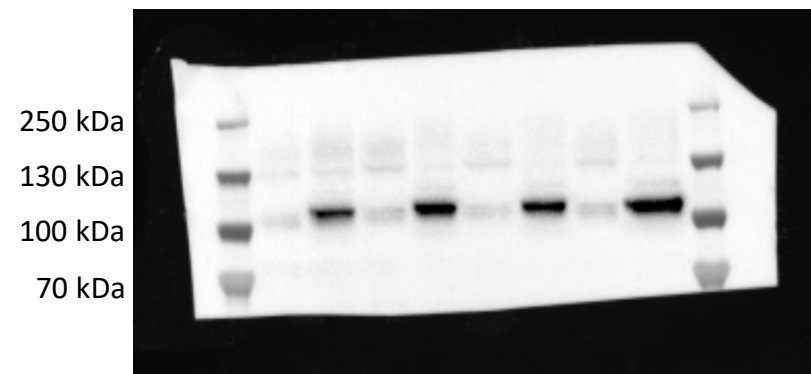

Cleaved PARP

C4-2B

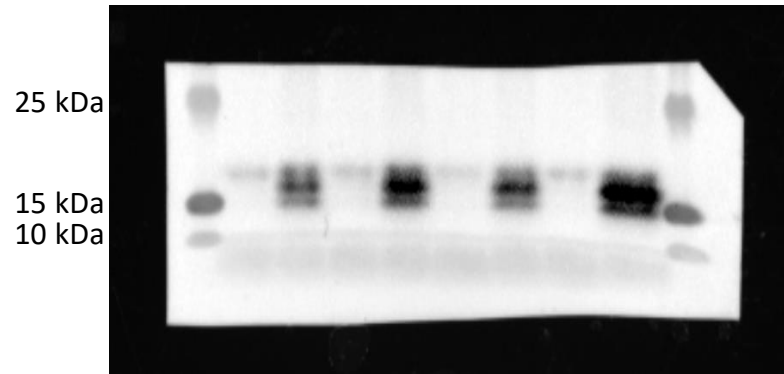

Cleaved caspase 3

C4-2B

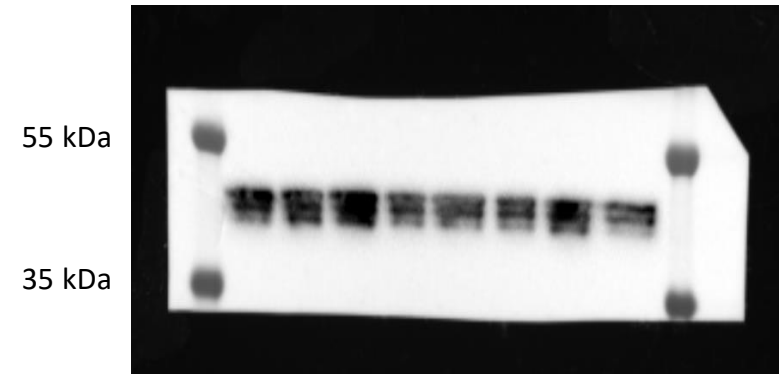

Beta-actin

Supplement: Supplementary file 8 — Source Data for Figure 4 [file EMMM-15-e17209-s002.zip › Fig 4/4_D/Fig4D_source data.pdf]

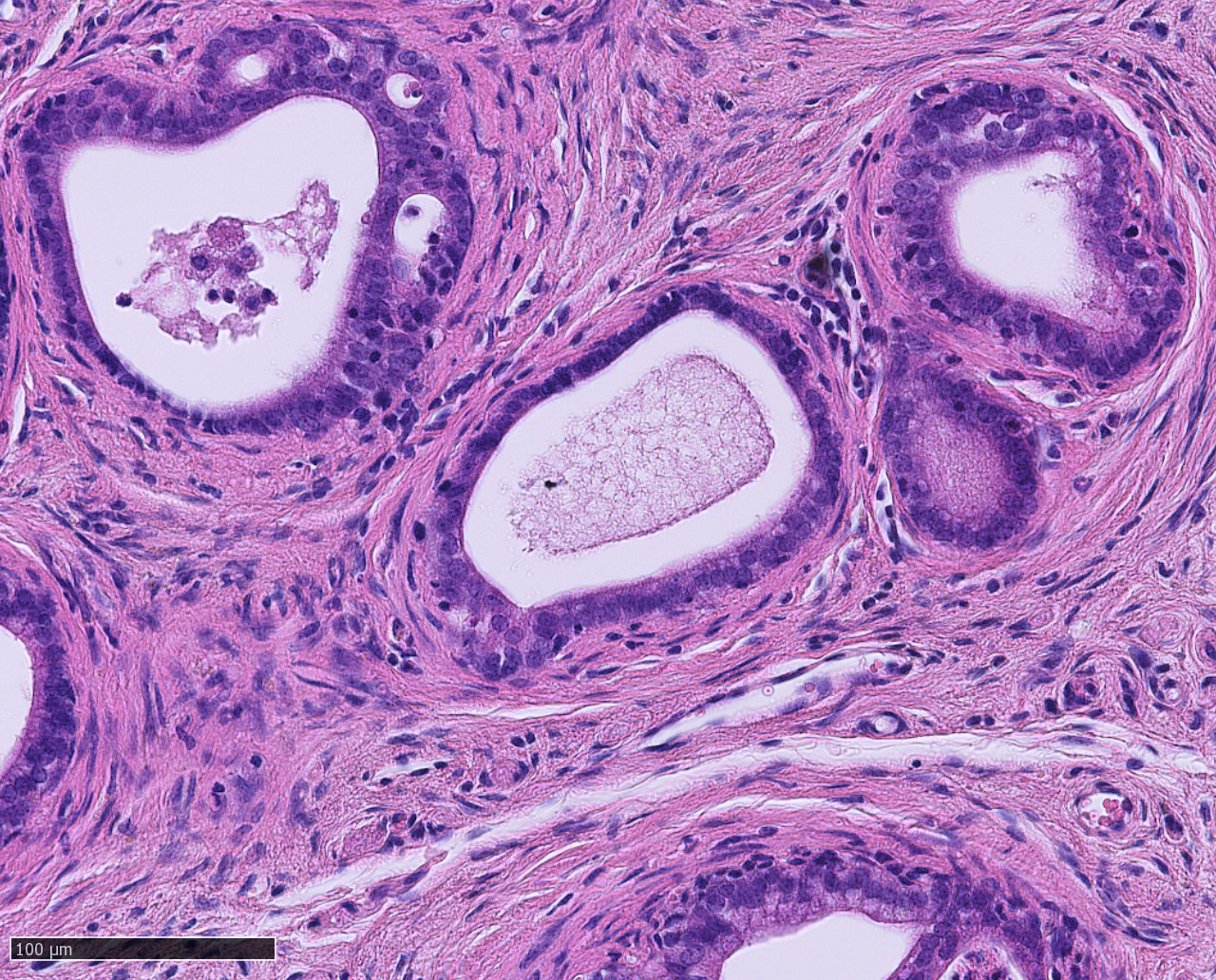

Supplement: Supplementary file 8 — Source Data for Figure 4 [file EMMM-15-e17209-s002.zip › Fig 4/4_F/PTEN_CTX_Px_revers_expr.tif]

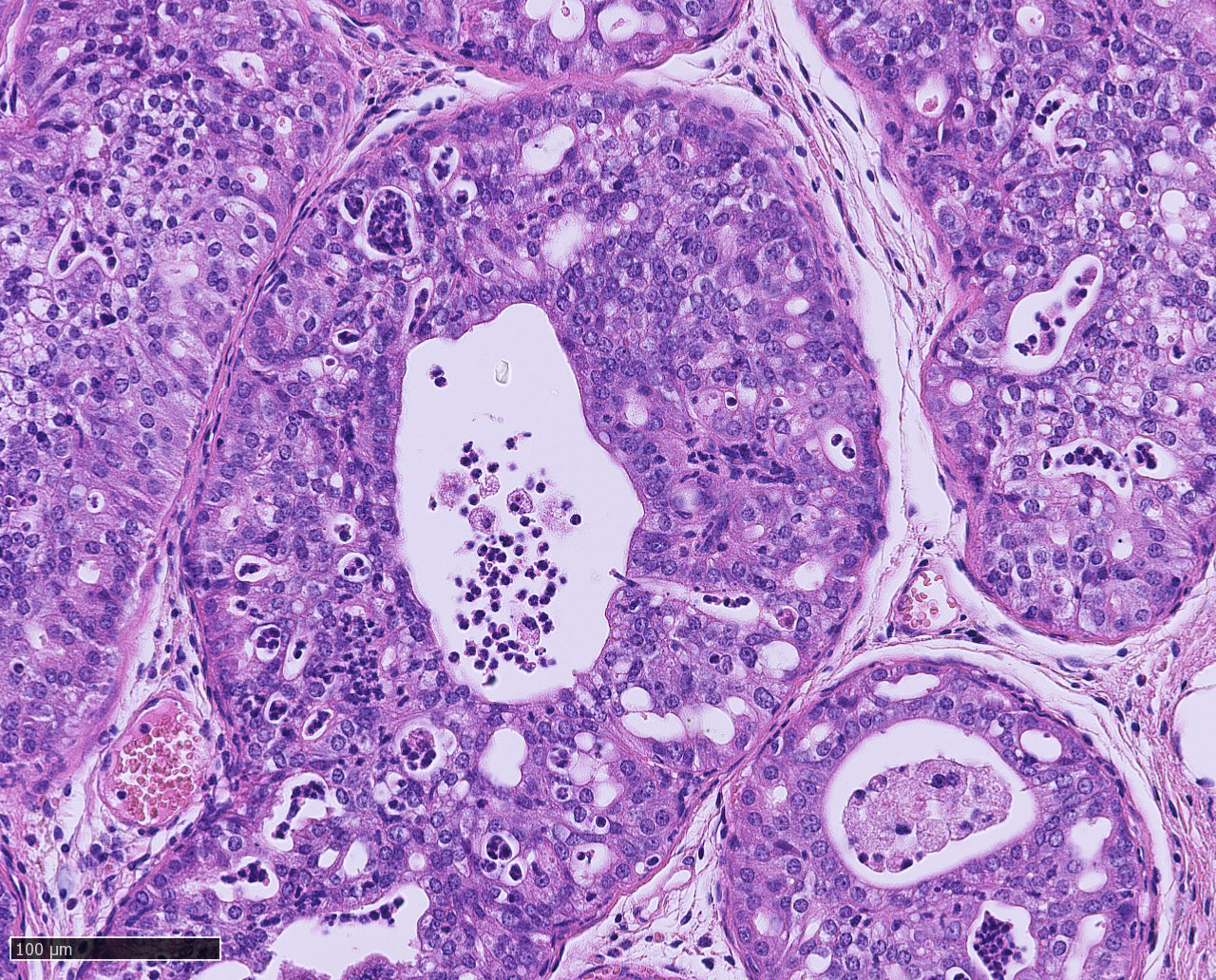

Supplement: Supplementary file 8 — Source Data for Figure 4 [file EMMM-15-e17209-s002.zip › Fig 4/4_F/PTEN_CTX_veh_revers_expr.tif]

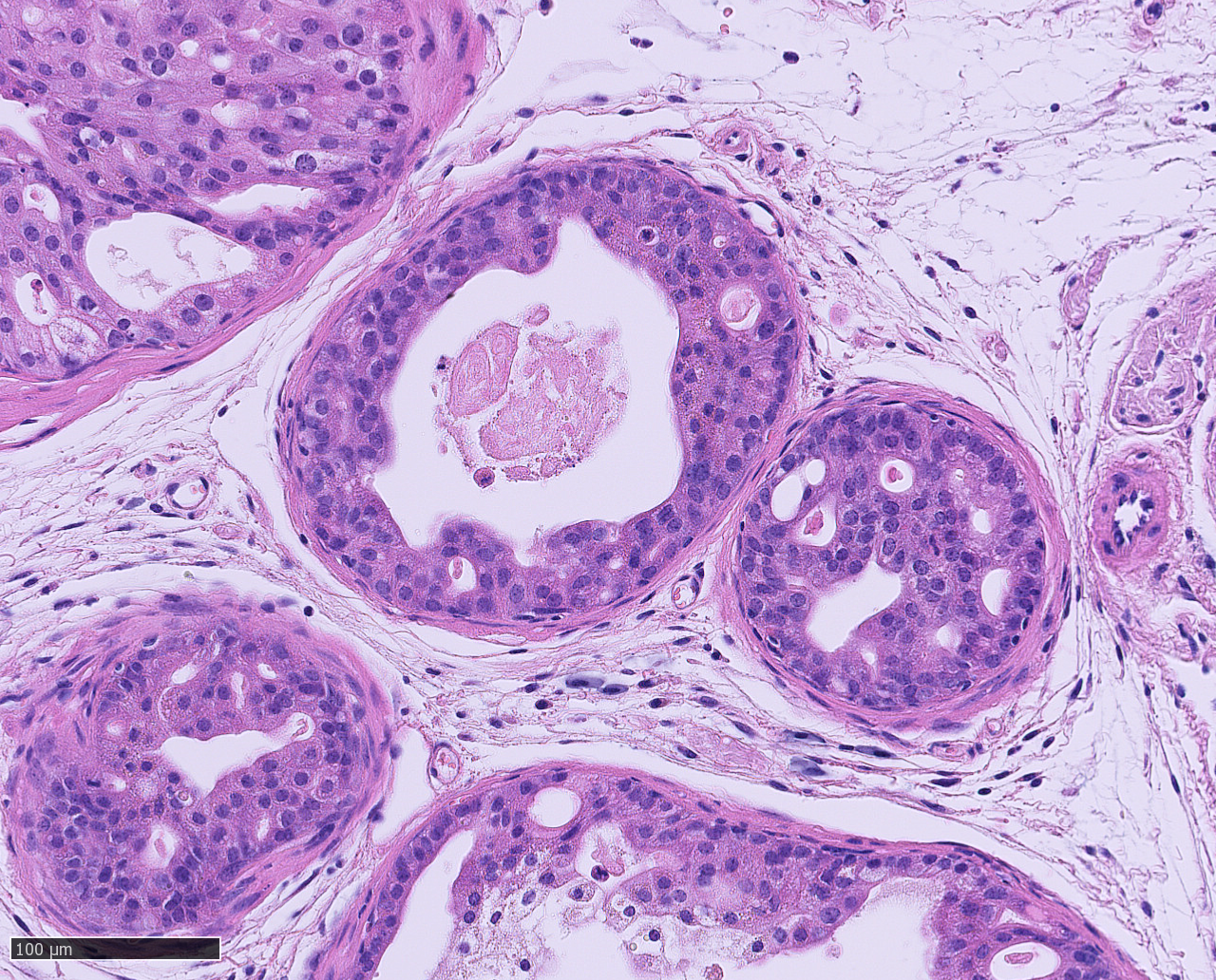

Supplement: Supplementary file 8 — Source Data for Figure 4 [file EMMM-15-e17209-s002.zip › Fig 4/4_F/PTEN_sham_Px_revers_expr.tif]
