# Supplementary material for: Hypoxia‐inducible factor 1A inhibition overcomes castration resistance of prostate tumors
Source: EMBO Mol Med. 2023 Apr 18;15(6):e17209. doi: 10.15252/emmm.202217209 (PMC10245031; doi:10.15252/emmm.202217209)
Supplement: Supplementary file 9 [file EMMM-15-e17209-s009.pdf]

EMBO Molecular Medicine

Hypoxia-inducible factor 1A inhibition overcomes castration resistance of prostate tumors

Julie Terzic, Mohamed Abu el Maaty, Régis Lutzing, Alexandre Vincent, Rana El Bizri, Matthieu Jung, Céline Keime, and Daniel Metzger
DOI: 10.15252/emmm.202217209

Corresponding author: Daniel Metzger (metzger@igbmc.fr)

Review Timeline:

Submission Date:	21st Nov 22
Editorial Decision:	12th Dec 22
Revision Received:	16th Feb 23
Editorial Decision:	9th Mar 23
Revision Received:	17th Mar 23
Accepted:	20th Mar 23

Editor: Lise Roth

Transaction Report:

(Note: With the exception of the correction of typographical or spelling errors that could be a source of ambiguity, letters and reports are not edited. Depending on transfer agreements, referee reports obtained elsewhere may or may not be included in this compilation. Referee reports are anonymous unless the Referee chooses to sign their reports.)

12th Dec 2022

Dear Dr. Metzger,

Thank you for the submission of your manuscript to EMBO Molecular Medicine. We have now received feedback from the three reviewers who agreed to evaluate your manuscript. As you will see from the reports below, the referees acknowledge the interest of the study and are overall supporting publication of your work pending appropriate revisions.

Addressing the reviewers' concerns in full will be necessary for further considering the manuscript in our journal, and acceptance of the manuscript will entail a second round of review. In particular, it will be important to increase the human relevance of your findings (human samples or cell lines). EMBO Molecular Medicine encourages a single round of revision only and therefore, acceptance or rejection of the manuscript will depend on the completeness of your responses included in the next, final version of the manuscript. For this reason, and to save you from any frustrations in the end, I would strongly advise against returning an incomplete revision.

If you would like to discuss further the points raised by the referees, I am available to do so via email or video. Let me know if you are interested in this option.

Revised manuscripts should be submitted within three months of a request for revision; they will otherwise be treated as new submissions, except under exceptional circumstances in which a short extension is obtained from the editor.

When submitting your revised manuscript, please carefully review the instructions that follow below. We perform an initial quality control of all revised manuscripts before re-review; failure to include requested items will delay the evaluation of your revision.

We require:

1) A .docx formatted version of the manuscript text (including legends for main figures, EV figures and tables). Please make sure that the changes are highlighted to be clearly visible.

2) Individual production quality figure files as .eps, .tif, .jpg (one file per figure). For guidance, download the 'Figure Guide PDF' (https://www.embopress.org/page/journal/17574684/authorguide#figureformat).

3) At EMBO Press we ask authors to provide source data for the main figures. Our source data coordinator will contact you to discuss which figure panels we would need source data for and will also provide you with helpful tips on how to upload and organize the files.

4) A .docx formatted letter INCLUDING the reviewers' reports and your detailed point-by-point responses to their comments. As part of the EMBO Press transparent editorial process, the point-by-point response is part of the Review Process File (RPF), which will be published alongside your paper.

5) A complete author checklist, which you can download from our author guidelines (https://www.embopress.org/page/journal/17574684/authorguide#submissionofrevisions). Please insert information in the checklist that is also reflected in the manuscript. The completed author checklist will also be part of the RPF.

6) It is mandatory to include a 'Data Availability' section after the Materials and Methods. Before submitting your revision, primary datasets produced in this study need to be deposited in an appropriate public database, and the accession numbers and database listed under 'Data Availability'. Please remember to provide a reviewer password if the datasets are not yet public (see https://www.embopress.org/page/journal/17574684/authorguide#dataavailability).

In case you have no data that requires deposition in a public database, please state so in this section. Note that the Data Availability Section is restricted to new primary data that are part of this study.

7) For data quantification: please specify the name of the statistical test used to generate error bars and P values, the number (n) of independent experiments (specify technical or biological replicates) underlying each data point and the test used to calculate p-values in each figure legend. The figure legends should contain a basic description of n, P and the test applied. Graphs must include a description of the bars and the error bars (s.d., s.e.m.). Please provide exact p values.

8) Our journal encourages inclusion of *data citations in the reference list* to directly cite datasets that were re-used and obtained from public databases. Data citations in the article text are distinct from normal bibliographical citations and should directly link to the database records from which the data can be accessed. In the main text, data citations are formatted as follows: "Data ref: Smith et al, 2001" or "Data ref: NCBI Sequence Read Archive PRJNA342805, 2017". In the Reference list, data citations must be labeled with "[DATASET]". A data reference must provide the database name, accession

number/identifiers and a resolvable link to the landing page from which the data can be accessed at the end of the reference. Further instructions are available at .

9) We replaced Supplementary Information with Expanded View (EV) Figures and Tables that are collapsible/expandable online. A maximum of 5 EV Figures can be typeset. EV Figures should be cited as 'Figure EV1, Figure EV2" etc... in the text and their respective legends should be included in the main text after the legends of regular figures.
- For the figures that you do NOT wish to display as Expanded View figures, they should be bundled together with their legends in a single PDF file called *Appendix*, which should start with a short Table of Content. Appendix figures should be referred to in the main text as: "Appendix Figure S1, Appendix Figure S2" etc.
- Additional Tables/Datasets should be labeled and referred to as Table EV1, Dataset EV1, etc. Legends have to be provided in a separate tab in case of .xls files. Alternatively, the legend can be supplied as a separate text file (README) and zipped together with the Table/Dataset file.
See detailed instructions here:

10) The paper explained: EMBO Molecular Medicine articles are accompanied by a summary of the articles to emphasize the major findings in the paper and their medical implications for the non-specialist reader. Please provide a draft summary of your article highlighting
- the medical issue you are addressing,
- the results obtained and
- their clinical impact.
This may be edited to ensure that readers understand the significance and context of the research. Please refer to any of our published articles for an example.

11) For more information: There is space at the end of each article to list relevant web links for further consultation by our readers. Could you identify some relevant ones and provide such information as well? Some examples are patient associations, relevant databases, OMIM/proteins/genes links, author's websites, etc...

12) Author contributions: CRediT has replaced the traditional author contributions section because it offers a systematic machine readable author contributions format that allows for more effective research assessment. Please remove the Authors Contributions from the manuscript and use the free text boxes beneath each contributing author's name in our system to add specific details on the author's contribution. More information is available in our guide to authors.

13) Disclosure statement and competing interests: We updated our journal's competing interests policy in January 2022 and request authors to consider both actual and perceived competing interests. Please review the policy https://www.embopress.org/competing-interests and update your competing interests if necessary.

14) Every published paper now includes a 'Synopsis' to further enhance discoverability. Synopses are displayed on the journal webpage and are freely accessible to all readers. They include a short stand first (maximum of 300 characters, including space) as well as 2-5 one-sentences bullet points that summarizes the paper. Please write the bullet points to summarize the key NEW findings. They should be designed to be complementary to the abstract - i.e. not repeat the same text. We encourage inclusion of key acronyms and quantitative information (maximum of 30 words / bullet point). Please use the passive voice. Please attach these in a separate file or send them by email, we will incorporate them accordingly.
Please also suggest a striking image or visual abstract to illustrate your article as a PNG file 550 px wide x 300-600 px high.

15) As part of the EMBO Publications transparent editorial process initiative (see our Editorial at http://embomolmed.embopress.org/content/2/9/329), EMBO Molecular Medicine will publish online a Review Process File (RPF) to accompany accepted manuscripts.
In the event of acceptance, this file will be published in conjunction with your paper and will include the anonymous referee reports, your point-by-point response and all pertinent correspondence relating to the manuscript. Let us know whether you agree with the publication of the RPF and as here, if you want to remove or not any figures from it prior to publication.
Please note that the Authors checklist will be published at the end of the RPF.

EMBO Molecular Medicine has a "scooping protection" policy, whereby similar findings that are published by others during review or revision are not a criterion for rejection. Should you decide to submit a revised version, I do ask that you get in touch after three months if you have not completed it, to update us on the status.

I look forward to receiving your revised manuscript.

With kind regards,

Lise

Lise Roth, PhD

Senior Editor
EMBO Molecular Medicine

***** Reviewer's comments *****

Referee #1 (Comments on Novelty/Model System for Author):

Everything that will improve the article has been detailed below.
This is a very innovative article because the role of HIF1 had not been considered in the castration resistance process in prostate cancer. These results are very promising for the development of new therapeutic approaches.

Referee #1 (Remarks for Author):

Title: Hypoxia-inducible factor 1A inhibition overcomes castration resistance of prostate tumors.

Authors: Julie Terzic, Mohamed A. Abu el Maaty, Régis Lutzing, Alexandre Vincent, Rana El Bizri, Matthieu Jung, Céline Keime, and Daniel Metzger.

Comments

Overall, this is an interesting paper to read, with sufficient evidence from both mouse models and organoid samples to support their conclusions. The experiments were well designed and executed. The conclusion will provide useful guidance to propose a more effective therapeutic treatment. With some further clarification and additional experimentation, this manuscript is worthy of consideration for publication in EMBO Molecular Medicine.

Major comments

1- Throughout the article, the authors seem to confuse HIF-1A with HIF-1. The former is the hypoxia-regulated subunit, the latter is the transcriptional factor. It is clear that it is the HIF-1A subunit that is invalidated, but it is not solely responsible for the variation in cell response to hypoxia. The authors cannot write "PTEN after puberty, are hypoxic and that enhanced hypoxia-inducible factor 1A (HIF1A) signaling drives the malignant evolution...". Only the transcription factor HIF1 can influence the cell response to hypoxia.
2- The results are rather difficult to read because the authors punctuate them with too many references that should not be at the level of the results but rather either in the Introduction or in the Discussion.
3- Fig. S1C - In order to allow a smoother reading and to understand that the 16106 cells of which the authors speak are the sum of 6166 + 9940 cells, it could be simpler to say "Analysis of these 16106 cells identified ...". The question will be less raised. The description of the cells and their belonging to this or that cluster is not easy to read. Would it be possible to start the numbering with 1 and not with 0?The quality of Figure S1 is not very good. The choice of colors does not allow an easy identification between the different clusters. It is also not easy to read the cluster numbers.
4- Fig. 1D - The choice of colors does not allow an easy identification between the different clusters. It is also not easy to read the cluster numbers.
5- Why are the 11 hypoxia genes not present in the list of 380 genes in Table S1?
6- Why focus on TGM2 (Figure 1L) when it is not listed?
7- Why focus on TROP 2 (Figure 2E)? On all the IHC images in the whole article, the authors should provide a magnification because one never sees the localization in the cells.
8- Fig. 2G - after 30 days of castration, does the morphology of the prostate in Pten/HIF look like the morphology of a normal prostate?
9- Fig. 2I - do Pten and Ptet/HIF organoids have the same growth rate? As HIF1 can activate autophagy, could it be possible to envisage a balance between less autophagy and thus more apoptosis which would be responsible for this return to normal?
10- Fig. 3B - Does the morphology of the prostate in Pten/HIF look like the morphology of a normal prostate?
11- Figure 3E - Enlarged.
12- The reference (Lee & Kim, 2011) should at least be replaced by Koh et al. 2008 Mol. Cancer Ther.
13- Could the authors comment on HIF-2a and HIF2? Did they observe a compensatory effect in Pten/HIF1A mice or with the compound PX-478? If HIF-2 is not inhibited, is it then possible to envisage that HIF-2 could activate stem cells over time and promote tumor recovery? Have the authors tried on Pten/HIF1A organoids to use PT2385 (HIF-2 inhibitor)?

Minor comments
1- In the Introduction, the authors wrote "Although initially effective, many patients relapse ". Many being unspecific, could the

authors be a little more specific?
2- Same remark for "improves survival of CRPC patients by several months".
3- Figure 1C should appear right after (Cpa3 and MCPT4).
4- Via in italic.
5- The list of 380 genes should be better organized and presented. The names of some genes are cut off.
6- Figure legends and not Figures Legends.
7- Figure 1: instead of Figure 1.:
8- Figure 1: AR, DLP and AGI should be defined.
9- Figure 4: 0.05 instead of 0,05

Referee #2 (Comments on Novelty/Model System for Author):

The authors assert that the inhibition of Hif1 by genetic or pharmacological manipulation in mice tumors deficient for Pten resulted in a recover from the castration resistance disease.
This is a significant finding and point out Hif1 as a new molecular target against CRPC.
The experiments are well performed and the in vivo model provides a strong support for the applicability of their results to the human disease.
It is a relevant discovery and it might be applied to other tumor types where PTEN is deleted.

The paper would be greatly strengthened if similar evidences can be observed in human tumors.

Referee #2 (Remarks for Author):

The authors assert that the inhibition of Hif1 by genetic or pharmacological manipulation in mice tumors deficient for Pten resulted in a recover from the castration resistance disease.

This is a significant finding and point out Hif1 as a new molecular target against CRPC. This relevant discovery might be applied to other tumor types where PTEN is deleted.

Referee #3 (Comments on Novelty/Model System for Author):

valuable GEMM of CRPC

Referee #3 (Remarks for Author):

This study focuses on mechanisms by which prostatic intraepithelial neoplasia (PIN) lesions (considered precursors to prostate adenocarcinoma) in Pten-KO mice persist following androgen deprivation (castration). The authors identified a subpopulation of cells termed Luminal-C cells in Pten-KO mice and that this subpopulation is resistant to androgen deprivation. Droplet-based single-cell RNA-seq revealed that hypoxia-related genes were upregulated after castration. Using double KO mice (Pten and HIF1a) the authors showed that HIF1a prevents cell death after castration. This long-term effect was observed up to five months after castration. PX-478 an orally available inhibitor of HIF1A effectively prevented castration resistance in Pten-KO mice. PX-478 administration after castration-resistant prostate cancer lesions were already established also reduced the number of PINs and adenocarcinoma. This manuscript demonstrates the importance of HIF1a in prostate cancer progression and nominates HIF1a as promising target for incurable CRPC.
Specific comments:
• The authors should better highlight that Luminal C cells are increased in Pten(i)pe-/- vs WT mice, thereby allowing the reader to understand the importance of this sub-population in disease progression.
• Fig. 1D. can changes in subpopulations be quantified? A graph showing this would be helpful.
• Although Supple Fig 1E shows the two specific subpopulations (luminal-A and -B), the color coding in Fig 1D is confusing, since many shades are very similar, it would help if luminal A and C cells would be indicated by an arrow or another identifier.
• Fig 1E and G. Are these the top hits? Were apoptosis related gene sets upregulated in CTX vs sham?
• Could the authors explain the x axis of graphs E and G (-log2 FDR)?
• The authors report PIN and adenocarcinoma in dorsolateral prostate (DLP) of castrated Pten(i)pe-/- mice. Were other prostate lobes affected as well?
• Figure 2A and C - How does this compare to total weight of DLP and gland area in sham and castrated Pten(i)pe-/ mice?
• Fig 2 and 3 - when referring to prostate weight, is this only DLP or the whole prostate?
• Could authors explain the staining of cleaved caspase 3 in the lumen of the acini (Figure 2G)?
• Detection of other apoptotic markers and proliferation markers would strengthen the CC3 IHC (and WB in C4-2B) results.
• Why were such high concentrations of enzalutamide used for the organoid experiments? Experiments with an additional AR antagonist could rule out potential off-target effects.

- Panel 3 shows that Pten/HIF1a KO response to castration is maintained for long periods of time, could this be reverted by testosterone administration?
- It is unclear why enzalutamide-sensitive C4-2B cells don't respond to enzalutamide treatment with increased CC3.
- How do authors speculate that HIF1A is activated in C4-2B in normoxic conditions?
- In the discussion, the authors argue that androgen deprivation promotes an immunosuppressive environment. Can this be further clarified? RNA-seq data on luminal C cells shows that castration induces upregulation of inflammation-related pathways and expression of certain pro-inflammatory cytokines is higher in CTX vs Sham.
- Is HIF1a overexpressed in Pten(i)pe-/- sham or castrated mice vs WT?
- Was mouse weight measured after PX-478 administration?
- Impact would be enhanced if the authors can describe relevant correlates in human samples or cell lines

Referee #1 (Comments on Novelty/Model System for Author):

Everything that will improve the article has been detailed below.

This is a very innovative article because the role of HIF1 had not been considered in the castration resistance process in prostate cancer. These results are very promising for the development of new therapeutic approaches.

Referee #1 (Remarks for Author):

Title: Hypoxia-inducible factor 1A inhibition overcomes castration resistance of prostate tumors.

Authors: Julie Terzic, Mohamed A. Abu el Maaty, Régis Lutzing, Alexandre Vincent, Rana El Bizri, Matthieu Jung, Céline Keime, and Daniel Metzger.

Comments

Overall, this is an interesting paper to read, with sufficient evidence from both mouse models and organoid samples to support their conclusions. The experiments were well designed and executed. The conclusion will provide useful guidance to propose a more effective therapeutic treatment. With some further clarification and additional experimentation, this manuscript is worthy of consideration for publication in EMBO Molecular Medicine.

Response: We thank the reviewer for his/her very positive comments and constructive suggestions.

Major comments

1- Throughout the article, the authors seem to confuse HIF-1A with HIF-1. The former is the hypoxia-regulated subunit, the latter is the transcriptional factor. It is clear that it is the HIF-1A subunit that is invalidated, but it is not solely responsible for the variation in cell response to hypoxia. The authors cannot write "PTEN after puberty, are hypoxic and that enhanced hypoxia-inducible factor 1A (HIF1A) signaling drives the malignant evolution...". Only the transcription factor HIF1 can influence the cell response to hypoxia.

Response: This has been updated, and now reads:

- line 39: *"In addition, hypoxia-inducible factor 1 (HIF1) signaling, which we have previously demonstrated to be induced in luminal cells of Pten$^{(i)pe-/-}$ mice and to promote malignant progression, is further activated."*

- line 82 : *"We have previously shown that prostatic intraepithelial neoplasia (PIN) of Pten$^{(i)pe-/-}$ mice, generated by luminal epithelial cell-specific deletion of the tumor suppressor PTEN after puberty, are hypoxic and that enhanced hypoxia-inducible factor 1 (HIF1) signaling in luminal cells drives the malignant evolution of such lesions (Abu El Maaty et al, 2022)."*

2- The results are rather difficult to read because the authors punctuate them with too many references that should not be at the level of the results but rather either in the Introduction or in the Discussion.

Response: Simplifications in the text of the results section have been made where possible, including the removal of a number of references.

3- Fig. S1C - In order to allow a smoother reading and to understand that the 16106 cells of which the authors speak are the sum of 6166 + 9940 cells, it could be simpler to say "Analysis of these 16106 cells identified ...". The question will be less raised. The description of the cells and their belonging to this or that cluster is not easy to read. Would it be possible to start the numbering with 1 and not with 0?

The quality of Figure S1 is not very good. The choice of colors does not allow an easy identification between the different clusters. It is also not easy to read the cluster numbers.

Response: As suggested, we modified the description in the manuscript (line 113). scRNA-seq data analysis using the Seurat package generates clusters with numbers starting with 0, a nomenclature that has been previously used in the literature (e.g. Chen et al. Nat Cell Biol. 2021). However, as requested, we modified the numbering of the clusters which now starts with cluster 1 (Fig EV1E) and the text accordingly (line 113-117). Moreover, we changed the colors of the different clusters and enlarged their numbers to facilitate their identification.

4- Fig. 1D - The choice of colors does not allow an easy identification between the different clusters. It is also not easy to read the cluster numbers.

Response: The colors have been modified. Moreover, Luminal-A and Luminal-C cells are clearly identified in the UMAPs of the new panel (Fig 1D), as suggested by reviewer 3.

5- Why are the 11 hypoxia genes not present in the list of 380 genes in Table S1?

Response: The 11 hypoxia genes were present in the list of 380 genes, in the folder “upregulated” of Table S1 in the Excel file (source file). Unfortunately, only the “downregulated” folder was converted as a pdf file. We have now modified Table EV1 to improve reading, and we added a column specifying the gene regulation (up- versus down-regulated) to avoid any confusion.
We listed them separately in a main table to allow the readers to identify easily the genes involved in the hypoxic signaling pathway.

6- Why focus on TGM2 (Figure 1L) when it is not listed?

Response: Although TGM2 is significantly upregulated in Luminal C cells of castrated mice compared to sham-operated ones (p-value= 0.00018; log2 fold change= 0.28097), the induction of its expression did not reach the stringent criteria we applied for differentially expressed genes (log2 fold change ≥ 0.4). As we show in the manuscript that the expression of stemness markers (*Cd44* and *Ly6d*) are induced in persistent luminal-C cells, and since we have shown that TGM2 is a marker of a high plasticity state in Pten luminal-C cells (Abu el Maaty et al. Sci Adv. 2022), we investigated its expression in prostatic sections of sham-operated and castrated Pten$^{(i)pe-/-}$ mice by immunohistochemistry, and observed a clear induction in its levels in the latter.
To justify the analysis of TGM2 expression, we added the following sentence (line 154-156): “*To further investigate this, we determined the levels of Transglutaminase 2 (TGM2), which we have recently shown to be a marker of HIF1A-driven disease progression and luminal plasticity (Abu El Maaty et al., 2022), in prostatic sections.*”

7- Why focus on TROP 2 (Figure 2E)?

Response: We previously validated TROP2 as a specific marker of luminal-C cells in Pten-deficient prostatic lesions (Abu el Maaty et al. Sci Adv. 2021; Abu el Maaty et al. Sci Adv. 2022). TROP2 stainings are provided to demonstrate the abundance of luminal-C cells in prostates of sham-operated and castrated mice.
This is now clarified in the manuscript, lines 173-176: ‘*Of note, immunohistochemical analyses showed that most of the remaining luminal cells of castrated Pten/Hif1a$^{(i)pe-/-}$ mice expressed the luminal-C marker TROP2 (Abu El Maaty et al., 2022) and expressed AR in the cytoplasm (Fig 2E)*’.

On all the IHC images in the whole article, the authors should provide a magnification because one never sees the localization in the cells.

Response: As requested, higher magnifications have been added in figures 1A and 1L, 2E, 2F, 2G and 3E.

8- Fig. 2G - after 30 days of castration, does the morphology of the prostate in Pten/HIF look like the morphology of a normal prostate?

Response: As described in the manuscript (line 204-205), more than 80% of glands in the dorsolateral prostates of Pten/Hif1a$^{(i)pe-/-}$ mice after 30 days of castration have a normal-like appearance. The remaining glands of castrated Pten/Hif1a$^{(i)pe-/-}$ mice contained PINs, which are not observed in normal prostates.

9- Fig. 2I - do Pten and Ptet/HIF organoids have the same growth rate?

Response: Our characterization of the organoids derived from prostates of both mouse lines revealed that those generated from Pten/Hif1a$^{(i)pe-/-}$ mice have a lower organoid formation capacity than those from Pten$^{(i)pe-/-}$ ones (see below), owing to the reduced stemness in the absence of HIF1A in prostatic luminal cells, as described in our previous work (Abu el Maaty et al. Sci Adv. 2022).

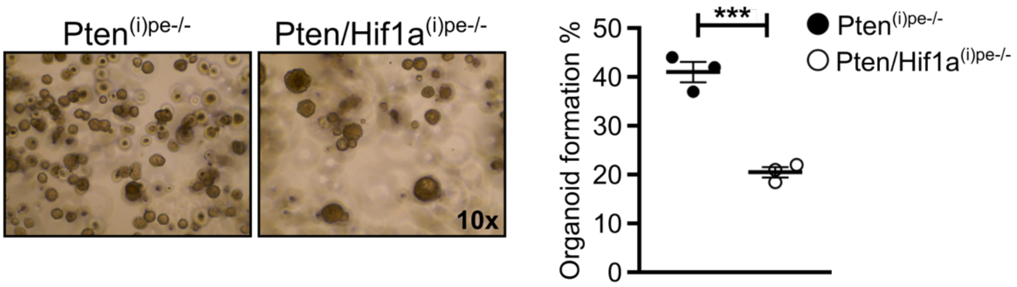

To circumvent any bias this might incur on the response of such organoids to enzalutamide treatments, we increased the initial seeding density of Pten/Hif1a$^{(i)pe-/-}$ organoids.

As HIF1 can activate autophagy, could it be possible to envisage a balance between less autophagy and thus more apoptosis which would be responsible for this return to normal?

The potential role of autophagy in the response of Pten-deficient prostatic tumors to androgen deprivation and the possible involvement of HIF1A in mediating this is an interesting prospect. Indeed, Nguyen et al. (doi.org/10.1038/onc.2014.25) have previously shown that enzalutamide treatment activates autophagy in CRPC cells. However, the role of autophagy in mediating the HIF1A-driven castration-resistance is out of the scope of this study.

10- Fig. 3B - Does the morphology of the prostate in Pten/HIF look like the morphology of a normal prostate?

Response: Please see response to point 8.

11- Figure 3E - Enlarged.

Response: As suggested, this figure has been enlarged.

12- The reference (Lee & Kim, 2011) should at least be replaced by Koh et al. 2008 Mol. Cancer Ther.

Response: The reference has been replaced, as suggested.

13- Could the authors comment on HIF-2a and HIF2? Did they observe a compensatory effect in Pten/HIF1A mice or with the compound PX-478? If HIF-2 is not inhibited, is it then possible to envisage that HIF-2 could activate stem cells over time and promote tumor recovery? Have the authors tried on Pten/HIF1A organoids to use PT2385 (HIF-2 inhibitor)?

Response: We performed immunostaining studies of HIF2A on prostatic sections of control and Pten$^{(i)pe-/-}$ mice at 1, 2 and 3 months AGI, and found that its levels are induced in Pten-deficient PINs.

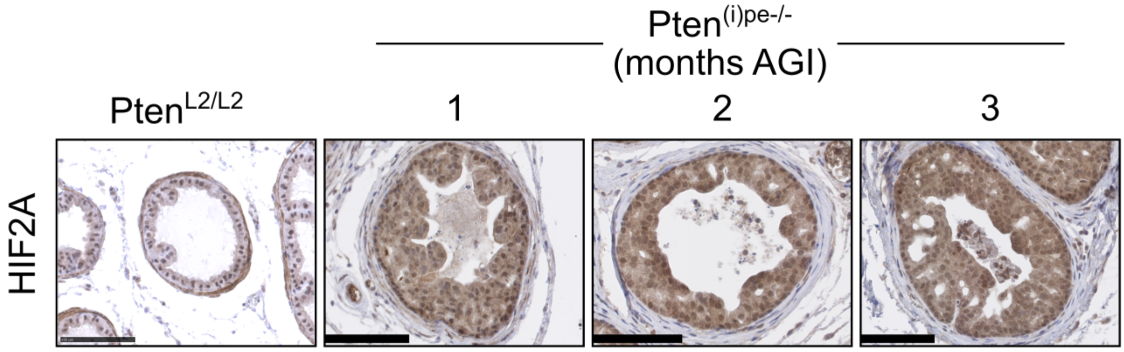

However, we previously showed that HIF1A loss markedly reduces disease progression over > 1 year (Abu el Maaty et al. Sci Adv. 2022), and demonstrate in this study that castration induces durable anti-tumor responses in Pten/Hif1a$^{(i)pe-/-}$ mice (Fig. 2 and 3), and that PX-478, which inhibits HIF1A (Koh et al. 2008) but not HIF2A (Welsh et al. doi.org/10.1158/1535-7163.233.3.3), also prevents castration resistance (Figure 4). Thus, having no evidence of a compensatory activity of HIF2A, we did not investigate its potential role in this study.

Minor comments

1- In the Introduction, the authors wrote "Although initially effective, many patients relapse ". Many being unspecific, could the authors be a little more specific?

Response: After prostatectomy, about 30% of the patients will relapse and develop biochemical recurrence, and almost all metastatic patient will become resistant to castration (Rebello *et al*, 2021). This has been updated in the manuscript (lines 60-61).

2- Same remark for "improves survival of CRPC patients by several months". About 5 months of improvement in the latest clinical trials.

Response: To clarify this point, we modified the text line 71-73 and it now reads: *"Indeed, a combination of second- generation AR pathway inhibitors (e.g. apalutamide, enzalutamide) and conventional taxane-based chemotherapy (e.g. docetaxel) only improves survival of CRPC patients by about five months (Fizazi et al, 2012)".*

3- Figure 1C should appear right after (Cpa3 and MCPT4).

Response: This is now added.

4- Via in italic.

Response: This is now corrected.

5- The list of 380 genes should be better organized and presented. The names of some genes are cut off.

Response: We have now provided a PDF file with a better organized table.

6- Figure legends and not Figures Legends.

Response: This has been corrected.

7- Figure 1: instead of Figure 1.:

Response: This has been modified.

8- Figure 1: AR, DLP and AGI should be defined.

Response: They are now defined in the figure legend.

9- Figure 4: 0.05 instead of 0,05

Response: This is now corrected.

Referee #2 (Comments on Novelty/Model System for Author):

The authors assert that the inhibition of Hif1 by genetic or pharmacological manipulation in mice tumors deficient for Pten resulted in a recover from the castration resistance disease. This is a significant finding and point out Hif1 as a new molecular target against CRPC. The experiments are well performed and the in vivo model provides a strong support for the applicability of their results to the human disease.

It is a relevant discovery and it might be applied to other tumor types where PTEN is deleted.

The paper would be greatly strengthened if similar evidences can be observed in human tumors.

Referee #2 (Remarks for Author):

The authors assert that the inhibition of Hif1 by genetic or pharmacological manipulation in mice tumors deficient for Pten resulted in a recover from the castration resistance disease.

This is a significant finding and point out Hif1 as a new molecular target against CRPC. This relevant discovery might be applied to other tumor types where PTEN is deleted.

Response: We thank the reviewer for commending our work, for finding our results significant and our discovery relevant.

We presented in our manuscript data performed on the human prostate cancer androgen-insensitive cell line C4-2B, which is PTEN-null. We now expand our analyses to include two additional human androgen-insensitive cell lines that lack androgen receptor expression, DU-145 and PC-3. The former has been shown to express PTEN, whereas the latter is PTEN-deficient. Both cell lines have been recently classified as castration-resistant with stem cell-like features due to their expression of TROP2 and CD44 (Tang *et al*, 2022), i.e. similar to the castration-resistant tumors in Pten$^{(i)pe-/-}$ mice. Our new data demonstrate that PX-478 treatment potently induces apoptotic signaling in these cell lines. Therefore, collectively, we show that HIF1A inhibition efficiently induces apoptosis in PTEN-deficient androgen-insensitive human prostate tumor cells (C4-2B and PC-3), as well as in those that express PTEN (DU-145). These results are now included in the manuscript (**Figures 4D and Fig EV3 B and C**), and described in the text (lines 228-231 and 281-285).

Referee #3 (Comments on Novelty/Model System for Author):

valuable GEMM of CRPC

Referee #3 (Remarks for Author):

This study focuses on mechanisms by which prostatic intraepithelial neoplasia (PIN) lesions (considered precursors to prostate adenocarcinoma) in Pten-KO mice persist following androgen deprivation (castration). The authors identified a subpopulation of cells termed Luminal-C cells in Pten-KO mice and that this subpopulation is resistant to androgen deprivation. Droplet-based single-cell RNA-seq revealed that hypoxia-related genes were upregulated after castration. Using double KO mice (Pten and HIF1a) the authors showed that HIF1a prevents cell death after castration. This long-term effect was observed up to five months after castration. PX-478 an orally available inhibitor of HIF1A effectively prevented castration resistance in Pten-KO mice. PX-478 administration after castration-resistant prostate cancer lesions were already established also reduced the number of PINs and adenocarcinoma. This manuscript demonstrates the importance of HIF1a in prostate cancer progression and nominates HIF1a as promising target for incurable CRPC.

Response: We thank the reviewer for his/her positive comments.

Specific comments:

• The authors should better highlight that Luminal C cells are increased in Pten(i)pe-/- vs WT mice, thereby allowing the reader to understand the importance of this sub-population in disease progression.

Response: This is now highlighted in lines 124-126: *"Luminal-C cells are a minor population in control prostates, and the predominant epithelial subset in Pten-deficient prostatic lesions (Abu El Maaty et al, 2021; Abu El Maaty et al., 2022)".*

• Fig. 1D. can changes in subpopulations be quantified? A graph showing this would be helpful.

Response: The proportions of the different cellular populations in prostates of sham-operated and castrated $Pten^{(i)pe-/-}$ mice determined by scRNA seq are now presented (Figure EV1F).

• Although Supple Fig 1E shows the two specific subpopulations (luminal-A and -B), the color coding in Fig 1D is confusing, since many shades are very similar, it would help if luminal A and C cells would be indicated by an arrow or another identifier.

Response: The color-coding in Fig 1D has been changed to allow a clear identification of the different clusters, and Luminal-A and -C cells are indicated.

• Fig 1E and G. Are these the top hits? Were apoptosis related gene sets upregulated in CTX vs sham?

Response: The pathways depicted in Fig. 1E and G are among the top 10 down- and up-regulated genes. A complete list of the top 10 pathways is now added in the manuscript as Table EV2. We clarified this in the manuscript, lines 133-136: *"As expected, enrichment analysis of the genes downregulated in luminal-C cells of castrated mice compared to those of sham-operated ones using the Molecular Signatures Database (MSigDB) identified "androgen response" among the top 10 downregulated pathways"…*

While apoptosis was indeed upregulated in CTX vs sham, the genes identified in this pathway included the prostate stem cell marker *Cd44*, the anti-apoptotic Bcl2-family member *Mcl1*, as well as the pro-inflammatory cytokine *Il1b*, which together with the lack of induction in CC3-positive epithelial cells in Pten-deficient prostates after CTX indicates the lack of apoptotic induction in such cells.

• Could the authors explain the x axis of graphs E and G (-log2 FDR)?

Response: -log2 FDR stands for -log2 false discovery rate. This is now added in the corresponding figure legend.

• The authors report PIN and adenocarcinoma in dorsolateral prostate (DLP) of castrated Pten(i)pe-/- mice. Were other prostate lobes affected as well?

Response: Tamoxifen-induced gene inactivation in PSA-CreERT2 transgenic mice is more efficient in the DLP, which is most analogous to the human peripheral zone where most prostate tumors arise, than in the ventral (VP) and anterior (AP) prostates (Ratnacaram et al. PNAS. 2008). Accordingly, Pten-loss induces PIN formation in almost all glands of the DLP of $Pten^{(i)pe-/-}$ mice, and to a lower extent in the other lobes.

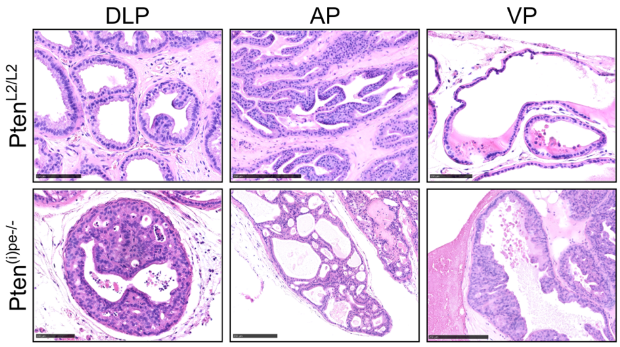

We thus report histological analyses on the DLP.

• Figure 2A and C - How does this compare to total weight of DLP and gland area in sham and castrated Pten(i)pe-/ mice?

Response: We report the weights of whole prostates to limit inaccuracies that may arise from the dissection of distinct lobes.

We have now included the prostate weight and gland area of sham-operated and castrated $Pten^{(i)pe-/-}$ mice in the manuscript (Fig EV1C), and describe the results in lines 101-104. As shown below, the prostate weight of $Pten^{(i)pe-/-}$ mice is decreased 1 month after castration, but remained higher than that of castrated $Pten/Hif1a^{(i)pe-/-}$ mice.

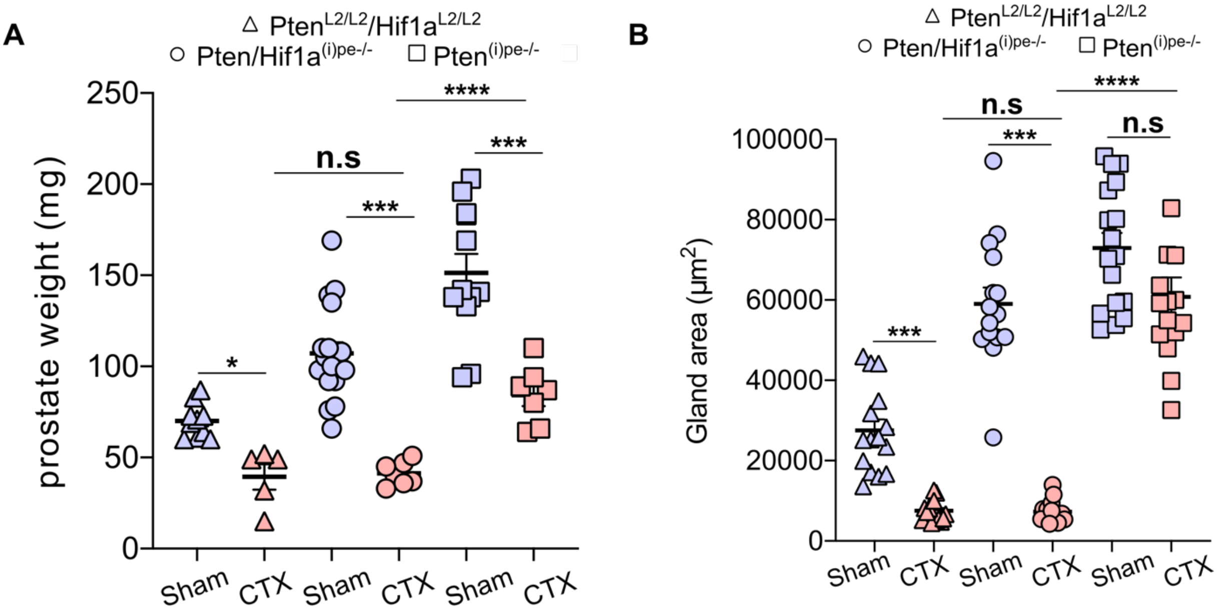

A. Prostate weights of $Pten^{L2/L2}/Hif1a^{L2/L2}$ (control), $Pten/Hif1a^{(i)pe-/-}$ and $Pten^{(i)pe-/-}$ mice, sham-operated or castrated at 3 months AGI and analyzed at one month after surgery. Data presented are mean ± SEM. Statistical analyses were performed using one-

way ANOVA followed by a post-hoc Tuckey test. n.s., *** and **** depict p-values ≥ 0.05 , < 0.001 and < 0.0001, respectively. N= 5-18 mice/condition.
B. Gland areas in the DLP of sham-operated and castrated Pten $^{L2/L2}$/Hif1a$^{L2/L2}$, Pten/Hif1a$^{(i)pe-/-}$ and Pten$^{(i)pe-/-}$ mice. Statistical analyses were performed using one-way ANOVA followed by a post-hoc Tuckey test. N=3-4 mice/condition. Five glands per mouse were quantified. n.s., *** and **** depict p-values ≥ 0.05, < 0.001 and < 0.0001, respectively.

As castration does not impact the abundance of PINs and adenocarcinoma in prostates of Pten$^{(i)pe-/-}$ mice, and does not significantly reduce the DLP gland area in them either, in contrast to that of control or Pten/Hif1a$^{(i)pe-/-}$ mice, the decrease in prostate weight of Pten$^{(i)pe-/-}$ mice by castration most probably results from reduced prostatic fluid content, which is a major androgen-dependent effect.

• Fig 2 and 3 - when referring to prostate weight, is this only DLP or the whole prostate?

Response: The prostate weight refers to the whole prostate and not specific lobes, as indicated in the figure legends.

• Could authors explain the staining of cleaved caspase 3 in the lumen of the acini (Figure 2G)?

Response: The time-course studies presented in figure 2G demonstrate a time-dependent increase in the number of CC3-positive cells, and a concurrent loss in the stratification of the epithelium in glands of Pten/Hif1a$^{(i)pe-/-}$ mice. Moreover, we provide similar results by cleaved PARP (CPARP) immunohistochemical analyses in Fig EV2F. At day 3, CC3- and CPARP-positive cells and debris are mainly in the lumen of the glands (i.e. the site of epithelial shedding).

• Detection of other apoptotic markers and proliferation markers would strengthen the CC3 IHC (and WB in C4-2B) results.

Response: As suggested, we performed immunodetection of cleaved-PARP (CPARP) that confirms our previous results with CC3, and determined the proliferation index of prostatic epithelial cells in Pten$^{(i)pe-/-}$ and Pten/Hif1a$^{(i)pe-/-}$ mice at multiple timepoints after castration.
This is now added in the manuscript lines 178-190:

'Although AR was already cytoplasmic 1 day after castration and the weight of the seminal vesicles was reduced after 3 days in both mouse lines (Fig EV2 A-C), histological analyses revealed tumor regression in prostates of Pten/Hif1a$^{(i)pe-/-}$ mice but not in those of Pten$^{(i)pe-/-}$ ones (Fig 2F). Furthermore, the proliferation rate of epithelial cells was decreased in both mouse lines 1-3 days after castration (Fig EV2D and E). Note however that while cell proliferation further decreased 1 month after castration in Pten/Hif1a$^{(i)pe-/-}$ mice, it increased in Pten$^{(i)pe-/-}$ mice to reach levels similar to those before castration (Fig EV2D and E). Remarkably, castration induced a time-dependent increase in the number of cleaved caspase-3 (CC3) and cleaved poly(ADP-ribose) polymerase (CPARP)-positive epithelial cells within 3 days in prostates of Pten/Hif1a$^{(i)pe-/-}$ mice, but not in those of Pten$^{(i)pe-/-}$ ones (Fig 2G and H, and Fig EV2 F-H).'

Furthermore, we determined cleaved-PARP levels as an additional marker of apoptosis in our western blot (Figure 4D).

• Why were such high concentrations of enzalutamide used for the organoid experiments? Experiments with an additional AR antagonist could rule out potential off-target effects.

Response: The concentrations used in our experiments are similar to those recently reported (Chan et al. Science. 2022; Deng et al. Nat Cancer. 2022), where prostatic organoids and prostate cancer cell lines were treated with enzalutamide at concentrations in the micromolar range. Additionally, a recent study (Smith et al. doi.org/10.1038/s41598-020-78798-x) screened the response of a panel of prostate cancer cell lines to enzalutamide, and found that the doses needed to inhibit 50% of the growth (GI50) after 72h of treatment were in the micromolar range. As enzalutamide is a highly potent and selective second-generation AR pathway inhibitor that is in widespread clinical use, we did not use additional AR antagonists.

• Panel 3 shows that Pten/HIF1a KO response to castration is maintained for long periods of time, could this be reverted by testosterone administration?

Response: The aim of our study was to discover novel targets for the management of CRPC. We identified HIF1A as a candidate to sensitize castration-resistant tumors to androgen deprivation, and validated its role in-vivo. The administration of testosterone to castrated Pten/Hif1a$^{(i)pe-/-}$ mice may lead to disease recurrence, but since this is not the aim of our work, and in adherence to the principles of the 3Rs of animal experimentation, we did not explore this avenue.

• It is unclear why enzalutamide-sensitive C4-2B cells don't respond to enzalutamide treatment with increased CC3.

Response: C4-2B cells are an androgen-insensitive subline of the LNCaP-derived C4-2 cells that resist chronic enzalutamide treatment by upregulating the expression of AR-FL and AR-V7 (Kohrt et al. doi.org/10.1158/1535-7163.MCT-20-0244; Liu et al. doi.org/10.1158/1078-0432.CCR-13-3296).

• How do authors speculate that HIF1A is activated in C4-2B in normoxic conditions?

Response: While hypoxia stabilizes HIF1A, the protein can also be detected by western blotting in PCa cells under normoxic conditions (e.g. Grebhardt et al. Int J Cancer. 2012; Abu el Maaty et al. Sci Adv. 2022). The reasons underlying increased HIF1A levels in PCa cells under normoxic conditions remain to be resolved (Ranasinghe et al. J Urol. 2015), but a study has suggested enhanced HIF1A mRNA translation in PC-3 cells (Ranasinghe et al. PLoS One. 2013).

• In the discussion, the authors argue that androgen deprivation promotes an immunosuppressive environment. Can this be further clarified? RNA-seq data on luminal C cells shows that castration induces upregulation of inflammation-related pathways and expression of certain pro-inflammatory cytokines is higher in CTX vs Sham.

Response: This is now further clarified in lines 248-250: *"Furthermore, we found that it triggers inflammation-related pathways, increases the expression of cytokines in such lesions and enhances the prostatic infiltration of MDSCs, thus promoting an immunosuppressive environment."*

• Is HIF1a overexpressed in Pten(i)pe-/- sham or castrated mice vs WT?

Response: Yes, the induction of HIF1A expression due to hypoxia is a very early event in Pten-loss driven prostate tumorigenesis, and HIF1A levels, as well as those of its target genes, are significantly induced in Pten-deficient luminal cells compared to those of control mice (Abu el Maaty et al. Sci Adv. 2022). Moreover, our scRNA-seq data show that Hif1a levels are induced in luminal-C cells of castrated Pten$^{(i)pe-/-}$ mice compared to sham-operated ones (Table EV1).

• Was mouse weight measured after PX-478 administration?

Response: In accordance with ethical guidelines, mice weights were measured, and less than 15% of body weight loss was observed after PX 478 administration.

• Impact would be enhanced if the authors can describe relevant correlates in human samples or cell lines

Response: We presented in our manuscript data performed on the human prostate cancer androgen-insensitive cell line C4-2B, which is PTEN-null. We now expand our analyses to include two additional human androgen-insensitive cell lines that lack androgen receptor expression, DU-145 and PC-3. The former has been shown to express PTEN whereas the latter is PTEN-deficient. Both cell lines have been recently classified as castration-resistant with stem cell-like features due to their expression of TROP2 and CD44 (Tang *et al.*, 2022), i.e. similar to the castration-resistant tumors in Pten$^{(i)pe-/-}$ mice. Our new data demonstrate that PX-478 treatment potently induces apoptotic signaling in these cell lines. Therefore, collectively, we show that HIF1A inhibition efficiently induces apoptosis in PTEN-deficient androgen-insensitive human prostate tumor cells (C4-2B and PC-3), as well as in those that express PTEN (DU-145). These results are now included in the manuscript (**Figures 4D and Fig EV3 B and C**), and described in the text (lines 228-231 and 281-285).

Abu El Maaty MA, Terzic J, Keime C, Rovito D, Lutzing R, Yanushko D, Parisotto M, Grelet E, Namer IJ, Lindner V *et al* (2022) Hypoxia-mediated stabilization of HIF1A in prostatic intraepithelial neoplasia promotes cell plasticity and malignant progression. *Sci Adv* 8: eabo2295

Barros-Silva JD, Linn DE, Steiner I, Guo G, Ali A, Pakula H, Ashton G, Peset I, Brown M, Clarke NW *et al* (2018) Single-Cell Analysis Identifies LY6D as a Marker Linking Castration-Resistant Prostate Luminal Cells to Prostate Progenitors and Cancer. *Cell Rep* 25: 3504-3518 e3506

Fizazi K, Scher HI, Molina A, Logothetis CJ, Chi KN, Jones RJ, Staffurth JN, North S, Vogelzang NJ, Saad F *et al* (2012) Abiraterone acetate for treatment of metastatic castration-resistant prostate cancer: final overall survival analysis of the COU-AA-301 randomised, double-blind, placebo-controlled phase 3 study. *Lancet Oncol* 13: 983-992

Rebello RJ, Oing C, Knudsen KE, Loeb S, Johnson DC, Reiter RE, Gillessen S, Van der Kwast T, Bristow RG (2021) Prostate cancer. *Nat Rev Dis Primers* 7: 9

Tang F, Xu D, Wang S, Wong CK, Martinez-Fundichely A, Lee CJ, Cohen S, Park J, Hill CE, Eng K *et al* (2022) Chromatin profiles classify castration-resistant prostate cancers suggesting therapeutic targets. *Science* 376: eabe1505

Wang X, Cai J, Zhao L, Zhang D, Xu G, Hu J, Zhang T, Jin M (2021) NUMB suppression by miR-9-5P enhances CD44(+) prostate cancer stem cell growth and metastasis. *Sci Rep* 11: 11210

9th Mar 2023

Dear Dr. Metzger,

Thank you for the submission of your revised manuscript to EMBO Molecular Medicine. We have now received the enclosed reports from the two referees who re-reviewed your study. As you will see, while referee #1 is supportive of publication, referee #3 raises a few remaining concerns. Therefore, we would like you to address these points in a minor round of revisions. More specifically, please address points 2, 3 and 4 as requested by the referee. Regarding point 1, while additional experiments would be welcome, they will not be required, and you may instead address this point with an adequate discussion in the manuscript text and in the point-by-point response.

Furthermore, please address the following editorial points:

1/ Main manuscript text:
- Would you consider making your manuscript a Report? This would not require any formatting changes from your end; we would simply switch the manuscript category.
- Please address the queries from our data editors in the data edited manuscript file. Please remove the yellow highlights, and only keep in track changes mode any new modification.
- Materials and methods:
o When referring to methods already published, please make sure the articles are open-access and/or to nevertheless provide minimal information.
o Animals: please indicate the housing and husbandry conditions (access to food/water and day/night cycle), as well as the origin of the mice.
- The Data Availability section should be moved to the end of the Materials and Methods. Please remove the sentence "All data needed to evaluate the conclusions of the study are present in the main text and the supplementary materials". Please make sure that the data are publicly available before publication of the manuscript.
- Acknowledgements/Funding information: Please make sure that the list of funders listed in the submission system match the information provided in the manuscript.
- Conflict of interest: Please indicate the full section title "Disclosure statement and competing interests".

2/ Figures:
- Figure 1a AR/Sham: The zoom box does not match the selection. Please double-check.
- Please provide Table 1 in an editable format.
- Table EV1 should be made Dataset EV1, ideally uploaded in excel format, and its legend should be removed from the manuscript text and added to the file. Table EV2 should consequently be renamed Table EV1, its legend removed from the manuscript and added to the table file.

3/ Source Data: please complete the checklist and provide the requested source data files.

4/ Thank you for providing a synopsis image. Please resize it as a PNG/TIF/JPEG file 550 px wide x 300-600 px high. Furthermore, please include a synopsis text. Synopses are displayed on the journal webpage and are freely accessible to all readers. They include a short stand first (maximum of 300 characters, including space) as well as 2-5 one-sentences bullet points that summarizes the paper. Please write the bullet points to summarize the key NEW findings. They should be designed to be complementary to the abstract - i.e. not repeat the same text. We encourage inclusion of key acronyms and quantitative information (maximum of 30 words / bullet point). Please use the passive voice. Please attach these in a separate file or send them by email, we will incorporate them accordingly.

5/ As part of the EMBO Publications transparent editorial process initiative (see our Editorial at http://embomolmed.embopress.org/content/2/9/329), EMBO Molecular Medicine will publish online a Review Process File (RPF) to accompany accepted manuscripts.
This file will be published in conjunction with your paper and will include the anonymous referee reports, your point-by-point response and all pertinent correspondence relating to the manuscript. Let us know whether you agree with the publication of the RPF and as here, if you want to remove or not any figures from it prior to publication.
Please note that the Authors checklist will be published at the end of the RPF.

I look forward to receiving your revised manuscript.

With kind regards,

Lise

Lise Roth, PhD
Senior Editor
EMBO Molecular Medicine

***** Reviewer's comments *****

Referee #1 (Comments on Novelty/Model System for Author):

As previously said, this is an interesting manuscript to read, with sufficient evidence from both mouse models and organoid samples to support their conclusions. THe experiments are well designed and executed. The conclusion will provide useful guidance to propose a more effective therapeutic treatment.

Referee #1 (Remarks for Author):

We thank the authors for answering the various questions with such precision. The article is more visible now and will undoubtedly shed new and interesting light on a rather underdeveloped area of prostate cancer, the role of HIF-1.

Referee #3 (Comments on Novelty/Model System for Author):

valuable GEMM of CRPC

Referee #3 (Remarks for Author):

Overall, the authors have addressed our suggestions and have accordingly modified the manuscript. Despite this, correlation with human samples or models should be further strengthened. Suggestions are detailed below:

1. Figure 1 shows that a population of epithelial cells, termed Luminal C, can resist castration. It's later suggested that this population drives androgen-independent tumor progression through HIF-1a signaling. These are novel results that help to understand resistant mechanisms to the standard of care for advanced prostate cancer i.e., androgen deprivation therapy (ADT). Validation of these results using human samples or cell lines is necessary to determine the relevance of these findings. Determining the levels of hypoxia genes after ADT in a panel of PC cell lines and genetic manipulation of HIF-1a to analyze growth effects before and after ADT are some of the experiments that should be considered.
2. The authors have added CPARP and CC3 western blot results on two independent CRCP cell lines which are concordant with the main findings of this article. Despite this, the concentration of PX-478 used in cell lines is noticeably high. Although pharmacological properties of the drug may require such high concentrations, controls with non-tumorigenic cell lines or rescue experiments are needed to rule out off-target effects.
3. Authors should cite Palayoor S.T. et al. Int J Cancer. 2008
4. The description of C4-2B needs to be corrected from "androgen insensitive" to "castration resistant", as these cells are known to express abundant and functional AR and respond to androgens.

Overall, the authors have addressed our suggestions and have accordingly modified the manuscript. Despite this, correlation with human samples or models should be further strengthened. Suggestions are detailed below:

Response: We are happy to see that the reviewer considers that we addressed his/her previous suggestions.

1. Figure 1 shows that a population of epithelial cells, termed Luminal C, can resist castration. It's later suggested that this population drives androgen-independent tumor progression through HIF-1a signaling. These are novel results that help to understand resistant mechanisms to the standard of care for advanced prostate cancer i.e., androgen deprivation therapy (ADT). Validation of these results using human samples or cell lines is necessary to determine the relevance of these findings. Determining the levels of hypoxia genes after ADT in a panel of PC cell lines and genetic manipulation of HIF-1a to analyze growth effects before and after ADT are some of the experiments that should be considered.

Response: We are pleased that the reviewer acknowledges that our findings shed light on a resistance mechanism in response to androgen deprivation therapy.
To enhance the human relevance of our findings, we selected the human castration-resistant cell lines C4-2B, DU-145 and PC-3. Using this panel of CRPC cell lines, we reported that the HIF1A inhibitor PX-478 triggers apoptotic signaling. We now show that HIF1A silencing in PC-3 cells has similar effects (Fig EV3D). Importantly, DU-145 and PC-3 have been recently identified as having stem cell-like properties on the basis of their expression of TROP2 (Tang et al. Science. 2022), and our in vivo studies showed that Luminal-C cells, which selectively express TROP2 and are castration resistant, are eliminated by apoptosis after androgen deprivation when HIF1A is genetically or pharmacologically inhibited. Hence, these results demonstrate that HIF1A is a potential target for the management of CRPC.

To further emphasize the clinical implications of our work, we added the following to the discussion section:
"Thus, these results are in line with our *in vivo* data showing that PX-478 sensitizes to castration Luminal C cells that also express such stem cell markers."
and
"These compelling findings therefore **highlight the importance of analyzing tumoral HIF1 signaling in patients whose disease progresses after ADT, and** pave the way for the clinical evaluation of HIF1A inhibitors in men with CRPC."

2. The authors have added CPARP and CC3 western blot results on two independent CRCP cell lines which are concordant with the main findings of this article. Despite this, the concentration of PX-478 used in cell lines is noticeably high. Although pharmacological properties of the drug may require such high concentrations, controls with non-tumorigenic cell lines or rescue experiments are needed to rule out off-target effects.

Response: The concentration of PX-478 used in our in-vitro experiments is in line with previous studies, including the study the reviewer mentions in point 3, as well as Koh et al. Mol Cancer Ther (2008). PX-478 might have off-target effects, but as silencing of HIF1A in PC-3 cells induces apoptotic signaling, we conclude that PX-478 pro-

apoptotic effects in human CRPC cell lines are elicited by targeting HIF1A. Moreover, our previous work demonstrated that therapeutic doses of PX-478 do not affect prostate weight and histology of WT mice (Abu el Maaty et al. Sci Adv. 2022), and importantly, this compound was well tolerated by patients with advanced cancers in a phase 1 clinical trial (DOI: 10.1200/jco.2010.28.15_suppl.3076 Journal of Clinical Oncology). Finally, we show that a combination of PX-478 and castration of Pten(i)pe-/- mice phenocopies the response of Pten/Hif1a(i)pe-/- mice to androgen deprivation, altogether demonstrating that PX-478's anti-tumor effects in CRPC cells are mediated by HIF1A inhibition.

3. Authors should cite Palayoor S.T. et al. Int J Cancer. 2008

Response: The reference is now added page 10 :
"… in keeping with previous results showing reduced survival of these cells in presence of PX-478 (Palayoor *et al*, 2008)."

4. The description of C4-2B needs to be corrected from "androgen insensitive" to "castration resistant", as these cells are known to express abundant and functional AR and respond to androgens.

Response: This is now changed.

20th Mar 2023

Dear Dr. Metzger,

Thank you for submitting your revised files. I am pleased to inform you that your manuscript is now accepted for publication and is being sent to our publisher to be included in the next available issue of EMM!

Please note that I introduced minor modifications in your synopsis text to fit our format, please let me know immediately if you don't agree with the following:

Resistance development to androgen deprivation therapy (ADT) in prostate cancer (PC) patients is a major clinical issue. By analysing PC mouse models, we found that HIF1A promotes androgen deprivation-induced luminal cell plasticity and that its inhibition overcomes ADT resistance.

- Luminal-C cells, the major epithelial subset in Pten-deficient tumors, are intrinsically castration-resistant.
- Single-cell analyses revealed that HIF1 signaling is further activated in luminal-C cells by castration, mediates a high plasticity state and induces resistance to androgen deprivation therapy.
- HIF1A inhibition sensitizes castration-resistant prostatic tumors to androgen deprivation and leads to long-term therapeutic responses.
- Androgen deprivation therapy combined with HIF1A inhibition is a promising approach to treat castration-resistant prostate cancer.

Please also be aware that the datasets deposited have to be public before online publication of the manuscript.

Congratulations on your interesting work,

With kind regards,

Lise

Lise Roth, PhD
Senior Editor
EMBO Molecular Medicine

Follow us on Twitter @EmboMolMed
Sign up for eTOCs at embopress.org/alertsfeeds

*** *** *** IMPORTANT INFORMATION *** *** ***

SPEED OF PUBLICATION
The journal aims for rapid publication of papers, using using the advance online publication "Early View" to expedite the process: A properly copy-edited and formatted version will be published as "Early View" after the proofs have been corrected. Please help the Editors and publisher avoid delays by providing e-mail address(es), telephone and fax numbers at which author(s) can be contacted.

Should you be planning a Press Release on your article, please get in contact with embomolmed@wiley.com as early as possible, in order to coordinate publication and release dates.

LICENSE AND PAYMENT:

All articles published in EMBO Molecular Medicine are fully open access: immediately and freely available to read, download and share.

EMBO Molecular Medicine charges an article processing charge (APC) to cover the publication costs. You, as the corresponding author for this manuscript, should have already received a quote with the article processing fee separately. Please let us know in case this quote has not been received.

Once your article is at Wiley for editorial production you will receive an email from Wiley's Author Services system, which will ask you to log in and will present you with the publication license form for completion. Within the same system the publication fee can be paid by credit card, an invoice, pro forma invoice or purchase order can be requested.

Payment of the publication charge and the signed Open Access Agreement form must be received before the article can be published online.

PROOFS

You will receive the proofs by e-mail approximately 2 weeks after all relevant files have been sent o our Production Office. Please return them within 48 hours and if there should be any problems, please contact the production office at embopressproduction@wiley.com.

Please inform us if there is likely to be any difficulty in reaching you at the above address at that time. Failure to meet our deadlines may result in a delay of publication.

All further communications concerning your paper proofs should quote reference number EMM-2022-17209-V3 and be directed to the production office at embopressproduction@wiley.com.

EMBO Press Author Checklist

Corresponding Author Name: Daniel Metzger	
Journal Submitted to: EMBO Molecular Medicine	
Manuscript Number: EMM-2022-17209	

USEFUL LINKS FOR COMPLETING THIS FORM
The EMBO Journal - Author Guidelines
EMBO Reports - Author Guidelines
Molecular Systems Biology - Author Guidelines
EMBO Molecular Medicine - Author Guidelines

Reporting Checklist for Life Science Articles (updated January 2022)
This checklist is adapted from Materials Design Analysis Reporting (MDAR) Checklist for Authors. MDAR establishes a minimum set of requirements in transparent reporting in the life sciences (see Statement of Task: 10.31222/osf.io/9sm4x). Please follow the journal's guidelines in preparing your manuscript.
Please note that a copy of this checklist will be published alongside your article.

Abridged guidelines for figures
1. Data
The data shown in figures should satisfy the following conditions:
- the data were obtained and processed according to the field's best practice and are presented to reflect the results of the experiments in an accurate and unbiased manner.
- ideally, figure panels should include only measurements that are directly comparable to each other and obtained with the same assay.
- plots include clearly labeled error bars for independent experiments and sample sizes. Unless justified, error bars should not be shown for technical replicates.
- if n<5, the individual data points from each experiment should be plotted. Any statistical test employed should be justified.
- Source Data should be included to report the data underlying figures according to the guidelines set out in the authorship guidelines on Data Presentation.

2. Captions
Each figure caption should contain the following information, for each panel where they are relevant:
- a specification of the experimental system investigated (eg cell line, species name).
- the assay(s) and method(s) used to carry out the reported observations and measurements.
- an explicit mention of the biological and chemical entity(ies) that are being measured.
- an explicit mention of the biological and chemical entity(ies) that are altered/varied/perturbed in a controlled manner.
- the exact sample size (n) for each experimental group/condition, given as a number, not a range;
- a description of the sample collection allowing the reader to understand whether the samples represent technical or biological replicates (including how many animals, litters, cultures, etc.).
- a statement of how many times the experiment shown was independently replicated in the laboratory.
- definitions of statistical methods and measures:
  - common tests, such as t-test (please specify whether paired vs. unpaired), simple χ2 tests, Wilcoxon and Mann-Whitney tests, can be unambiguously identified by name only, but more complex techniques should be described in the methods section;
  - are tests one-sided or two-sided?
  - are there adjustments for multiple comparisons?
  - exact statistical test results, e.g., P values = x but not P values < x;
  - definition of 'center values' as median or average;
  - definition of error bars as s.d. or s.e.m.

Please complete ALL of the questions below.
Select "Not Applicable" only when the requested information is not relevant for your study.

Materials

Newly Created Materials	Information included in the manuscript?	In which section is the information available? (Reagents and Tools Table, Materials and Methods, Figures, Data Availability Section)
New materials and reagents need to be available; do any restrictions apply?	Not Applicable	

Antibodies	Information included in the manuscript?	In which section is the information available? (Reagents and Tools Table, Materials and Methods, Figures, Data Availability Section)
For **antibodies** provide the following information:<br>- Commercial antibodies: RRID (if possible) or supplier name, catalogue number and or/clone number<br>- Non-commercial: RRID or citation	Yes	Materials and methods

DNA and RNA sequences	Information included in the manuscript?	In which section is the information available? (Reagents and Tools Table, Materials and Methods, Figures, Data Availability Section)
**Short novel DNA or RNA including primers, probes**: provide the sequences.	Not Applicable	

Cell materials	Information included in the manuscript?	In which section is the information available? (Reagents and Tools Table, Materials and Methods, Figures, Data Availability Section)
**Cell lines:** Provide species information, strain. Provide accession number in repository **OR** supplier name, catalog number, clone number, and/**OR** RRID.	Yes	Materials and methods
**Primary cultures:** Provide species, strain, sex of origin, genetic modification status.	Yes	Materials and methods
Report if the cell lines were recently **authenticated** (e.g., by STR profiling) and tested for mycoplasma contamination.	Yes	Materials and methods

Experimental animals	Information included in the manuscript?	In which section is the information available? (Reagents and Tools Table, Materials and Methods, Figures, Data Availability Section)
**Laboratory animals or Model organisms:** Provide species, strain, sex, age, genetic modification status. Provide accession number in repository **OR** supplier name, catalog number, clone number, **OR** RRID.	Yes	Materials and methods
**Animal observed in or captured from the field:** Provide species, sex, and age where possible.	Not Applicable	
Please detail **housing and husbandry conditions**.	Yes	Materials and methods

Plants and microbes	Information included in the manuscript?	In which section is the information available? (Reagents and Tools Table, Materials and Methods, Figures, Data Availability Section)
**Plants:** provide species and strain, ecotype and cultivar where relevant, unique accession number if available, and source (including location for collected wild specimens).	Not Applicable	
**Microbes:** provide species and strain, unique accession number if available, and source.	Not Applicable	

Human research participants	Information included in the manuscript?	In which section is the information available? (Reagents and Tools Table, Materials and Methods, Figures, Data Availability Section)
If collected and within the bounds of privacy constraints report on age, sex and gender or ethnicity for all study participants.	Not Applicable	

Core facilities	Information included in the manuscript?	In which section is the information available? (Reagents and Tools Table, Materials and Methods, Figures, Data Availability Section)
If your work benefited from core facilities, was their service mentioned in the acknowledgments section?	Yes	Acknowledgements section

Design

Study protocol	Information included in the manuscript?	In which section is the information available? (Reagents and Tools Table, Materials and Methods, Figures, Data Availability Section)
If study protocol has been **pre-registered, provide DOI in the manuscript**. For clinical trials, provide the trial registration number **OR** cite DOI.	Not Applicable	
Report the **clinical trial registration number** (at ClinicalTrials.gov or equivalent), where applicable.	Not Applicable	

Laboratory protocol	Information included in the manuscript?	In which section is the information available? (Reagents and Tools Table, Materials and Methods, Figures, Data Availability Section)
Provide DOI OR other citation details if **external detailed step-by-step protocols** are available.	Yes	Materials and methods

Experimental study design and statistics	Information included in the manuscript?	In which section is the information available? (Reagents and Tools Table, Materials and Methods, Figures, Data Availability Section)
Include a statement about **sample size** estimate even if no statistical methods were used.	Yes	Materials and methods
Were any steps taken to minimize the effects of subjective bias when allocating animals/samples to treatment (e.g. **randomization procedure**)? If yes, have they been described?	Yes	Materials and methods
Include a statement about **blinding** even if no blinding was done.	Yes	Materials and methods
Describe **inclusion/exclusion criteria** if samples or animals were excluded from the analysis. Were the criteria pre-established?<br><br>If sample or data points were omitted from analysis, report if this was due to attrition or intentional exclusion and provide justification.	Yes	Materials and methods
For every figure, are **statistical tests** justified as appropriate? Do the data meet the assumptions of the tests (e.g., normal distribution)? Describe any methods used to assess it. Is there an estimate of variation within each group of data? Is the variance similar between the groups that are being statistically compared?	Yes	Figure legends

Sample definition and in-laboratory replication	Information included in the manuscript?	In which section is the information available? (Reagents and Tools Table, Materials and Methods, Figures, Data Availability Section)
In the figure legends: state number of times the experiment was **replicated** in laboratory.	Yes	Figure legends
In the figure legends: define whether data describe **technical or biological replicates**.	Yes	

Ethics

Ethics	Information included in the manuscript?	In which section is the information available? (Reagents and Tools Table, Materials and Methods, Figures, Data Availability Section)
Studies involving **human participants**: State details of **authority granting ethics approval** (IRB or equivalent committee(s), provide reference number for approval.	Not Applicable	
Studies involving **human participants**: Include a statement confirming that **informed consent** was obtained from all subjects and that the experiments conformed to the principles set out in the WMA Declaration of Helsinki and the Department of Health and Human Services Belmont Report.	Not Applicable	
Studies involving **human participants:** For publication of **patient photos**, include a statement confirming that consent to publish was obtained.	Not Applicable	
Studies involving experimental **animals**: State details of **authority granting ethics approva**l (IRB or equivalent committee(s), provide reference number for approval. Include a statement of compliance with ethical regulations.	Yes	Materials and methods
Studies involving **specimen and field samples:** State if relevant **permits** obtained, provide details of authority approving study; if none were required, explain why.	Not Applicable	

Dual Use Research of Concern (DURC)	Information included in the manuscript?	In which section is the information available? (Reagents and Tools Table, Materials and Methods, Figures, Data Availability Section)
Could your study fall under dual use research restrictions? Please check biosecurity documents and list of **select agents and toxins** (CDC): https://www.selectagents.gov/sat/list.htm	Not Applicable	
If you used a select agent, is the security level of the lab appropriate and reported in the manuscript?	Not Applicable	
If a study is subject to dual use research of concern regulations, is the name of the **authority granting approval and reference number** for the regulatory approval provided in the manuscript?	Not Applicable	

Reporting

The MDAR framework recommends adoption of discipline-specific guidelines, established and endorsed through community initiatives. Journals have their own policy about requiring specific guidelines and recommendations to complement MDAR.

Adherence to community standards	Information included in the manuscript?	In which section is the information available? (Reagents and Tools Table, Materials and Methods, Figures, Data Availability Section)
State if relevant guidelines or checklists (e.g., **ICMJE, MIBBI, ARRIVE, PRISMA**) have been followed or provided.	Not Applicable	
For **tumor marker prognostic studies**, we recommend that you follow the **REMARK** reporting guidelines (see link list at top right). See author guidelines, under 'Reporting Guidelines'. Please confirm you have followed these guidelines.	Not Applicable	
For **phase II and III randomized controlled trials**, please refer to the **CONSORT** flow diagram (see link list at top right) and submit the CONSORT checklist (see link list at top right) with your submission. See author guidelines, under 'Reporting Guidelines'. Please confirm you have submitted this list.	Not Applicable	

Data Availability

Data availability	Information included in the manuscript?	In which section is the information available? (Reagents and Tools Table, Materials and Methods, Figures, Data Availability Section)
Have **primary datasets** been deposited according to the journal's guidelines (see 'Data Deposition' section) and the respective accession numbers provided in the Data Availability Section?	Yes	Data Availability Section
Were **human clinical and genomic datasets** deposited in a public access-controlled repository in accordance to ethical obligations to the patients and to the applicable consent agreement?	Not Applicable	
Are **computational models** that are central and integral to a study available without restrictions in a machine-readable form? Were the relevant accession numbers or links provided?	Not Applicable	
If publicly available data were reused, provide the respective **data citations in the reference list**.	Not Applicable	
